# Targeting regulated cell death pathways in cancers for effective treatment: a comprehensive review

**DOI:** 10.3389/fcell.2024.1462339

**Published:** 2024-11-15

**Authors:** Ruchi Saxena, Craig M. Welsh, You-Wen He

**Affiliations:** ^1^ Department of Integrative Immunobiology, Duke University School of Medicine, Durham, United States; ^2^ Department of Molecular Biology and Biochemistry, Sue and Bill Gross Stem Cell Research Center, University of California, Irvine, United States

**Keywords:** regulated cell death pathway, cancer therapy, apoptosis, autophagy, necroptosis, ferroptosis, pyroptosis, cuproptosis

## Abstract

Cancer is a complex disease characterized by specific “mission-critical” events that drive the uncontrolled growth and spread of tumor cells and their offspring. These events are essential for the advancement of the disease. One of the main contributors to these events is dysregulation of cell death pathways—such as apoptosis, necroptosis, ferroptosis, autophagy, pyroptosis, cuproptosis, parthanatos and—allows cancer cells to avoid programmed cell death and continue proliferating unabated. The different cell death pathways in cancers provide useful targets for cancer treatment. This review examines recent progresses in the preclinical and clinical development of targeting dysregulated cell death pathways for cancer treatment. To develop effective cancer therapies, it is essential to identify and target these mission-critical events that prevent tumor cells from timely death. By precisely targeting these crucial events, researchers can develop therapies with maximum impact and minimal side effects. A comprehensive understanding of the molecular and cellular mechanisms underlying these regulated cell death pathways will further the development of highly effective and personalized cancer treatments.

## 1 Introduction

**FIGURE 1:**
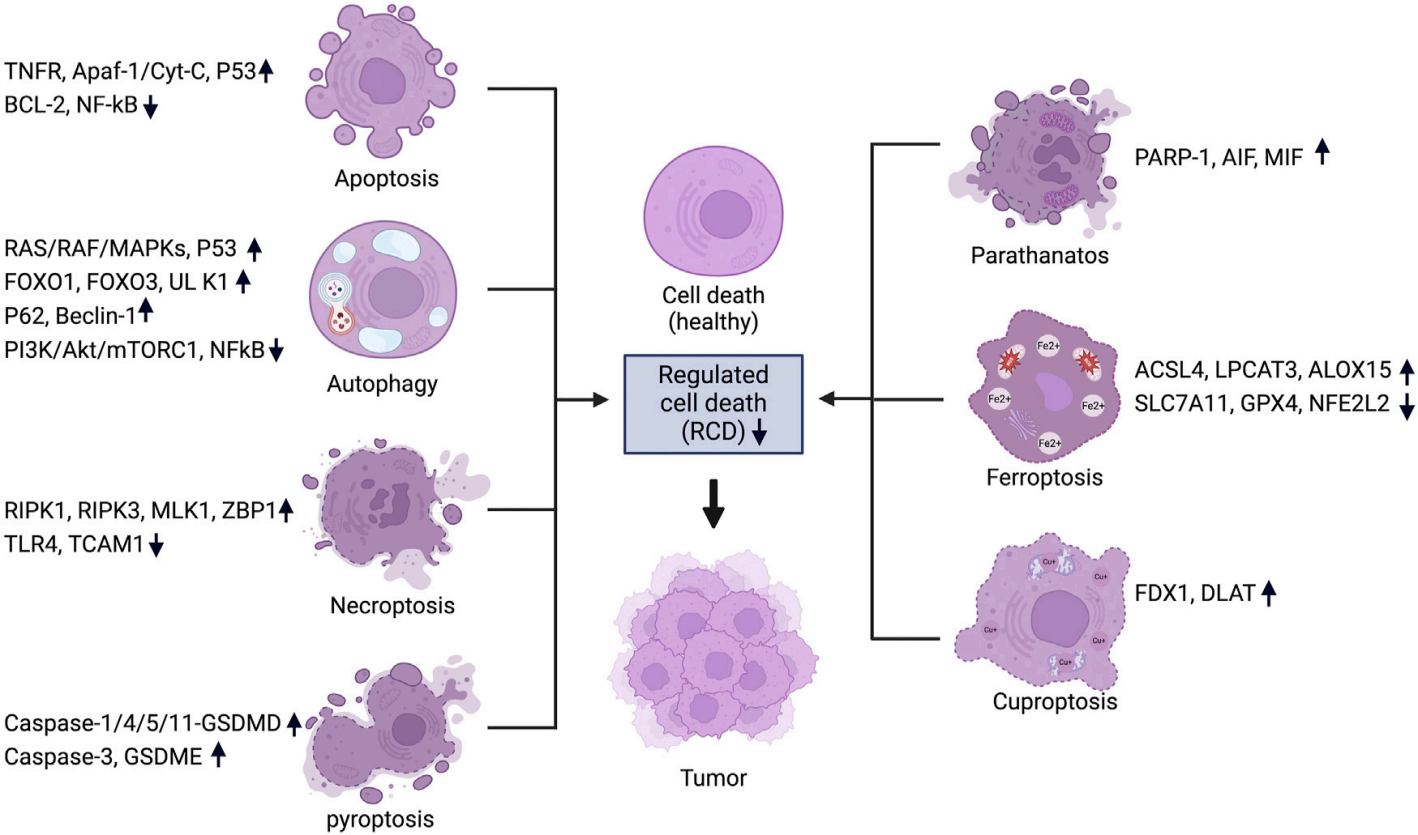
Regulated cell death pathways in cancer and their associated genes. For each RCD pathway, a set of key regulators are listed and the change of expression levels are indicated. Figure created using Biorender.

**FIGURE 2:**
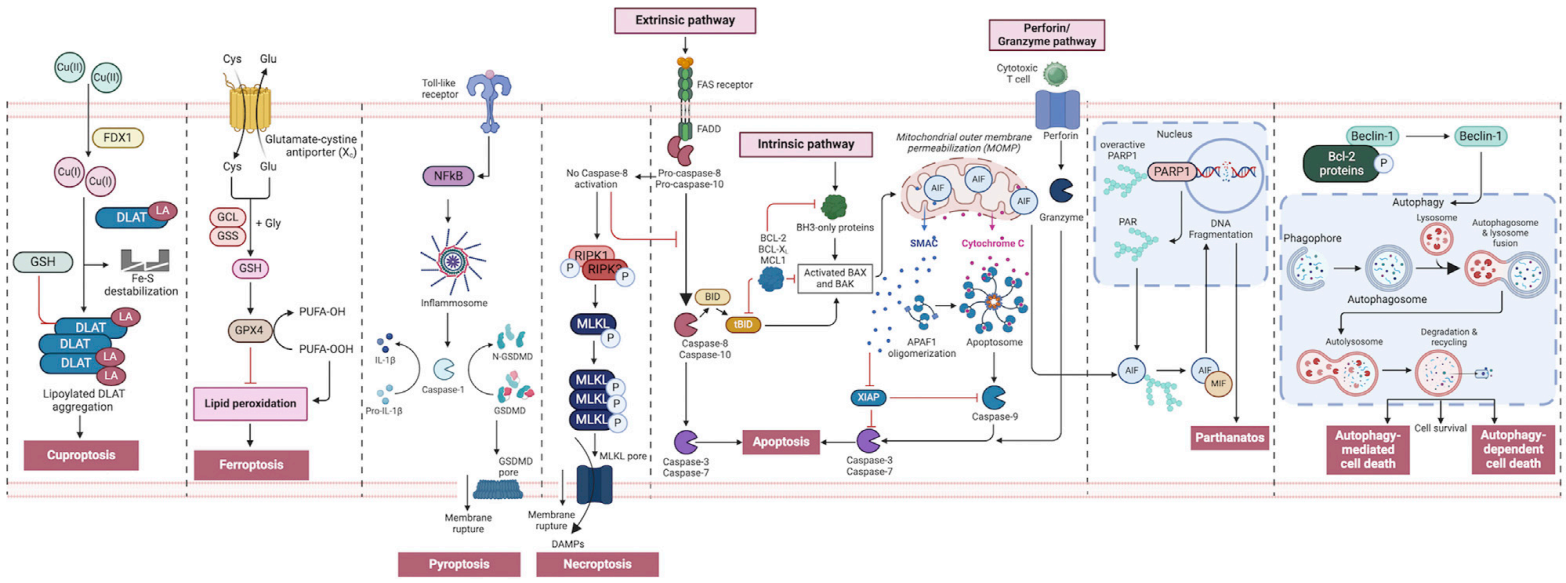
An overview of regulated cell death processes. A summary of the RCD pathways involved in cancer pathogenesis. Intrinsic apoptosis: Following an inherent fatal signal, BH3-only proteins activate BAX and BAK either directly or indirectly by binding to and blocking BCL-2 proteins. The mitochondrial outer membrane is then permeabilized (MOMP), releasing cytochrome C (Cyt C) and SMAC, the latter of which can suppress apoptosis. The apoptosome is subsequently produced, which activates caspase-9, followed by caspases 3 and 7, and initiates apoptosis. Extrinsic apoptosis: When death receptors (TNFR1, FAS, or TRAIL-R) receive an extrinsic fatal signal, they join with pro-caspase-8 and -10 to create complex I. Complex IIa is then generated, resulting in caspase-8 and -10 activation. Apoptosis is then initiated either directly by cleaving caspases-3 and -7, or indirectly by cleaving BID into tBID and activating BAX and BAK. Granzyme pathway: Cytotoxic T-cells are the main controllers for the granzyme pathway, which results in caspase-10 activation that in turn activates caspase-3. Granzyme B can activate caspases in the targeted cell. Necroptosis occurs when an extrinsic fatal signal is received but caspase-8 is not activated. Complex IIb (also known as the necrosome) is generated. This causes RIPK 1 and 3 to phosphorylate and activate mixed lineage kinase domain-like pseudokinase (MLKL). MLKL then forms a complex, causing the release of cytokines, chemokines, and damage-associated molecular patterns (DAMPS). Finally, this causes inflammation and necroptosis of the cell. Pyroptosis occurs when toll-like receptors (e.g., TLR4) detect an external fatal signal. Nuclear factor kappa B (NF-KB) signaling is initiated. This causes inflammasome development and subsequent caspase-1 activation. Then, pro-IL-1b is converted to active IL-1b, and gasdermin D (GSDMD) is broken down into N-GSDMD fragments resulting in inflammation and pyroptosis of the cell. Parthanatos occurs when an inherent fatal signal arises (for example, high reactive oxygen species accumulation), poly [ADP-ribose] polymerase 1 (PARP-1) is activated. Overactivation of PARP-1 can result in the accumulation of PAR polymer and the translocation of apoptosis inhibitory factor (AIF) from the mitochondria. AIF forms a compound with macrophage migration inhibitory factor (MIF) and re-enters the nucleus. Ultimately, this leads to cell parthanatos and DNA fragmentation. Autophagic Cell Death: Beclin-1 generally forms a complex with Bcl-2 proteins. After they have been phosphorylated and inactivated, free Beclin-1 can start autophagy. Ferroptosis occurs exclusively when there is an imbalance in the regulatory system, leading to the accumulation of lipid peroxide to a lethal threshold. Transferrin (TF) binds to extracellular Fe3+ and facilitates its transport into cells via transferrin receptor 1 (TfR1), where it is then converted to Fe2+. Later, intracellular divalent metal transporter 1 (DMT1) and zinc transporter 8/14 (ZIP8/14) store the Fe2+ in the intracellular labile iron pool (LIP). Fe2+ transfers electrons through the Fenton reaction with peroxide, resulting in the production of oxidizing free radicals. Following an excessive accumulation of iron within cells, numerous free radicals interact with polyunsaturated fatty acids (PUFA) found in the phospholipids of cell membranes resulting in the formation of lipid peroxides, which ultimately lead to cell death. The intracellular antioxidant stress system depends on GPX4 to eliminate surplus lipid peroxides. The Cystine/glutamate antiport (system xc−) facilitates the movement of glutamate from within cells to the outside, while simultaneously transporting cystine from the outside into cells. Cuproptosis: FDX1 plays a crucial role as a copper ion carrier in the induction of cell death and is involved in the regulation of protein lipoylation. Elevated copper levels foster the accumulation and functional impairment of lipoylated proteins, leading to instability of iron–sulfur cluster proteins, protein toxicity stress, and ultimately cell death. In addition, excessive copper binds to lipoylated DLAT, triggering abnormal oligomerization of DLAT and the formation of DLAT foci. This process contributes to cellular protein toxicity stress, further exacerbating cell death. Figure created using Biorender.

Cancer, an intricate disease characterized by uncontrolled cell proliferation and evasion of regulated cell death mechanisms, is a significant global health concern (Brown et al., 2023; Bhat et al., 2024). Among the several cellular mechanisms disrupted in cancer, the regulation of cell death pathways is crucial (Peng et al., 2022; Tong et al., 2022; Gong et al., 2023; Hadian and Stockwell, 2023). Programmed cell death (PCD), also known as regulated cell death (RCD), is a genetically controlled process in which cells die in an orderly manner (Koren and Fuchs, 2021; Gong et al., 2023). RCD encompasses several mechanisms, including apoptosis, necroptosis, autophagy and the newly identified pathways of pyroptosis, ferroptosis, cuproptosis, and parthanatos (Galluzzi et al., 2018) (Figure 1). Each of these mechanisms is crucial for maintaining cellular balance and responding to cellular stress (Tang et al., 2019; Lamichhane and Samir, 2023). When mammalian cells experience irreversible disruptions in their internal or external milieu, they can initiate several signal transduction cascades that ultimately result in cell death (Kayagaki et al., 2024; Newton et al., 2024). In cancer, the disruption of these pathways not only enables the initiation and progression of tumors but also significantly affects treatment resistance and patient outcomes (Table 1) (Gong et al., 2023). Each of these RCD patterns is triggered and propagated through molecular pathways that exhibit significant connectivity (Tang et al., 2019) (Figure 2). Each variant of RCD exhibits a diverse array of morphological characteristics, ranging from complete to partial programmed cell death, which elicit unique immunomodulatory properties, including anti-inflammatory effects, promotion of immune tolerance, enhancement of inflammation, and immunogenicity. Apoptosis, marked by regulated cell shrinkage and membrane blebbing, typically leads to anti-inflammatory outcomes since apoptotic cells are phagocytosed without provoking immune activation (Elmore, 2007). Autophagy is a process of cellular degradation that generally promotes cell survival; however, under prolonged stress, it can result in cell death. Autophagy can either suppress or promote inflammation based on the context, as it regulates the immune response by degrading immune modulators or releasing signals that activate immune cells (Liu et al., 2023). Necroptosis, characterized by membrane rupture and the release of cellular contents, triggers inflammation by activating immune cells via damage-associated molecular patterns (DAMPs) (Kaczmarek et al., 2013). In a similar manner, pyroptosis, characterized by pore formation and cell lysis, enhances inflammation through the release of pro-inflammatory cytokines such as IL-1β (Liu Y. et al., 2024). Cuproptosis, a form of cell death that relies on copper, inflicts damage on the mitochondria and has the potential to trigger immune responses, although its specific immunomodulatory characteristics are still under investigation (Springer et al., 2024). Ferroptosis, initiated by iron-dependent lipid peroxidation, has the potential to promote inflammation via the release of DAMPs, which in turn can affect immune responses (Qi and Peng, 2023). Parthanatos, resulting from excessive PARP activation that leads to significant DNA damage, can trigger inflammation while potentially fostering immune tolerance in chronic conditions (Huang et al., 2022). In summary, these RCD pathways influence immune dynamics by either inhibiting or facilitating immune activation, thereby affecting cancer progression and treatment results. (Galluzzi et al., 2018; Liao M. et al., 2022).

**TABLE 1:** Role of RCD pathways in cancer.

Dysregulated pathway	Cancer type	Dysregulated gene/mechanism	Mechanism of chemoresistance	References
Apoptosis	Pancreatic cancer, ovarian cancer, lymphoma, multiple myeloma, lung adenocarcinoma, prostate cancer	Overexpression of anti-apoptotic BCL2 family proteins	Resistance to apoptosis through BCL2	Lowe and Lin (2000), Walensky et al. (2004), Del Gaizo Moore et al. (2007), Mohammad et al. (2015)
Apoptosis	Non-Small Cell Lung Cancer	Overexpression of Bcl-2 and downregulation of Bax	Inhibits chemotherapy-induced apoptosis, leading to cisplatin resistance	Alam et al. (2022)
Apoptosis	Chronic Lymphocytic Leukemia	Overexpression of Bcl-2 family proteins	Inhibition of apoptosis leading to fludarabine resistance	Tzifi et al. (2012)
Apoptosis	Prostate Cancer	P53 mutation	Impaired apoptotic response causing docetaxel resistance	Gan et al. (2011)
Apoptosis	Colon Cancer	CSC overexpress death receptors (DR4, DR5)	Increased resistance to apoptosis, reduced sensitivity to TRAIL therapy	Sussman et al. (2007)
Apoptosis	Breast cancer	Chemoresistant CSC-like population with elevated FAS, DR5	Resistance to apoptosis, potential therapeutic targeting with FAS ligand	Wang and Scadden (2015)
Apoptosis	solid tumors and myeloid leukemias	Upregulation of XIAP, inhibition of CASP3/7	Resistance to apoptosis in the presence of apoptotic signals	Tamm et al. (2000), Fulda and Vucic (2012), Monian and Jiang (2012), Amaravadi et al. (2015), Yang et al. (2019)
Apoptosis	hematological malignancies, melanoma, testicular germ cell tumor, hepatocellular carcinoma, breast cancer, urothelial carcinoma, ovarian cancer	Amplification/overexpression of MCL1	Supresses mitochondrial pro-apoptotic proteins	Wertz et al. (2011), Carneiro and El-Deiry (2020), Satta and Grant (2020), Wei et al. (2020), Widden and Placzek (2021), Li et al. (2024a)
Autophagy	Breast cancer	Sustained JNK activation, Beclin1 release, p62 accumulation	Autophagy linked to cellular senescence,	Choi et al. (2020)
Autophagy	Glioblastoma	CDK4 downregulation	Impairs autophagy	Giordano et al. (2023)
Autophagy	Ovarian Cancer	Downregulation of ATG14, FOXP1	Enhances sensitivity to cisplatin, autophagy promotes chemoresistance	Hu et al. (2020)
Autophagy	Hepatocellular carcinoma	P62 accumulation	preventing oncogene-induced senescence and death of cancer-initiating cells	Taniguchi et al. (2016)
Autophagy	Lung cancer, Liver cancer, myeloma	Decreased Beclin-1 and P62 accumulation	P62 accumulation leads to altered NF-κB and inflammation signaling	Park et al. (2013), Tucci et al. (2014)
Autophagy	Non-Small Cell Lung Cancer	ATG3 upregulation	ATG3 upregulation weakened miR-1-induced apoptosis in cisplatin-resistant non-small cell lung cancer (NSCLC) cells	Hua et al. (2018)
Necroptosis	Breast Cancer, Colorectal cancer, Ovarian cancer, Acute myeloid leukemia (AML), Melanoma	downregulation of RIPK3 expression	Reduced necroptosis leads to poor survival and chemoresistance	Feng et al. (2015), Koo et al. (2015), Wang et al. (2020), Morgan and Kim (2022)
Necroptosis	AML (with Sorafenib use)	Sorafenib inhibits MLKL phosphorylation in SMAC mimetic-induced necroptosis	Reduces sensitivity to necroptosis, maintaining chemoresistance	Feldmann et al. (2017)
Necroptosis	Colorectal cancer	Metabolic reprogramming and hypoxia reduce RIPK1/RIPK3 expression	Anaerobic glycolysis and pyruvate scavenging of ROS confer necroptosis resistance	Huang and Yu (2015)
Pyroptosis	Melanoma, HER2+ Breast Cancer	Low GSDME/GSDMB expression	Impairs pyroptosis, resulting in decreased immune cell infiltration	Lage et al. (2001), Hergueta-Redondo et al. (2016), Molina-Crespo et al. (2019)
Ferroptosis	Lung Cancer	Overexpression of SLC7A11 activated by SOX2	Increases resistance to ferroptosis through cystine transport	Wang et al. (2021c)
Ferroptosis	Colorectal Cancer	p53 boosts antioxidant defenses	Limits ferroptosis, contributing to therapy resistance	Xie et al. (2017)
Ferroptosis	Breast Cancer	GPX4 overexpression	Prevents lipid peroxidation, leading to resistance to chemotherapy	Li et al. (2021)
Ferroptosis	Ovarian Cancer	Dysregulation of SLC7A11/High GPX4 activity	Inhibits ferroptosis, aiding in chemoresistance	Qin et al. (2023), Fantone et al. (2024)
Ferroptosis	Pancreatic Cancer	High GPX4 activity	Protects against ferroptosis	Ye et al. (2021b)
Ferroptosis	Prostate Cancer, Melanoma, Sarcoma	Altered iron metabolism and Fenton reaction	Promotes resistance to oxidative stress, preventing ferroptosis	Piccolo et al. (2024)
Ferroptosis	Glioblastoma	Upregulation of antioxidant pathways independent of GPX4	Blocks ferroptosis, supporting survival against oxidative stress	Hangauer et al. (2017)
Cuproptosis	Prostate Cancer Breast Cancer	Abnormal copper regulation and elevated serum copper levels	Copper promotes metastasis and resistance through metabolic activation	Safi et al. (2014), Blockhuys et al. (2017), Blockhuys and Wittung-Stafshede (2017)
Cuproptosis	Lung Adenocarcinoma, Glioma	High-risk group associated with immune escape through cuproptosis-related lncRNAs	Reduces response to immunotherapy	Ma et al. (2022), Wang et al. (2022b), Wang et al. (2022c)
Parthanatos	Breast Cancer, Ovarian cancer, oral cancer	Overexpression of PARP-1	Enhanced chemoresistance	Harraz et al. (2008), Fong et al. (2009), Galia et al. (2012), Dorsam et al. (2018), Pazzaglia and Pioli (2019), Wang et al. (2021a)

Targeting various RCD pathways to treat cancer has been under intensive investigation for several decades (Peng et al., 2022). Research in the last decade has revealed novel RCD pathways and with these discoveries, progress has been made in clinical application to target these newly identified pathways for cancer treatment (Man et al., 2017; Seehawer et al., 2018; Zhou et al., 2021; Wang Y. et al., 2022; Zhang C. et al., 2022). Furthermore, therapeutic approaches that target these RCD pathways have been used in combination with immunotherapeutic agents to further enhance their efficacies (Tong et al., 2022). Such combined approaches have the potential to significantly improve patient outcomes. Despite notable advancements, major challenges such as treatment resistance exist. This review summarizes recent advancement in preclinical and clinical development to target RCD pathways in cancer from a therapeutic standpoint, exploring how alterations in these mechanisms contribute to cancer development and impact the efficacy of current treatment methods.

### 1.1 Dysregulated apoptosis in cancer and targeting strategies for therapy

Apoptosis is a vital intracellular mechanism that maintains tissue homeostasis in an organism by regulating cell populations (Elmore, 2007; Akhtar and Bokhari, 2024) (Figure 1). However, in cancer, cells lose their capacity to undergo apoptosis-induced death, which results in unchecked cell proliferation (Morana et al., 2022). Therefore, targeting the regulation of the apoptosis signaling pathway can be one of the crucial methods to improve cancer treatment (Pfeffer and Singh, 2018). Apoptosis is characterized by cell shrinkage, chromatin condensation, membrane blebbing, DNA breakage, and apoptotic body formation (Elmore, 2007). It involves two primary pathways: the extrinsic pathway, triggered by death receptors, and the intrinsic pathway, regulated by mitochondria (Zhang et al., 2005; Jan and Chaudhry, 2019) (Figure 2). The extrinsic pathways are controlled by transmembrane death receptors belonging to the CD95 (Apo-1 or Fas)/TRAIL/tumor-necrosis factor (TNF) receptor 1 family. When death ligands such as TNFα (tumor necrosis factor-alpha), Fas ligand (FasL), or TRAIL bind to their corresponding cell surface receptors—TNFR1, Fas, and death receptors 4 and 5 (DR4/5)—it triggers a signaling cascade. This ligand-receptor interaction leads to the recruitment and activation of caspase-8, an initiator caspase, which in turn activates downstream effector caspases (Annibaldi and Walczak, 2020). The mitochondrion is involved in the other primary route that is responsible for death signaling. It performs the function of an integrating sensor of numerous death insults by releasing cytochrome c into the cytosol, where it then activates caspase. It is believed that the mitochondrial route is the primary target of survival signaling pathways (Elmore, 2007). The Bcl-2 family controls the mitochondrial (intrinsic) pathway, which is triggered by damage of the mitochondria and the subsequent release of cytochrome c. This route is initiated by cytotoxic agents and UV radiation. Cytochrome c, Apaf-1, d-ATP/ATP, and procaspase-9 interact to form an apoptosome, which then triggers the caspase cascade (Wang and Youle, 2009). Additionally, a third pathway related to endoplasmic reticulum (ER) stress has also been described (Iurlaro and Munoz-Pinedo, 2016). Stress causes mutant proteins to accumulate in the endoplasmic reticulum, disrupting the balance between protein folding and protein requirement. This event triggers the unfolded protein response (UPR), which identifies and modulates ER stress (Schroder and Kaufman, 2005; Gardner et al., 2013). Key sensors in the UPR—ATF6 (activating transcription factor 6), IRE1α (inositol-requiring enzyme 1 alpha), and PERK (protein kinase R-like ER kinase)—are activated when misfolded protein concentrations exceed a certain threshold. If the stress is too severe or prolonged, the UPR can shift from a protective role to triggering apoptosis, in order to eliminate the affected cell and prevent damage (Spencer and Finnie, 2020). Despite having distinct mechanisms of initiation, these intrinsic, extrinsic and stress-induced pathways all lead to activation of a series of proteolytic enzymes that are members of the caspase family (Elmore, 2007; McIlwain et al., 2015) (Figure 2). The caspases, which are cascades of cysteine aspartyl proteases are produced as dormant zymogens, which are then activated by proteolytic cleavage. This is normally accomplished by the action of upstream apical caspases (McIlwain et al., 2015). Apart from these intrinsic, extrinsic and stress-induced processes, there exists an additional pathway that entails T cell mediated cytotoxicity and perforin/granzyme-dependent cell death. The cell death inducing enzymes in this pathway are granzyme B and granzyme A proteases (Trapani and Smyth, 2002).

Cancer cells often overexpress proteins that prevent the apoptotic cascade from being activated, including Bcl-2 and related anti-apoptotic proteins such as Bcl-xL, Mcl-1, A1/Bf1 and Bcl-w (Table 1) (Lowe and Lin, 2000). Targeting these proteins has become a strategy to inhibit cancer proliferation and promote cell death (Frenzel et al., 2009; Carneiro and El-Deiry, 2020). Developing cancer drugs targeting the apoptosis pathway represents the first phase of clinical development in the field (Jan and Chaudhry, 2019). A comprehensive list of compounds targeting apoptotic pathways and demonstrating anti-cancer properties is presented in Table 2. ABT-737 was the initial chemical inhibitor targeting Bcl-2, Bcl-xL and Bcl-w (Del Gaizo Moore et al., 2007). It binds to the hydrophobic pocket of Bcl-2 family members and has shown efficacy against lung cancer, especially when combined with chemotherapy and radiation therapy. Navitoclax (ABT-263) demonstrates anti-cancer properties, particularly when used with MEK or tyrosine kinase inhibitors against solid tumors (Tse et al., 2008; Walensky et al., 2004) (ABT-199), a potent Bcl-2 inhibitor, has shown promising outcomes for treating acute myeloid leukemia (AML), chronic lymphocytic leukemia (CLL) and non-Hodgkin lymphoma (NHL) (Souers et al., 2013). Selective Bcl-xL inhibitors include a vaccine for prostate cancer and ABBV-155, an antibody-drug conjugate being studied as monotherapy or for use in combination with taxanes for solid tumors (Walensky et al., 2004).

**TABLE 2:** Apoptosis targeting drugs for cancer therapy.

Drug/Treatment	Mechanism of action	Cancer type	Clinical status	References
Venetoclax (ABT-199)	Selective BCL-2 inhibitor	Chronic lymphocytic leukemia, Acute myeloid leukemia	FDA Approved	DiNardo et al. (2019), Shimony et al. (2022)
Navitoclax (ABT-263)	BCL-2/BCL-XL inhibitor	Solid tumors, Hematological malignancies	Phase I/II	Tse et al. (2008), Gandhi et al. (2011), Rudin et al. (2012), Nor Hisam et al. (2021), Joly et al. (2022)
Obatoclax (GX15-070)	Pan-BCL-2 inhibitor	Hematological malignancies, Solid tumors	Phase I/II	Parikh et al. (2010), Paik et al. (2011), Goy et al. (2014)
ABT-737	BCL-2/BCL-XL/BCL-w inhibitor	Lung cancer, Hematological malignancies	Preclinical	Lee et al. (2022), Yuan et al. (2022), Yang et al. (2024)
Birinapant (TL32711)	SMAC mimetic (IAP antagonist)	Solid tumors, Hematological malignancies	Phase I/II	Amaravadi et al. (2015)
Nutlin-3	MDM2 inhibitor (p53 activator)	Leukemia, Solid tumors	Phase I	Secchiero et al. (2011)
RG7112	MDM2 inhibitor (p53 activator)	Sarcoma, myelogenous leukemia, hematologic neoplasms	Phase I	Andreeff et al. (2016)
Renieramycin T (RT)	Natural compound stabilizing p53	Lung cancer	Preclinical	Petsri et al. (2019)
Andrographolide (ANDRO)	Degrades mutant p53	Various cancers	Preclinical	Sato et al. (2018)
Protoporphyrin IX (PpIX)	Targets p53 and p73	Chronic lymphocytic leukemia	Preclinical	Son et al. (2019)
DJ34	Inhibits c-Myc, activates p53	Leukemia	Preclinical	Tadele et al. (2021)
AQ-101	MDM2 inhibitor	Leukemia	Preclinical	Gu et al. (2018)
Palbociclib	CDK4/6 inhibitor	Breast cancer	FDA Approved	Loi et al. (2022), Di Cosimo et al. (2023), Slamon et al. (2024)
Azacytidine	Hypomethylating agent	Myelodysplastic syndromes, Acute myeloid leukemia	FDA Approved	Mishra et al. (2023), Dohner et al. (2024) 11. Harper et al., 2023.
HO-3867	p53 agonist	Ovarian cancer	Preclinical	Devor et al. (2021)
SAHBA	Stapled peptide BH3 mimetic targeting BCL-XL	Leukemia	Preclinical	Chang et al. (2013)
SMBA1-3	Small molecules activating Bax	Various cancers	Preclinical	Li et al. (2017)
AMG 176 and AZD5991	MCL-1 inhibitor	Myeloma	Phase I	Caenepeel et al. (2018), Tron et al. (2018)
AM-8621	MCL-1 inhibitor	Hematological malignancies	Phase I	Wei et al. (2020)
VU661013 and S63845	MCL-1 inhibitor	Hematological malignancies,	Preclinical	Carneiro and El-Deiry (2020), Satta and Grant (2020)
LCL161	Oral SMAC mimetic (IAP antagonist)	Multiple myeloma, Breast cancer	Phase I/II	Yang et al. (2019)
Mapatumumab	TRAIL receptor agonist antibody (DR4)	Solid tumors	Phase II	Greco et al. (2008), Hotte et al. (2008), Trarbach et al. (2010), Younes et al. (2010), von Pawel et al. (2014), Ciuleanu et al. (2016)
Conatumumab	TRAIL receptor agonist antibody (DR5)	Solid tumors	Phase II	Herbst et al. (2010), Kindler et al. (2012)
Drozitumab	TRAIL receptor agonist antibody (DR5)	Solid tumors	Phase I/II	Rocha Lima et al. (2012)
APR-246 (Eprenetapopt)	Restores mutant p53 function	Myelodysplastic syndromes, Acute myeloid leukemia	Phase III	Lehmann et al. (2012), Cluzeau et al. (2021)
COTI-2	Restores mutant p53 function	Solid tumors	Phase I	Maleki Vareki et al. (2018), Lindemann et al. (2019), Nagourney et al. (2023), Tang et al. (2023)
Panobinostat	HDAC inhibitor	Multiple myeloma	FDA Approved	23. San-Miguel et al., *Lancet Oncology*, 2014.
ABBV-075 (Mivebresib)	BET inhibitor	Solid tumors, Hematological malignancies	Phase I	Kim et al. (2018)
Mortaparib Plus	HSP70 inhibitor (p53 reactivator)	Colorectal cancer, Breast cancer	Preclinical	Sari et al. (2021)
ABBV-155 (Mirzotamab clezutoclax)	Antibody-drug conjugate targeting B7-H3	relapsed or refractory solid tumors	Phase I clinical trial	Walensky et al. (2004)

BH3 mimetics have been effectively created using stapled peptides that specifically bind through protein-protein interactions and have an improved ability to enter the cell (Ali et al., 2019). SAHBA (Stabilized Alpha-Helix of BCL-2 Domains) mimics the α-helical BH3 section of proapoptotic BID, efficiently enters leukemia cells, binds to Bcl-xL, and promotes apoptosis (Chang et al., 2013). Targeting Bax using small molecules like SMBA1-3, which bind directly to Bax and inhibit the phosphorylation of S184, promotes cytochrome c release and apoptosis (Li et al., 2017).

The Bcl-2 family member Mcl-1 can prevent apoptosis induced by multiple apoptotic triggers such as radiation and chemotherapy (Wertz et al., 2011; Widden and Placzek, 2021). AM-8621 attaches to the Mcl-1 binding pocket, displaces BIM and induces apoptosis in a myeloma cell line (Wei et al., 2020). Derivatives AMG 176 and AZD5991 have shown notable outcomes in combination with venetoclax and chemotherapy (Caenepeel et al., 2018; Tron et al., 2018). Mcl-1 inhibitors VU661013 and S63845 show promise in treating blood cancers and overcoming resistance to venetoclax when used in combination with other therapies (Carneiro and El-Deiry, 2020; Satta and Grant, 2020). In addition, IAP inhibitors have been used to target apoptosis in cancer (Fulda and Vucic, 2012; Monian and Jiang, 2012). Antagonists like LCL161 and birinapant (TL32711) show promising anti-tumor effects, particularly in combination with chemotherapy, radiation and the immune checkpoint inhibitor (ICI) anti-PD1 pembrolizumab (Amaravadi et al., 2015; Yang et al., 2019).

Agonist antibodies were also created targeting DR4 and DR5 due to their favorable half-life and notable preclinical efficacy (Hymowitz et al., 1999; LeBlanc and Ashkenazi, 2003). The only clinically tested anti-DR4 monoclonal antibody is mapatumumab, a completely human DR4-agonistic antibody with selective and strong binding to DR4 and high cytotoxicity (Pukac et al., 2005). Mapatumumab was tested in phase I and II clinical trials for HCC, NSCLC, colorectal cancer, and refractory non-Hodgkin’s lymphoma (Greco et al., 2008; Hotte et al., 2008; Trarbach et al., 2010; Younes et al., 2010; von Pawel et al., 2014; Ciuleanu et al., 2016), but none of the assays met the initial objectives, ending clinical development. Unlike DR4, several DR5 agonist antibodies have been developed and tested in clinic including Conatumumab, Drozitumab, Lexatumumab, LBY135, Tigatuzumab, and DS-8273a (Belyanskaya et al., 2007; Herbst et al., 2010; Kang et al., 2011; Forero-Torres et al., 2013; Burvenich et al., 2016; Dominguez et al., 2017; Forero et al., 2017). Conatumumab and Drozitumab demonstrated efficacy in advanced solid tumors, while Lexatumumab was tested in prostate and bladder cancer cells (Shimada et al., 2007; Herbst et al., 2010; Rocha Lima et al., 2012). DS-8273a is the newest clinically tested anti-DR5 antibody. The initial study showed that DS-8273a might be used to eliminate myeloid-derived suppressor cells in advanced cancer patients, but no objective response was seen (Dominguez et al., 2017). It is tested in three more clinical trials to assess its safety in advanced solid tumors and lymphomas or its efficacy in combination with Nivolumab in advanced colorectal cancer and unresectable stage II and IV melanoma (Dubuisson and Micheau, 2017). In addition, chimeric mouse–human antibodies LBY135 and Tigatuzumab were developed. Solid advanced cancers tolerated LBY135 well, and Tigatuzumab was investigated for relapsed lymphoma or solid malignancies (Forero-Torres et al., 2010; Sharma et al., 2014). Conatumumab and Drozitumumab reached phase II clinical trials, but Lexatumumab, LBY-135, and Tigatuzumab did not (Dubuisson and Micheau, 2017).

The p53 protein, a critical tumor suppressor, is often altered or deactivated in various malignancies, making it an ideal target for therapeutic treatments. Several p53-targeted medicines have been developed to help restore or improve p53 function. MDM2 inhibitors, including Nutlin-3, APG-115, RG7388, DS-3032, and MK-8242, suppress the p53-MDM2 interaction, stabilizing p53 and inducing apoptosis in malignancies such as gastric cancer and leukemia (Ding et al., 2013; Levine, 2022). Other MDM2 antagonists include AMG-232, HDM201, BI 907828, and ALRN-6924 (Carneiro and El-Deiry, 2020; Peng et al., 2022). MDMX inhibitors, such as XI-011 and DIMP53-1, restore p53 stability by inducing apoptosis and reducing migration in cervical and colon malignancies (Soares et al., 2017; Zhang J. et al., 2022). Small compounds such as PRIMA-1 (APR-017), APR-246 (Eprenetapopt), and COTI-2 restore mutant p53 to a functional state, reactivating its tumor-suppressive capabilities, with potential therapeutic uses in a variety of malignancies (Berke et al., 2022). The p53 agonist HO-3867 restores transcriptional repression in mutant p53, especially in ovarian cancer, resulting in cell death (Devor et al., 2021).

Cyclophilin A (CypA) inhibitors, such as HL001, impede MDM2-mediated p53 degradation, resulting in cell cycle arrest and death in NSCLC (Lu et al., 2017). Natural compounds such as Renieramycin T (RT) (Petsri et al., 2019) and Protopine (Son et al., 2019) stabilize p53, inducing apoptosis in lung and colon tumors, respectively, whereas Andrographolide (ANDRO) degrades mutant p53 (Sato et al., 2018). Actinomycin V and TCCP also increase p53 expression, which causes apoptosis in many cancer cells (Lin S. Q. et al., 2019; Rashmi et al., 2019). Heat-shock protein inhibitors, such as Mortaparib (Plus), reactivate p53 by disrupting its association with mortalin, causing apoptosis in colorectal and breast malignancies (Sari et al., 2021). Furtherrmore, Protoporphyrin IX (PpIX) targets both p53 and its homolog p73, which promotes apoptosis in CLL (Son et al., 2019).

Novel therapeutics include gold complexes like MC3, which upregulate p53 via the ROS formation and have shown effectiveness in colorectal cancer (Dabiri et al., 2019), as well as platinum-based compounds like bromocoumarinplatin 1 and diplatin, which activate p53 to overcome cisplatin or carboplatin resistance respectively in lung cancer (Lin X. et al., 2019; Ma et al., 2020). Other small compounds, such as DJ34, kill leukemia stem cells by inhibiting c-Myc and activating p53 (Tadele et al., 2021), whereas AQ-101 inhibits MDM2 to activate p53 and increase apoptosis in leukemia (Gu et al., 2018).

Research is exploring inhibitors of uncontrolled oncogenic effectors such as PI3K, AKT, β-catenin, Myc, CDKs, mTOR, and VEGF (Sever and Brugge, 2015). CDK4/6 inhibitors like palbociclib enhance cell death and induce cell cycle arrest in various cancers (Tao et al., 2016). Epigenetic strategies focused on inducing apoptosis in cancer cells involve histone deacetylase (HDAC) inhibitors and Bromodomain and Extra-Terminal motif (BET) inhibitors (Bolden et al., 2006; Kim et al., 2018). HDAC inhibitors, such as panobinostat, enhance Noxa expression, reduce Mcl-1 levels, and increase sensitivity to Bcl-2 inhibitors (Liu et al., 2018). They also enhance the effectiveness of MEK inhibitors and venetoclax in treating multiple myeloma (DiNardo et al., 2019). BET inhibitors like ABBV-075, when combined with venetoclax, demonstrate promising outcomes in patients with cutaneous T cell lymphoma (CTCL) (Kim et al., 2018). The hypomethylating agent azacytidine, when combined with venetoclax and ABT-737, has shown promising results (Mishra et al., 2023).

### 1.2 Targeting autophagy for cancer therapy

Autophagy, a critical mechanism for maintaining cellular balance by removing damaged organelles and protein aggregates, can also facilitate cell death (Liu et al., 2023). Cells can undergo autophagy-related cell death in two primary ways: autophagy-dependent cell death (ADCD) which transpires independently of other programmed death mechanisms and autophagy-mediated cell death (AMCD) that occurs when autophagy-related molecules directly engage with those implicated in forms of cell death. (Zhou et al., 2022) (Figure 1). Moreover, autophagy is linked to other cell death mechanisms, such as apoptosis, necrosis, and ferroptosis, through a variety of processes (Dunkle and He, 2011; Gordy and He, 2012; Chen et al., 2018; Liu J. et al., 2020; Peng et al., 2022) (Figure 2).

Three distinct forms of autophagy have been identified: macroautophagy, microautophagy, and chaperone-mediated autophagy (CMA) (Parzych and Klionsky, 2014). Microautophagy is a form of autophagy in which lytic organelles autonomously engulf and degrade cytoplasmic components. It is essential for regulating biosynthesis, transport, metabolic adaptability, organelle remodeling, and the maintenance of cellular component quality (Saha et al., 2018). Macroautophagic autophagosomes convey cellular constituents for destruction to endosomes or lysosomes (Feng et al., 2014). Autophagy starts with an isolating membrane known as the phagophore, which encases a portion of the cytoplasm. The Atg9 protein promotes growth by supplying crucial lipid constituents. The Atg1 and Atg9 proteins, together with a phosphatidylinositol 3-kinase complex, govern this activity. In the subsequent phase, two conjugation steps transpire. The first activation entails Atg12 and the Atg7 protein. The Atg12 protein is conveyed to the Atg10 protein, leading to a covalent connection with Atg5. The Atg12-Atg5 complexes subsequently associate with the Atg16L protein. The ATG12-ATG5-ATG16L1 complex is essential for the production of autophagosomes. The second step of conjugation involves the proteins Atg3, Atg4, Atg7, and LC3. The Atg4 protease cleaves proLC3, resulting in the formation of LC3-I. Subsequently, the Atg7, Atg3, and Atg12-Atg5-Atg16L proteins are conjugated. The LC3-I protein interacts with the lipophilic phosphatidyle-thanolamine (PE) to generate the LC3-II form. These stages generate the autophagosome, which encapsulates a segment of the cytoplasm and proteins. The outer membrane of the autophagosome fuses with the lysosome to form an autophagolysosome. Lysosomal enzymes facilitate the digestion of the autophagolysosome’s inner membrane and its contents (Gomez-Virgilio et al., 2022; Liu et al., 2023). Chaperone-mediated autophagy (CMA) removes damaged proteins during fasting or oxidative stress. The chaperone complex links the protein’s target motif to facilitate lysosome trafficking. In the lysosome, the complex interacts with LAMP-2A’s cytoplasmic tail and is destroyed (Bejarano and Cuervo, 2010).

Autophagy plays a complex role in cancer, acting as both an inhibitor and promoter of tumor growth (Chavez-Dominguez et al., 2020; Nawrocki et al., 2020; Debnath et al., 2023). It can help cancer cells avoid damage induced by chemotherapeutics and promote chemoresistance (Table 1) (Nawrocki et al., 2020; Debnath et al., 2023). Preclinical research using chemotherapeutics like cyclophosphamide, imatinib, and vorinostat has shown that autophagy reduces the effectiveness of these drugs and contributes to acquired resistance (Mele et al., 2020). Furthermore, autophagy aids cancer cells in adapting to chemotherapy (Ahmadi-Dehlaghi et al., 2023).

Autophagy inhibitors have shown promise in combination with chemotherapeutic and targeted immunotherapeutic drugs (Table 3). While many prospective autophagy inhibitors are being developed, chloroquine (CQ) and its derivative hydroxychloroquine (HCQ) are the only approved drugs (Wang et al., 2011). HCQ, like CQ, suppresses autophagy by blocking lysosomal acidification and autophagosome degradation but has lower toxicity (Sui et al., 2013; Cook et al., 2014; Pellegrini et al., 2014; Lee et al., 2015). A Phase II trial for muscle-invasive bladder cancer is investigating the combination of HCQ with gemcitabine and cisplatin for systemic chemotherapy (Ojha et al., 2016). Similarly, in breast cancer, HCQ combined with tamoxifen was found more effective in suppressing autophagy in estrogen-positive (ER+) cell lines (Cook et al., 2014). In renal cell carcinoma, HCQ combined with temsirolimus led to increased apoptosis by inhibiting autophagy (Lee et al., 2015). Early phase I/II trials of HCQ have focused on adult solid tumors, including pancreatic adenocarcinoma, melanoma, colorectal carcinoma, myeloma, lymphoma and renal cell carcinoma, using chemotherapy drugs such as temsirolimus, bortezomib, temozolomide, vorinostat, and doxorubicin (Llovet et al., 2008; Mahalingam et al., 2014; Rangwala et al., 2014a; Rangwala et al., 2014b; Rosenfeld et al., 2014; Vogl et al., 2014; Wolpin et al., 2014). HCQ doses ranged from 400 mg to 600 mg twice daily, showing tolerability with partial responses and stable disease in some patients (Carew and Nawrocki, 2017). For advanced solid tumors and melanoma, HCQ combined with 150 mg/m^2^ of temozolomide showed 27% stable disease and 14% partial response in wildtype melanoma (Cheng et al., 2009; Rangwala et al., 2014b). The combination of HCQ and rapamycin, an inhibitor of mTORC1 activity, was well-tolerated in advanced solid tumor (Rangwala et al., 2014a). In myeloma, HCQ combined with bortezomib improved the efficiency of proteasome inhibitors by causing the accumulation of misfolded proteins, with 45% of patients showing stable disease. The most common adverse effects were gastrointestinal issues and cytopenias (Vogl et al., 2014).

**TABLE 3:** Autophagy targeting drugs for cancer therapy.

Drug/Compound	Mechanism of action	Cancer type	Clinical status	References
Chloroquine (CQ)	Inhibits autophagy by blocking autophagosome-lysosome fusion	Various Solid Tumors, Glioblastoma, Pancreatic Cancer	FDA Approved for Malaria; Clinical Trials in Cancer	Varisli et al. (2020), Agalakova (2024)
Hydroxychloroquine (HCQ)	Inhibits autophagy by preventing lysosomal acidification	Bladder Cancer, Breast Cancer, Renal Cell Carcinoma, Multiple Myeloma, Melanoma	FDA Approved for Malaria and RA; Clinical Trials in Cancer	Sui et al. (2013), Cook et al. (2014), Pellegrini et al. (2014), Lee et al. (2015)
HCQ + Gemcitabine and Cisplatin	HCQ inhibits autophagy, potentially enhancing chemotherapy effectiveness	Muscle-Invasive Bladder Cancer	Phase II Clinical Trials	Ojha et al. (2016)
HCQ + Tamoxifen	HCQ inhibits autophagy, enhancing efficacy of tamoxifen in ER + breast cancer	Breast Cancer (ER+)	Preclinical/Clinical Trials	Cook et al. (2014)
HCQ + Temsirolimus	HCQ inhibits autophagy, increasing apoptosis when combined with mTOR inhibitor	Renal Cell Carcinoma	Phase I Clinical Trials	Lee et al. (2015)
HCQ + Bortezomib	HCQ inhibits autophagy, enhancing proteasome inhibitor efficacy	Multiple Myeloma	Phase I/II Clinical Trials	Vogl et al. (2014)
HCQ + Temozolomide	HCQ inhibits autophagy, enhancing chemotherapy effectiveness	Advanced Solid Tumors and Melanoma	Phase I/II Clinical Trials	Cheng et al. (2009), Rangwala et al. (2014b)
HCQ + Rapamycin	HCQ inhibits autophagy; rapamycin induces autophagy via mTOR inhibition	Advanced Solid Tumors	Phase I Clinical Trials	Rangwala et al. (2014a)
SBI-0206965	ULK1 inhibitor blocking autophagy initiation	Various Cancers	Preclinical	Tang et al. (2017)
Bafilomycin A1	Inhibits vacuolar H (+)-ATPase (V-ATPase), preventing autophagosome-lysosome fusion	Various Cancers	Preclinical	Lu et al. (2015)
Rapamycin (Sirolimus)	Activates autophagy via mTOR inhibition	Renal Cell Carcinoma, Breast Cancer	FDA Approved for Organ Transplant Rejection; Investigational in Cancer	Blagosklonny (2023), Li et al. (2024c)
Everolimus (RAD001)	mTOR inhibitor inducing autophagy	Renal Cell Carcinoma, Breast Cancer, Neuroendocrine Tumors	FDA Approved for RCC, Breast Cancer	Motzer et al. (2008), Singh et al. (2014), Chen et al. (2019)
Temsirolimus (CCI-779)	mTOR inhibitor inducing autophagy	Various Cancers	FDA Approved for RCC	Harshman et al. (2014), Zanardi et al. (2015)
BEZ235 (Dactolisib)	Dual PI3K/mTOR inhibitor inducing autophagy	Breast Cancer, Glioblastoma	Phase I/II Clinical Trials	Wise-Draper et al. (2017), Salazar et al. (2018)
Sunitinib	Induces autophagy via inhibition of multiple tyrosine kinases	Renal Cell Carcinoma, GIST	FDA Approved	Vuorinen et al. (2019), Lara et al. (2024)
Vorinostat (SAHA)	HDAC inhibitor inducing autophagy	Cutaneous T-cell Lymphoma, Solid Tumors	FDA Approved for CTCL; Investigational in other cancers	Mann et al. (2007), Mahalingam et al. (2014)
Arsenic Trioxide (ATO)	Induces autophagy via oxidative stress	Acute Promyelocytic Leukemia, Multiple Myeloma	FDA Approved for Acute promyelocytic leukemia; Investigational in other cancers	Jiang et al. (2023), Gill (2024)
Vitamin D Analogs (e.g., Calcitriol)	Induce autophagy through modulation of AMPK/mTOR pathway	Prostate Cancer, Breast Cancer	Preclinical/Clinical Trials	Duffy et al. (2017), Jeon and Shin (2018)
Metformin	Activates AMPK, inducing autophagy	Breast Cancer, Prostate Cancer, Colorectal Cancer	FDA Approved for Type 2 Diabetes; Investigational in cancer	Kasznicki et al. (2014), Hua et al. (2023)
Chlorpromazine	Antipsychotic drug inhibiting autophagy by interfering with lysosomal function	Glioblastoma, Lung Cancer	Preclinical	Kamgar-Dayhoff and Brelidze (2021), Matteoni et al. (2021)
3-Methyladenine (3-MA)	PI3K inhibitor blocking autophagy initiation	Various Cancers *in vitro*	Preclinical	Zhang et al. (2022e)
Silibinin	Natural compound inducing autophagy via modulation of PI3K/Akt/mTOR pathway	Prostate Cancer, Breast Cancer	Preclinical	Chatran et al. (2018)
Resveratrol	Induces autophagy through SIRT1 activation and mTOR inhibition	Various Cancers	Preclinical/Clinical Trials	Song et al. (2023)
Curcumin	Natural compound inducing autophagy via multiple pathways	Breast Cancer, Colon Cancer	Preclinical/Clinical Trials	Zhao et al. (2016), Saghatelyan et al. (2020), Deng et al. (2024)
Autophinib	Selective ULK1 inhibitor blocking autophagy initiation	Various Cancers *in vitro*	Preclinical	Aleksandrova and Suvorova (2023)
Veru-111 (Sabizabulin)	Microtubule disruptor inducing autophagy and apoptosis	Prostate Cancer, Breast Cancer	Phase II/III Clinical Trials	Krutilina et al. (2022), Markowski et al. (2022)
SBI-0206965	ULK1 inhibitor blocking autophagy initiation	Various Cancers	Preclinical	Tang et al. (2017)
Spautin-1	Beclin-1 inhibitor promoting Vps34 complex degradation	Various Cancers	Preclinical	Kona and Kalivendi (2024)
SAR405	Vps34 kinase inhibitor blocking autophagy initiation	Various Cancers	Preclinical	Pasquier (2015)
Gambogic Acid	Induces caspase-mediated cleavage of autophagy proteins, inhibiting autophagy	Various Cancers	Preclinical	Ishaq et al. (2014)
ATG4 Inhibitors (NSC185058, NSC377071)	Inhibit ATG4B protease, blocking autophagy	Various Cancers	Preclinical	Akin et al. (2014)
Verteporfin	Inhibits early-stage autophagosome formation	Pancreatic Cancer, Glioblastoma	FDA Approved for Macular Degeneration; Investigational in Preclinical Cancer Studies	Donohue et al. (2011)
Lysosomal Inhibitors (ROC325, Lys05, DQ661, DC661)	Inhibit lysosomal function, blocking autophagic flux	Various Cancers	Preclinical	Amaravadi and Winkler (2012), Rebecca et al. (2017), Nawrocki et al. (2019), Rebecca et al. (2019)
Z-DEVD (Caspase-3 Inhibitor) + RAD001 + Irradiation	Induce autophagy by inhibiting apoptosis and mTOR, enhancing radiation-induced autophagy	Non-Small Cell Lung Cancer	Preclinical	Kim et al. (2008)
Silver Nanoparticles (AgNPs)	Induce autophagy; effect confirmed with autophagy inhibitor 3-MA	Glioma	Preclinical	Wu et al. (2015)

Autophagy activation by drugs like sorafenib, a multi-tyrosine kinase mTOR inhibitor used for hepatocellular cancer, is being explored as a potential cause of drug resistance (Llovet et al., 2008). Clinical trials have investigated HCQ in hepatocellular cancer (Cheng et al., 2009) and targeted immunotherapeutic treatments, such as checkpoint inhibitors, have limited efficacy and high costs. Combining autophagy modulators like HCQ with immune checkpoint inhibitors (ICI) has the potential to improve efficacy and reduce treatment costs. Several inhibitors targeting different stages of autophagy are under investigation including inhibitors of upstream signaling molecules: SBI-0206965 (ULK1 inhibitor) (Tang et al., 2017), Spautin-1 (Beclin1 inhibitor), SAR405 (Vps18 kinase inhibitor), and gambogic acid (induces caspase-mediated cleavage of autophagy proteins) (Ishaq et al., 2014; Pasquier, 2015). Autophagy initiation inhibitors include ATG4 inhibitors NSC185058 and NSC377071, and Verteporfin (inhibits early-stage autophagosome formation) (Donohue et al., 2011; Akin et al., 2014). Lysosomal inhibitors include ROC325 (Nawrocki et al., 2019), Lys05 (Amaravadi and Winkler, 2012), DQ661 (Rebecca et al., 2017), and DC661 (Rebecca et al., 2019).

Preclinical trials have also explored autophagy’s potential to enhance radiation therapy (Kim et al., 2008; Kuwahara et al., 2011). For example, in a lung cancer mouse model, combining Z-DEVD (caspase-3 inhibitor), RAD001 (mTOR inhibitor), and irradiation induced the highest levels of autophagy and associated radiation damage. This suggests that inhibiting both apoptosis and mTOR during radiotherapy could improve outcomes in non-small cell lung cancer patients (Kim et al., 2008). Similarly, in glioma cells, autophagy induction by silver nanoparticles (AgNPs) and/or radiation was confirmed by applying 3-methyladenine (3-MA), highlighting selective autophagy as a promising therapeutic avenue for effective cancer treatment (Wu et al., 2015).

### 1.3 Necroptosis in cancer development and treatment

Programmed inflammatory cell death, known as necroptosis, was first identified as an alternative to apoptosis following the activation of death domain receptors (Degterev et al., 2005; Dhuriya and Sharma, 2018) (Figure 1). Necroptosis is a regulated type of necrosis that is dependent on receptor interacting kinase-1 (RIPK1) and RIPK3 phosphorylating mixed-lineage kinase-like (MLKL) (Vandenabeele et al., 2010; Sun et al., 2012; Newton et al., 2014) (Figure 2). The necroptotic process begins when RNA- and DNA-sensing molecules and cell surface death receptors including FasRs, TNFR1, IFN receptors, and TLRs are activated (Kaiser et al., 2013). There are two ways that cell death signaling continues (Pasparakis and Vandenabeele, 2015). Complex I, a survival complex that communicates via NF-kB, can be created by TNF-α. RIPK1 deubiquitination transforms the complex into apoptotic complex IIa. When caspase-8 is absent and RIPK3 is elevated, the complex forms IIb (the necrosome). The death domain-related proteins RPK1, RPK3, and Fas on this necrosome directly phosphorylate the kinase domain-like protein (MLKL) to induce necroptosis. MLKL phosphorylation forms an oligomer that punctures the plasma membrane, killing the cell. Calmodulin-dependent protein kinase and mitochondrial serine/threonine protein phosphatase II are other RIPK3 downstream effects (He et al., 2009; Cai et al., 2014; Wang et al., 2014; Murphy, 2020). Necroptotic cell death is characterized by cell membrane perforation, elevated intracellular osmotic pressure, cell rounding and swelling, organelle swelling, impaired mitochondrial activity, mitochondrial membrane potential loss, nuclear chromatin loss, and plasma membrane rupture (Dhuriya and Sharma, 2018). Plasma membrane rupture causes potassium efflux, cytokines, and chemokines, which cause inflammation and immunological responses (Dhuriya and Sharma, 2018).

Necroptosis is involved in various aspects of tumor biology, including tumor development, necrosis, metastasis and the immune response within tumors (Najafov et al., 2017; Gong et al., 2019; Yan et al., 2022; Meier et al., 2024). This cell death pathway exhibits both pro- and anti-tumorigenic effects (Ye et al., 2023). Major regulators of necroptosis are often downregulated in cancer cells, correlating with unfavorable outcomes (Table1) (Yan et al., 2022). Necroptosis has emerged as a novel target for anticancer therapy due to its significant role in tumor biology (Gong et al., 2019).

Several natural compounds and small molecule inhibitors are known to induce necroptosis in cancer cells (listed in Table 4) (Wu et al., 2020). Chloroquine increases the expression of endogenous RIPK3 in colorectal cancer cell lines, with necroptosis being the mechanism (Meng et al., 2016). Shikonin, derived from a Chinese medicinal herb, induces necroptosis in nasopharyngeal carcinoma cells by enhancing reactive oxygen species (ROS) production and increasing RIPK1, RIPK3 and MLKL expression (Liu et al., 2019). Emodin triggers necroptosis in glioma cell lines by activating the TNF/RIPK1/RIPK3 pathway (Zhou et al., 2020). Neoalbaconol (NA), a compound derived from the fungus Albatrellus confluens, has been found to trigger necroptosis by facilitating the autocrine release of TNFα through the modulation of the RIPK/NF-κB signaling pathway and RIPK3-dependent reactive oxygen species (ROS) generation (Yu et al., 2015). The steroid glycoside Ophiopogonin D induces necroptosis in prostate cancer cells by activating RIPK1 (Lu et al., 2020). Resibufogenin inhibits colorectal cancer cell line growth by increasing RIPK3 expression (Han et al., 2018). The initiation of necroptosis can also be influenced by adjusting upstream signaling pathways, such as using the sphingosine analog FTY720 (fingolimod), which triggers necroptosis in human lung cancer cells by interacting with the I2PP2A/SET oncoprotein and activating the PP2A/RIPK1 pathway (Saddoughi et al., 2013).

**TABLE 4:** Necroptosis targeting drugs for cancer therapy.

Drug/Compound	Mechanism of action	Cancer type	Clinical status	References
Chloroquine	Increases expression of RIPK3	Colorectal Cancer	FDA Approved for Malaria; Investigational in cancer	Meng et al. (2016)
Shikonin	ROS production and upregulation of RIPK1, RIPK3, MLKL	Nasopharyngeal Carcinoma, Various Cancers	Preclinical	Han et al. (2007), Boulos et al. (2019), Liu et al. (2019)
Emodin	activation of TNFα/RIPK1/RIPK3 pathway	Glioma, Various Cancers	Preclinical	Zhou et al. (2020), Sharifi-Rad et al. (2022)
Neoalbaconol (NA)	modulation of RIPK/NF-κB pathway and RIPK3-dependent ROS generation	Breast Cancer	Preclinical	Yu et al. (2015)
Ophiopogonin D	activates RIPK1	Prostate Cancer	Preclinical	Lu et al. (2020)
Resibufogenin	increasing RIPK3 expression,	Colorectal Cancer	Preclinical	Han et al. (2018)
FTY720 (Fingolimod)	I2PP2A/SET oncoprotein interaction and activation of PP2A/RIPK1 pathway	Lung Cancer	FDA Approved for Multiple Sclerosis; Investigational in cancer	Saddoughi et al. (2013)
Fe(III)-Shikonin Supramolecular Nanomedicine (FSSN)	Induces necroptosis and ferroptosis; improved delivery and reduced toxicity	Colon Cancer	Preclinical	Feng et al. (2022)
Graphene Oxide Nanoparticles	enhances RIPK1, RIPK3, and HMGB1 activity	Colon Cancer	Preclinical	Chen et al. (2015)
Selenium Nanoparticles	increased ROS production and upregulation of TNFα and IRF1	Prostate Cancer	Preclinical	Sonkusre (2019)
Myricetin-loaded Solid Lipid Nanoparticles (MYC-SLNs)	increases RIPK3 and MLKL expression	Lung Cancer (A549 cells)	Preclinical	Alidadi et al. (2022)
Dimethyl Fumarate (Delivered via sPCPEG-azo Micelles)	depletes GSH, increasing ROS levels, activating MAPKs	Colon Cancer	Preclinical	Ma et al. (2016c)
Necrosulfonamide (NSA)	MLKL inhibitor;	Various Cancers	Preclinical	Liu et al. (2016)
Necrostatin-1	RIPK1 inhibitor	Colorectal Cancer	Preclinical	Liu et al. (2015), Cao and Mu (2021)
GSK2982772	RIPK1 inhibitor	Approved for Inflammatory Diseases; investigational in Cancer	Phase IIa Clinical Trials (Inflammatory Diseases)	Wu et al. (2020), Tong et al. (2022)
Trichothecin	increases RIPK3 expression, enhancing MLKL phosphorylation, activating ROS production	Chemoresistant Cancers	Preclinical	Zhao et al. (2021b)
CBL0137	Induces Z-type dsDNA formation, activating ZBP1-dependent necroptosis; reverses insensitivity to immune checkpoint inhibitors	Melanoma	Preclinical	Zhang et al. (2022c)

Nanoparticles to induce necroptosis in cancer cells is another emerging field (Mohammadinejad et al., 2019). Although the antifungal agent Shikonin shows potential, its clinical use is limited due to poor tumor specificity, low water solubility, short bloodstream half-life, and high risk of side effects on healthy tissues (Boulos et al., 2019). To address these issues, Feng et al. developed an Fe(III)-shikonin supramolecular nanomedicine (FSSN) using metal-polyphenol coordination of Fe(III) and shikonin, demonstrating improved water solubility and reduced cytotoxicity in normal cells and induced both ferroptosis and necroptosis (Feng et al., 2022). In CT26 colon cancer cells, graphene oxide nanoparticles triggered necroptosis by enhancing RIPK1, RIPK3, and HMGB1 activity (Chen et al., 2015). Similarly, selenium nanoparticles induced necroptosis in prostate adenocarcinoma cells by increasing ROS production and TNF and interferon regulatory factor 1 expression (Sonkusre, 2019). Folate-sodium alginate-cholesterol nanoparticles delivering doxorubicin and metformin achieved targeted accumulation and induced various forms of programmed cell death, including necroptosis, apoptosis, and pyroptosis in xenograft melanoma tumors (Song et al., 2021). Myricetin-loaded solid lipid nanoparticles (MYC-SLNs) enhanced necroptosis in A549 cells by increasing RIPK3 and MLKL expression without affecting apoptosis and without apparent effects on the growth and health of MRC5 cells (Alidadi et al., 2022). Ma et al. developed star-PCL-azo-PEG micelles (sPCPEG-azo) to deliver dimethyl fumarate (DMF) specifically to the colon, inducing necroptosis by eliminating GSH, increasing ROS levels and activating MAPKs (Ma Z. G. et al., 2016).

In a study conducted by Liu et al., MLKL inhibitor necrosulfonamide (NSA) was shown to significantly delay tumor growth, thus offering compelling evidence of the role necroptosis plays in promoting tumor development (Liu et al., 2016). In mice, the use of necrostatin-1, another necroptosis inhibitor, has been found to be effective in reducing colitis-associated tumorigenesis (Liu et al., 2015). There is ongoing testing of the RIPK1 inhibitor, GSK2982772, in phase 2a clinical studies for individuals with inflammatory disease. Furthermore, in a clinical trial (NCT04739618), researchers explored the potential benefits of nonablative cryosurgical freezing-induced necroptosis followed by immunotherapeutic drug injection in metastatic solid tumors. The immunotherapy included pembrolizumab (anti-PD1), ipilimumab (anti-CTLA-4), and GM-CSF. The aim was to assess the overall response rate of radiographic changes (Tong et al., 2022). In addition, induction of apoptosis has also been shown to reverse drug resistance. Xu Zhao et al., effectively employed trichothecin to trigger necroptosis in cancers that are resistant to chemotherapy. Mechanistically, the natural secondary metabolite trichothecin significantly increased the expression of RIPK3. Subsequently, RIPK3 enhanced the phosphorylation of MLKL and activated mitochondrial energy metabolism and ROS production. This novel approach sensitizes cancer cells to cisplatin therapy (Zhao X. et al., 2021).

In addition, it has been shown that necroptosis-inducing drugs could impact the effectiveness of ICIs in individuals with cancer (Tang et al., 2020). Using a viral vaccination strategy, Hoecke et al. were able to effectively deliver the necroptosis mediator MLKL to tumor cells, resulting in the promotion of necroptotic death and the enhancement of antitumor immunity. Increased immunity directly against neo-epitopes was responsible for the potent antitumor immunity (Van Hoecke et al., 2020). In addition, the RNA editing enzyme ADAR1 has been widely recognized for its role in suppressing Z-type dsRNA, a substrate for ZBP1. This suppression mechanism leads to resistance and limited responsiveness to ICIs (Zhang T. et al., 2022). However, the small-molecule drug CBL0137 has the ability to directly induce the formation of Z-type dsDNA in cells. This in turn activates ZBP1-dependent necroptosis and effectively reverses the insensitivity to ICIs in mouse melanoma models (Zhang T. et al., 2022). In addition, cIAPs have the ability to hinder the RIPK1-dependent necroptosis process. However, this inhibition can be counteracted by Smac mimetics which then trigger the activation of the necroptotic death pathway in Burkitt’s lymphoma cell lines (Koch et al., 2021). In melanoma, the response to ICIs can be enhanced by using Smac mimetics which have a direct impact on immune cells such as B cells, MDSCs, DCs, and cytotoxic T cells (Michie et al., 2020). Based on the evidence, it appears that necroptosis could potentially be employed to enhance the readiness of the tumor microenvironment for immunotherapy.

Even with progress in necroptosis research, various obstacles impede its application in cancer treatment. The practicality of necroptosis, having potential as an alternative therapy for tumors resistant to apoptosis, continues to be debated. Hitomi et al. (2008) identified a cellular signaling network that regulated necroptosis and implicated two suppressor genes, CYLD and EDD1, and four Ras-related proteins, suggesting a role in tumorigenesis. CYLD gene mutations in tumorigenic epidermal cells promote carcinoma aggressiveness by increasing angiogenic factor production, which is crucial to epidermal cancer malignancy (Alameda et al., 2010). RIPK3 and CYLD were downregulated in CLL cells, and LEF1 represses CYLD. Together, necroptosis may be crucial to carcinogenesis (Reed, 2006). Tumor heterogeneity presents a significant challenge, as numerous cancers are deficient or have mutated for essential necroptosis regulators such as RIPK3 or MLKL, which restricts the effectiveness of necroptosis inducers. RIPK3-r, a truncated splice variation of RIPK3, was dramatically elevated in colon and lung tumors compared to matched normal tissues, suggesting that it may be a primary splice form involved in carcinogenesis, according to Yang et al. (2005). The RIPK3 gene lies on chromosome 14q11.2, which is mutated in several malignancies, including nasopharyngeal carcinoma and T cell leukemia/lymphoma (Kasof et al., 2000). In non-Hodgkin lymphoma, RIPK3 gene polymorphisms increases tumor risk (Wu et al., 2012). Furthermore, existing inducers exhibit a lack of selectivity, resulting in uncontrolled inflammation and the possibility of harming healthy tissues, which raises concerns regarding off-target effects and systemic toxicity. Necroptosis may enhance anti-tumor immunity due to its pro-inflammatory characteristics, yet it also has the potential to facilitate tumor progression by creating a pro-tumor environment. Necroptosis of tumor cells can affect the TME in a way that can contribute to tumor growth because the inflammation associated with necroptosis can stimulate cell division, genetic instability, angiogenesis and metastasis (Negroni et al., 2020). Furthermore, the restricted clinical evidence, primarily based on preclinical models, along with the unpredictable nature of necroptosis outcomes, adds to the complexity of its application. Tumors can develop resistance to necroptosis, similar to how they respond to therapies that induce apoptosis. Consequently, additional research is essential to enhance targeting specificity, reduce inflammatory risks, and confirm the efficacy of necroptosis-based treatments in clinical environments.

### 1.4 The roles of pyroptosis in cancer cell survival and treatment strategies

Pyroptosis is a form of RCD associated with inflammatory responses. It is triggered by human caspase-1, -3, -4, -5 (mouse caspase-11), −6, −8, and −9 and NLRP3 and has significant therapeutic implications for several malignancies due to its profound effects on the invasion, proliferation, and metastasis of tumor cells (Table 1) (Shi J. et al., 2014; Fang et al., 2020; Zheng and Li, 2020; Rao et al., 2022; Wei et al., 2022) (Figure 1). Gasdermin (GSDM) superfamily members, GSDMA-GSDME, which are essential to pyroptosis, are triggered by caspases and perforate the plasma membrane (Kayagaki et al., 2015; Shi et al., 2015; Rogers et al., 2019) (Figure 2). The characteristic features of pyroptosis in cancer involve gasdermins family protein cleavage and polymerization, both N-terminal and C-terminal junction domain cleavage, and activated N-terminal regions. The N-terminal generates a cell membrane pore by binding to membrane lipids, phosphatidylinositol, and cardiolipin, causing cell osmotic swelling, plasma membrane rupture, and death (Ding et al., 2016; Feng et al., 2018). Gasdermins create 10–20 nm holes in cell membranes, releasing cell contents slowly and potentially causing inflammatory responses. The cells become flattened eventually and create 1–5 μm apoptotic body-like protrusions. Nuclear concentration and chromatin DNA breaking occur as cells enlarge to rupture plasma membrane (Zhang et al., 2018). Pyroptotic pathway can occur either through classical or non-classical pathway in cancer. The classical pathway is activated by pathogen-associated molecular patterns (PAMPs) or damage-associated molecular patterns (DAMPs) (Chen and Nunez, 2010; Franchi et al., 2012). Cytoplasmic pattern recognition receptors (PRRs) identify them. Based on particular inputs, nod-like receptors (NLRs) or melanoma deficiency factor 2-like receptors (ALRs) produce inflammatory bodies and activate caspase-1. After Caspase-1 cleaves GSDMD, its N-terminal aggregates into cell membrane holes (Amarante-Mendes et al., 2018; Zheng D. et al., 2020) (Figure 2). Additionally, caspase-1 cleaves pro-IL-1β and pro-IL-18 into mature IL-1β and IL-18, which are then released via the membrane hole. The nonc-lassical pyrolytic pathway requires caspase-4/-5/-11 activation. After lipopolysaccharide (LPS) stimulates the cytoplasm, caspase-4/caspase-5/caspase-11 (the human equivalent of mouse caspase-11 caspase-4/caspase-5) can directly bind to the conserved structure of LPS, lipoprotein A, causing oligomerization, activation, and the N-terminal of GSDMD to be cleaved and localized to the cell membrane to form membrane pores (Kayagaki et al., 2015). Pyroptosis is quicker and more violent than apoptosis, releasing several pro-inflammatory molecules. Cell scorch is caused by inflammatory corpuscles and GSDM family proteins.

Several chemotherapeutic drugs, including cisplatin, paclitaxel, 5-FU, lobaplatin and others have been found to trigger pyroptosis in tumor cells (Table 5) (Zhang C. C. et al., 2019; Jia et al., 2023). Chemotherapy-induced pyroptosis is frequently the result of GSDME pathway activation. Chemotherapy drug Lobaplatin triggers pyroptosis in cervical cancer and colorectal cancer by activating GSDME (Chen et al., 2022). This effect is achieved by activating caspase-3/9 through the ROS/JNK/BAX mitochondrial apoptosis pathway (Yu et al., 2019). 5-FU triggers pyroptosis in gastric cancer cells via GSDME instead of GSDMD (Wang Y. et al., 2018). When exposed to cisplatin or 5-FU, GSDME^+/+^ mice experience significant intestinal damage and infiltration of immune cells. On the other hand, GSDME^−/−^ mice show less injury, indicating that triggering pyroptosis in cancer cells might offer a potential alternative approach for cancer treatment (Yu and He, 2017).

**TABLE 5:** Pyroptosis targeting drugs for cancer therapy.

Drug/Compound	Mechanism of action	Cancer type	Clinical status	References
Cisplatin (DDP)	Induces pyroptosis via activation of GSDME through caspase-3	Various Cancers (e.g., gastric, esophageal)	FDA Approved Chemotherapy Agent	Yan et al. (2021), Xuzhang et al. (2024), Wang et al. (2018b) ^1^, Wu et al. (2019a) ^2^
5-Fluorouracil (5-FU)	Triggers pyroptosis via GSDME activation	Gastric Cancer, Colorectal Cancer	FDA Approved Chemotherapy Agent	Yu and He (2017), Wang et al. (2018b)
Paclitaxel	Induces pyroptosis through GSDME activation	Various Solid Tumors	FDA Approved Chemotherapy Agent	Zhang et al. (2019a)
Lobaplatin	Induces pyroptosis via ROS/JNK/BAX pathway activating caspase-3/9 and GSDME	Cervical Cancer, Colorectal Cancer	Approved in China; Investigational elsewhere	Yu et al. (2019), Chen et al. (2022)
Atezolizumab	Induces pyroptosis when combined with chemotherapy/radiation	Non-Small Cell Lung Cancer	FDA Approved	Wang et al. (2022d)
Trimethylamine N-oxide (TMAO)	Triggers GSDME-mediated pyroptosis; enhances anti-PD-1 effects	Breast Cancer	Preclinical	Wang et al. (2022a), Jia et al. (2023)
Val-boroPro (Talabostat)	Activates CARD8 inflammasome, leading to caspase-1 activation and pyroptosis	Acute Myeloid Leukemia (AML)	Clinical trials halted after Phase II	Johnson et al. (2018)
BRAF Inhibitors (BRAFi) and MEK Inhibitors (MEKi)	Induce pyroptosis via GSDME activation when combined	Melanoma	FDA Approved (both agents separately); Combination approved	Erkes et al. (2020)
BI2536 (PLK1 Inhibitor) with Cisplatin	Combination induces pyroptosis via caspase-3/GSDME pathway	Esophageal Cancer	Preclinical	Wu et al. (2019a)
Ivermectin	Activates pannexin-1 pathway, leading to pyroptosis via P2X4/P2X7 receptors	Triple-Negative Breast Cancer	FDA Approved for Parasitic Infections; Investigational in cancer	Draganov et al. (2015)
Metformin	Triggers GSDMD-mediated pyroptosis via AMPK/NLRP3 pathway	Various Cancers	FDA Approved for Type 2 Diabetes; Investigational in cancer	Pizato et al. (2018), Zheng et al. (2020c)
Anthocyanin	Induces GSDMD-mediated pyroptosis	Breast Cancer	Dietary Supplement (Preclinical in cancer)	Yue et al. (2019)
Docosahexaenoic Acid (DHA)	Triggers pyroptosis via GSDMD activation	Colon Cancer	Dietary Supplement (Preclinical in cancer)	Wang et al. (2019a), Wang et al. (2019a) ^18^

Clinical trials have demonstrated that the combination of PD-L1 inhibitors with chemotherapy or radiation can effectively eliminate tumor cells through pyroptosis induction (Reck et al., 2019). This approach has shown promising results in terms of improved patient survival rates, surpassing those observed in patients solely treated with PD-L1 inhibitors. In breast cancer cells, the presence of Trimethylamine N-oxide (TMAO) can trigger GSDME-mediated pyroptosis (Wang H. et al., 2022), and when TMAO is combined with PD-1, it has the potential to enhance the antitumor effects of anti-PD-1 (Jia et al., 2023).

CAR-T cells have been successfully utilized to effectively treat hematological malignancies, yielding favorable outcomes (Gill and Brudno, 2021). However, cytokine release syndrome (CRS) is a significant side effect of this technology. When CAR-T cells release granzyme B, it can trigger pyroptosis by activating the caspase-3/GSDME pathway (Liu Y. et al., 2020). Interestingly, the elimination of GSDME through knockout has been found to effectively prevent CRS. Furthermore, the presence of perforin/granzyme B in CAR-T cells, as opposed to in CD8^+^ T cells, triggers GSDME-mediated pyroptosis in target cells (Liu Y. et al., 2020). These findings underscore the clinical importance of pyroptosis in immunotherapy. The release of IL-1β and IL-18 by pyroptotic cells, along with other DAMPs, can attract immune cells like dendritic cells (DCs) and macrophages (MFs) to engulf the pyroptotic cells (Wang Q. et al., 2018; Karki and Kanneganti, 2021). Mature DCs display tumor-specific antigens to activate cytotoxic T lymphocytes, which then eliminate tumors (Wang et al., 2013).

Targeted drugs have also been discovered that selectively trigger pyroptosis in tumor cells (Table 5). Val-boroPro triggers pyroptosis in primary acute myeloid leukemia (AML) cells by activating the inflammasome sensor protein CARD8 which then activates procaspase-1 (Johnson et al., 2018). In a melanoma study, it was found that the combination of BRAFi and MEKi could potentially have an antitumor effect by inducing pyroptosis through GSDME (Erkes et al., 2020). Additionally, the combination of DDP and BI2536 (a PLK1 kinase inhibitor) was observed to induce pyroptosis in esophageal cancer cells (Wu M. et al., 2019). In a study conducted by Dobrin et al., triple-negative breast cancer cells were exposed to ivermectin, resulting in the activation of the pannexin-1 pathway. This activation led to the overexpression of P2X4/P2X7 receptors, the release of ATP and ultimately the induction of pyroptosis (Draganov et al., 2015). Several drugs, including metformin, anthocyanin, and DHA have been found to trigger GSDMD mediated pyroptosis in different types of cancers (Pizato et al., 2018; Wang L. et al., 2019; Yue et al., 2019).

### 1.5 Clinical development targeting ferroptosis for cancer treatment

Ferroptosis is a recently discovered RCD pathway distinguished by oxidative and non-apoptotic mechanisms. It is characterized by iron-dependent lipid peroxide damage in mitochondria and a lack of glutathione peroxidase 4 (GPX4) and is distinct from apoptosis, autophagy, and necrosis (Dixon et al., 2012) (Figures 1, 2). From a morphological perspective, the cell membrane stays intact while developing blisters. The mitochondria decrease in size, and their membrane density increases. The mitochondrial cristae may either reduce in number or vanish entirely. The nucleus retains its typical size, while the chromatin remains uncondensed. Ferroptosis takes place when there is a disruption in the regulatory system, resulting in the buildup of lipid peroxide to a critical level. Transferrin (TF) attaches to extracellular Fe3+ and aids in its transport into cells through transferrin receptor 1 (TfR1), where it is subsequently transformed into Fe2+ (Masaldan et al., 2018). Subsequently, intracellular divalent metal transporter 1 (DMT1) and zinc transporter 8/14 (ZIP8/14) facilitate the storage of Fe2+ in the intracellular labile iron pool (LIP) (Sterling et al., 2017). Fe2+ can transfer electrons via the Fenton reaction with peroxide, leading to the generation of oxidizing free radicals. After an excessive buildup of iron within cells, many free radicals engage with polyunsaturated fatty acids (PUFA) present in the phospholipids of cell membranes, leading to the creation of lipid peroxides, which ultimately result in cell death (Doll et al., 2017). The intracellular antioxidant stress system relies on GPX4 to remove excess lipid peroxides. The Cystine/glutamate antiport (system xc−) enables the transfer of glutamate from inside cells to the exterior, while concurrently bringing cystine from outside into the cells. Inhibiting cysteine with system xc− blockers like erastin reduces the necessary cysteine levels for GSH production and disrupts GSH synthesis. GPX4 facilitates the hydrolysis of lipid peroxide through the action of GSH. Enhancing ferroptosis requires the inhibition of system Xc−, depletion of GSH, and deactivation of GPX4 (Yang et al., 2014).

Cancer cells’ higher iron (Fe) accumulation makes them more susceptible to ferroptotic cell death, thereby impacting tumor development, proliferation and metastasis (Table 1) (Maru et al., 2022; Lei et al., 2024; Zhou Q. et al., 2024). Several ferroptosis inducers have been developed, and their effectiveness varies in different cancer types (listed in Table 6) (Luo et al., 2024; Zhou Q. et al., 2024). Sorafenib, an FDA-approved chemotherapeutic for hepatocellular carcinoma (HCC), renal cell carcinoma (RCC), and thyroid cancer stimulates ferroptosis by inhibiting system XC− and glutathione (GSH) formation (Dixon et al., 2014; Sun et al., 2017). Combining sorafenib with sulfasalazine can further inhibit sulfur-based amino acid metabolism, triggering ferroptosis in HCC cells both *in vitro* and *in vivo* (Wang K. et al., 2021). In NSCLC and colon cancer, cisplatin induces ferroptosis by depleting GSH and inactivating GPX4 (Guo et al., 2018). Etoposide, a phenolic anticancer drug, depletes GSH in myeloperoxidase-rich myelogenous leukemia cells, reducing GPX4 and triggering ferroptosis (Kagan et al., 2017). Further, Ma et al. demonstrated that the combination of the lysosome disruptor siramesine with the tyrosine kinase inhibitor lapatinib resulted in the ferroptotic death of breast cancer cells. This was achieved by blocking iron transport and inducing lipid peroxidation (Ma S. et al., 2016). The combination of DHA with cisplatin triggered cell death in pancreatic ductal adenocarcinoma (PDAC) by promoting the degradation of GPX4, creation of ROS and the degradation of ferritin, leading to induction of ferroptosis (Du J. et al., 2021).

**TABLE 6:** Ferroptosis targeting drugs for cancer therapy.

Drug/Compound	Mechanism of action	Cancer type	Clinical status	References
Erastin	Inhibits system Xc− depleting GSH	Various Cancers	Preclinical	Bakar-Ates and Ozkan (2024), Sinha et al. (2024), Wu et al. (2024a)
RSL3	Directly inhibits GPX4, leading to lipid peroxidation	Various Cancers (Research tool)	Preclinical	Dixon et al. (2014), Sun et al. (2017)
Sorafenib	Inhibits system Xc−; multi-kinase inhibitor	Hepatocellular Carcinoma, Renal Cell Carcinoma	FDA Approved for HCC, RCC; Investigational for ferroptosis	Dixon et al. (2014), Sun et al. (2017)
Sulfasalazine	Inhibits system Xc−, depleting GSH and inducing ferroptosis	Glioma, Breast Cancer, Hepatocellular carcinoma	FDA Approved for RA and UC; Investigational in cancer	Wang et al. (2021b)
Artesunate	Iron-dependent ROS generator	Pancreatic Cancer, Breast Cancer, lung cancer	FDA Approved for Malaria; Investigational in cancer	(Eling et al., 2015, Wang et al., 2021b, Wang et al., 2024a)
Dihydroartemisinin	GPX4 inhibitor	pancreatic ductal adenocarcinoma	Preclinical studies	Du et al. (2021a)
Lycorine	GPX4 inhibitor	Renal cell carcinoma	Preclinical studies	Du et al. (2021b)
Osimertinib	EGFR inhibitor	Non-small cell lung cancer (NSCLC)	FDA-approved	Zhang et al. (2023)
Lapatinib	inhibits system Xc− and HER2/EGFR pathways	Breast Cancer	FDA Approved for HER2+ Breast Cancer	Ma et al. (2016b)
Altretamine	Induces lipid peroxidation leading to ferroptosis	Ovarian Cancer, Head and neck cancer	FDA Approved; Investigational for ferroptosis	Keldsen et al. (2003), Roh et al. (2016)
FIN56	Promotes degradation of GPX4 and depletes coenzyme Q10	Bladder cancer	Preclinical	Shimada et al. (2016)
FINO2	Oxidizes iron and directly oxidizes lipids, inducing ferroptosis	Various Cancers	Preclinical	Gaschler et al. (2018)
Dihydroartemisinin (DHA)	ROS generation and iron metabolism alteration	Hepatocellular Carcinoma, Leukemia	Preclinical/Clinical Trials	Yuan et al. (2020), Du et al. (2021a), Li et al. (2024b)
Carboplatin	Enhances ferroptosis via iron accumulation and ROS generation	Ovarian Cancer	FDA Approved Chemotherapy Agent	Basuli et al. (2017)
Cisplatin	under certain conditions promotes lipid peroxidation	Lung Cancer, Ovarian Cancer	FDA Approved Chemotherapy Agent	Guo et al. (2018)
Etoposide	Depletes GSH	leukemia	Preclinical	Kagan et al. (2017)
Imidazole Ketone Erastin (IKE)	More potent analog of erastin; inhibits system Xc−	Various Cancers	Preclinical	Zhang et al. (2019c)
ML162 and ML210	Potent GPX4 inhibitors	Various Cancers	Preclinical	Viswanathan et al. (2017)
Statins (e.g., Simvastatin)	Decrease coenzyme Q10 synthesis	Prostate Cancer, Breast Cancer	FDA Approved for Hypercholesterolemia; Investigational in cancer	Shimada et al. (2016), Yao et al. (2021)
SRS16-86	GPX4 inhibitor	Pancreatic Cancer	Preclinical	Pu et al. (2022)
Siramesine	Lysosomal disruptor that induces ferroptosis in combination with lapatinib	Breast Cancer	Preclinical	Ma et al. (2016b)
Vitamin C (High Dose)	Induces ferroptosis via depletion of GSH and iron-dependent ROS generation	Colorectal Cancer, Pancreatic Cancer	Clinical Trials	Yun et al. (2015), Wang et al. (2021d)
β-Elemene	Induces ferroptosis through regulation of iron metabolism	Glioblastoma, Lung Cancer	Preclinical	Tan et al. (2021)
FePt@MoS2	release Fe(II) in the TME, accelerating the Fenton reaction	Solid tumors	Preclinical	Zhang et al. (2019b)
Zero-valent iron nanoparticles transformed Fe(II	to enhance the Fenton reaction	Oral cancer cells	Preclinical	Huang et al. (2019a)
FSSN	Reduces GSH levels	Breast cancer	Preclinical	Feng et al. (2022)

Nanotechnology enhances RCD induction by delivering inducers directly to tumors (Mohammadinejad et al., 2019). FePt@MoS2 nanoparticles, for example, release Fe(II) in the tumor microenvironment, accelerating the Fenton reaction and triggering ferroptosis in various cancer cell lines (Zhang D. et al., 2019). Similarly, additional research showed that zero-valent iron nanoparticles transformed Fe(II) to enhance the Fenton reaction, resulting in mitochondrial lipid peroxidation in oral cancer cells (Huang K. J. et al., 2019). In addition, FSSN based on the metal-polyphenol coordination of Fe(III) and shikonin, led to necroptosis and a reduced GSH level induced ferroptosis in mouse breast cancer cell lines (Feng et al., 2022).

Radiation therapy induces ferroptotic cell death by generating reactive oxygen species (ROS), leading to lipid peroxidation (Lang et al., 2019; Lei et al., 2020; Ye et al., 2020). ROS extract electrons from polyunsaturated fatty acids (PUFAs), forming lipid peroxyl radicals and hydroperoxides. Radiation also upregulates ACSL4 to facilitate PUFA-phospholipid production and also reduces GSH levels, impairing GPX4 and promoting ferroptosis (Lei et al., 2020; Ye et al., 2020; Zhang C. et al., 2022). Moreover, studies have demonstrated that disulfiram induces lysosomal membrane permeabilization through a process dependent on reactive oxygen species (ROS), leading to ferroptosis induction and enhancing the vulnerability of cells to radiation (Ye L. et al., 2021).

ICIs have advanced cancer therapy, but their efficacy is limited without tumor-associated antigens (Ding et al., 2022; Kou et al., 2023). CD8^+^ T lymphocytes can suppress tumors by triggering necroptosis, pyroptosis, and ferroptosis (Tang et al., 2020; Chen L. et al., 2021; Liao P. et al., 2022; Wang Z. et al., 2022). Unique RCDs in the TME stimulate proinflammatory cytokines and cytotoxic T cell infiltration, enhancing ICI responsiveness (Workenhe et al., 2020). Lipid peroxides generated during ferroptosis signal DCs to present tumor antigens to CD8^+^ T cells, improving immunotherapy (Zhao et al., 2022). Combining ferroptosis inducers with ICIs may enhance cancer cell susceptibility to immunotherapy. Wang and colleagues have demonstrated that the concurrent administration of a GPX4 inhibitor, cyst(e)inase, and PD-L1 inhibition enhances T cell-mediated antitumor immune responses and synergistically promotes ferroptotic death of cancer cells (Wang W. et al., 2019). On the other hand, ferroptosis inhibition therapy yielded greater antitumor efficacy when used in combination with anti-PD-1 antibodies [346].

However, ferroptosis can sometimes promote tumor initiation and progression (Dang et al., 2022). Ferroptosis-induced inflammation may drive necroinflammation-associated malignancies, and immune cell susceptibility to ferroptosis can undermine tumor suppression or promote tumor development (Bell et al., 2024). Ferroptotic cancer cells may also have immunosuppressive effects that enhance tumor growth (Chen X. et al., 2021; Qi and Peng, 2023). In addition, the ferroptosis of non-tumor cells is linked to a diminished ability to combat tumors due to a decrease in the generation of cytotoxic cytokines. Utilizing the ferroptosis inhibitor ferrostatin-1 effectively prevents CD8^+^ T cell ferroptosis by inhibiting lipid peroxidation (Wang W. et al., 2019). As a result, the production of pro-inflammatory cytokines is enhanced, leading to the elimination of tumors. Ferroptosis inhibition yields enhanced antitumor effectiveness when combined with anti-PD-1 antibodies (Ma et al., 2021). Further, Inhibiting ferroptosis could mitigate adverse effects from therapies promoting it, suggesting its suppression might be a viable cancer treatment strategy in certain contexts.

Currently, a phase II clinical study is assessing the ferroptosis inhibitor MIT-001 for preventing oral mucositis in lymphoma or multiple myeloma patients undergoing conditioning chemotherapy with autologous hematopoietic stem cell transplantation (NCT05493800).

### 1.6 New roles for cuproptosis in cancer cell death and targeting strategies

In 2019, Tsvetkov et al. discovered Cu-dependent death while investigating the anticancer mechanism of the Cu ionophore elesclomol (Tsvetkov et al., 2019). It was discovered that administering elesclomol to a mouse model of multiple myeloma decreased the cancer cells’ resistance to the damage caused by proteasome inhibitors. Mechanistically, reduced Cu(I) is produced when elesclomol-bound Cu(II) interacts with the mitochondrial enzyme ferredoxin 1 (FDX1), raising ROS levels (Nagai et al., 2012; Tsvetkov et al., 2019). Lipid peroxidation was once thought to be the cause of elesclomol’s lethality (Gao et al., 2021). Later, in 2022, they reported that intracellular copper build-up causes mitochondrial lipoylated protein oligomerization and destabilizes Fe–S cluster proteins, resulting in cuproptosis, an independent mode of cell death that is different from other RCD pathways (Ge et al., 2022). Research in the domains of cancer pathology and cell physiology has long focused on the role of copper in tumor progression, with studies emphasizing the critical connection between cuproptosis and cancer. Tumor angiogenesis and metastasis are activated by copper, a proangiogenic factor (Xu et al., 2022). Dysfunctional copper metabolism is the cause of both radioresistance and chemoresistance (Liu et al., 2022; Yang et al., 2022). Increased serum copper levels have been linked in a number of studies to disease invasion and tumor stage in patients with breast, lung, and colorectal cancer (Baszuk et al., 2021; Cui et al., 2021; Tsang et al., 2022). On the other hand, cuproptosis causes endothelial cell dysfunction, oxidative stress, and mitochondrial damage in malignant cells by interfering with lipid metabolism (Halliwell and Chirico, 1993; Ruiz et al., 2021).

Further, elevated Cu has been strongly associated with the increased expression level of hypoxia-inducible factor 1α (Feng et al., 2009; Wu Z. et al., 2019), inducing angiogenesis, and neovascularization leading to increased production of vascular endothelial growth factor (Zimna and Kurpisz, 2015). Elevated expression of intracellular Cu-dependent protein MEMO1, an oncogenic protein, has been associated with migration and invasion of breast and lung cancer cells (MacDonald et al., 2014). Zhang et al. have demonstrated that MEMO1 preferentially binds to Cu(I) and not Cu(II) and thus protects cells from redox activity (Zhang et al., 2022d). Consequently, releasing Cu ions and preventing the spread of tumor cells may be achieved by devising a suitable strategy to disrupt the Cu(I) binding site on the MEMO1 protein.

Cuproptosis may prevent the spread of cancer cells and reduce their proliferation (Li J. et al., 2022; Feng et al., 2024). Cuproptotic tumors exhibit reduced angiogenesis and respond well to therapy with sunitinib and sorafenib (Li K. et al., 2022). Cancer cells have developed mechanisms to defend against Cu-induced apoptosis (Table 1). For example, individuals with hepatocellular carcinoma (HCC) had significantly reduced levels of the critical cuproptosis regulator FDX1, making HCC cells resistant to cuproptosis (Zhang Z. et al., 2022). More advanced tumor-node-metastasis stages are closely linked to reduced FDX1 expression. Additionally, shorter survival rates have been associated with decreased FDX1 expression across various cancer types (Wang T. et al., 2022).

Copper ionophores, or cuproptosis-related drugs which trigger cuproptosis, may hold promise for future tumor treatments (Table 7) (Springer et al., 2024; Wang Y. et al., 2024). Elesclomol (ES) and Disulfiram (DFS) induce apoptosis by transporting copper ions into cells and mitochondria, resulting in the oligomerization of dihydrolipoamide s-acetyltransferase, decreased stability of Fe-S clusters and interaction with Npl4 (Reeder et al., 2011). Copper complexes with bis(thiosemicarbazone) ligands raise copper ion levels in both cancer cells and in Chlamydia-infected host cells (Cater et al., 2013; Marsh et al., 2017). Furthermore, derivatives of quinolines also function as copper ionophores (Oliveri et al., 2017; Oliveri, 2022). Derivatives from simple compounds such as 3-Hydroxyflavone (Dai et al., 2017), as well as more intricate copper ionophores like Hydrophilic Temperature-Sensitive Liposomes (Gaal et al., 2020) and a copper ionophore designed using salicylaldehyde isonicotinoyl hydrazone (Ji et al., 2018), also increase copper levels inside cells.

**TABLE 7:** Cuproptosis targeting drugs for cancer therapy.

Drug/Treatment	Target/Pathway	Cancer Type(s)	Clinical status	References
Elesclomol (ES)	Acts as a copper ionophore, increasing intracellular copper levels	Melanoma, Lung Cancer, Lymphoma	Phase III trial (for melanoma) in combination with chemotherapy for advanced melanoma.	O'Day et al. (2013), Tsvetkov et al. (2022)
Disulfiram (DFS)	Forms a complex with copper, inducing proteasome inhibition	Breast Cancer, Prostate Cancer, Glioblastoma	Phase I clinical trial	Kelley et al. (2021)
Tetrathiomolybdate (TM)	Copper chelator that depletes systemic copper,	Breast Cancer, Kidney cancer	Phase II clinical trial	Brewer et al. (2000), Redman et al. (2003), Chan et al. (2017)
D-Penicillamine	Chelates copper, reducing intracellular levels	Lung cancer, Breast cancer	Preclinical	Sciegienka et al. (2017)
Trientine	Copper chelator reducing systemic copper levels	Various Cancers	Preclinical/Clinical Trials	Yoshii et al. (2001), Huang et al. (2019b)
Copper-64 Radiopharmaceuticals	Utilize radioactive copper isotopes for imaging and targeted radiotherapy, affecting cuproptosis pathways	Neuroendocrine Tumors, Prostate Cancer	Clinical Trial	Boschi et al. (2018), Zhou et al. (2019)
Copper Oxide Nanoparticles	Increase intracellular copper levels, inducing oxidative stress	Breast.colotectalr yhrg Colon cancer	Preclinical	Benguigui et al. (2019), Ghasemi et al. (2023), Abdollahzadeh et al. (2024)
Ammonium Tetrathiomolybdate	Copper chelator that reduces angiogenesis and metastasis	Breast Cancer, Lung Cancer	Phase I/II Clinical Trials	Chisholm et al. (2016)

Among these agents, Elesclomol (ES) and Disulfiram (DFS) are currently undergoing evaluation in clinical trials (Xie J. et al., 2023). Recent trials investigating ES (O'Day et al., 2013) and DSF (Kelley et al., 2021) have demonstrated excellent safety profiles. Current research in this area is focused on nanomedicines that combine copper ions with copper ionophores (Lee et al., 2023; Zhou et al., 2023). Combining other cancer treatments with cuproptosis-related therapy may yield improved outcomes. Overall, copper ionophores may have greater efficacy in tumors with elevated mitochondrial metabolism. In the phase III clinical trial of ES, the impact of ES varied among individuals with low serum LDH levels (O'Day et al., 2013). Thus, serum LDH levels may serve as a prognostic indicator in the future clinical use of cuproptosis-related medications, helping to assess the potential effectiveness of these drugs. To summarize, copper ionophores can be combined with targeted therapeutic agents like TKI and PI. This combination is most effective in tumors with high mitochondrial metabolic status. Additionally, LDH can be used as a predictor to guide treatment before drug administration and as a prognostic indicator afterward. Further research is necessary to ascertain the feasibility of cuproptosis-inducing therapies in select patients with distinct types of cancer.

### 1.7 Parthanatos as target in cancer treatment

Parthanatos is a cell death mechanism controlled by PARP-1 and is distinct from apoptosis and necroptosis (Harraz et al., 2008) (Figures 1, 2). In parthanatos, abnormal PARP-1 activation causes excessive PAR production (Dawson and Dawson, 2004), mitochondrial membrane depolarization decreases ATP and NADPH levels, and triggers AIF translocation from mitochondria to the nucleus. Additionally, AIF binds to MIF nuclease, activating it (Wang Y. et al., 2019). After translocating to the nucleus, AIF and MIF cause nuclear shrinkage, chromatin agglutination, and big DNA fragments (15–50 KB) that cause parthanatos (Zhou et al., 2021). The lack of caspase is its main characteristic.

There is a strong correlation between parthanatos and tumor formation and progression (Zhou et al., 2021). The expression level of PARP-1 in breast cancer, ovarian cancer, endometrial cancer, lung cancer, skin cancer and non-Hodgkin’s lymphoma is elevated compared to normal tissues, thus establishing a strong association between parthanatos and these cancers (Harraz et al., 2008; Fong et al., 2009; Galia et al., 2012; Dorsam et al., 2018; Pazzaglia and Pioli, 2019). PARP-1 knockout mice showed a considerable decrease in susceptibility to epithelial malignancies. Downregulating PARP-1 protein hinders the action of NF-κB and the expression of tumor-promoting proteins controlled by NF-κB, thereby preventing the induction of parthanatos (Pazzaglia and Pioli, 2019). Additionally, the absence of PARP-1 in mice resulted in a notable decrease in the occurrence of colorectal cancer caused by oxymethane (AOM) and dextran sulfate sodium (DSS). Reducing PARP-1 protein levels may effectively prevent induced colorectal cancer by suppressing the expression of cyclin D and STAT3 (Dorsam et al., 2018).

The impact of parthanatos on carcinogenesis and tumor development manifests in two key dimensions (Zhou et al., 2021). During rapid cellular proliferation, DNA is highly susceptible to radiotherapy or chemotherapy, leading to tumor cell death. PARP-1 plays a crucial role in DNA repair and is essential for tumor cell survival. Hence, inducing apoptosis in tumor cells can be achieved by suppressing PARP-1 activity. Conversely, the occurrence of parthanatos primarily arises from the abnormal activation of PARP-1. Promoting parthanatos in tumor cells by augmenting PARP-1 activity can impede tumor cell proliferation. Given PARP-1’s involvement in several DNA repair pathways and its role in maintaining genomic stability (Yang et al., 2020), modulating PARP-1 activity may be therapeutic for treating associated malignancies (Table 8).

**TABLE 8:** Parthanatos targeting drugs for cancer therapy.

Drug/Treatment	Target/Pathway	Cancer Type(s)	Clinical status	References
Olaparib (Lynparza)	PARP inhibitor; inhibits PARP-1/2 leading to DNA damage accumulation	BRCA-mutated Ovarian Cancer, Breast Cancer pancreatic cancer, and prostate cancer	FDA approved	Clarke et al. (2024), Fenton and Hussain (2024), Kawamoto et al. (2024), Lee et al. (2024), Shah et al. (2024)
Rucaparib (Rubraca)	PARP inhibitor	BRCA-mutated Ovarian Cancer, Prostate Cancer	FDA approved	Monk et al. (2022), Sayyid et al. (2024)
Niraparib (Zejula)	PARP inhibitor inducing parthanatos via DNA damage accumulation	Ovarian Cancer	FDA approved	Wu et al. (2024b)
Veliparib (ABT-888)	PARP inhibitor inducing parthanatos; used in combination therapies	Various Cancers	Phase II/III Clinical Trials	Mizuno et al. (2023), Rodler et al. (2023), Zhao et al. (2023), Dieras et al. (2024), Kashbour et al. (2024), Sun and Li (2024)
Talazoparib (Talzenna)	PARP inhibitor with strong PARP-trapping ability	BRCA-mutated Breast Cancer, prostate cancer	FDA approved	Fizazi et al. (2024), Heiss et al. (2024), Narang et al. (2024), Piha-Paul et al. (2024), Telli et al. (2024)
β-Lapachone	PARP inducer	Hepatocellular carcinoma	preclinical	Zhao et al. (2021a)
Deoxypodophyllotoxin (DPT)	PARP inducer	Glioma	preclinical	Ma et al. (2016a)
PJ34	PARP inhibitor	Various Cancers	Preclinical	Shi et al. (2014b)
CEP-8983	PARP inhibitor	leukemia	Preclinical	Dilley et al. (2014)
E7016 (GPI 21016)	PARP inhibitor	Solid Tumors	Phase I Clinical Trials	Russo et al. (2009)
INO-1001	PARP inhibitor	Melanoma, Glioblastoma	Preclinical	Mason et al. (2008)

In clinical trials, PARP inhibitors are mostly administered to cancer patients with homologous recombination repair deficiencies including those with breast and ovarian cancers carrying BRCA1 and BRCA2 mutations (gBRCA1/2m) and castration-resistant prostate cancer. Currently, Olaparib (Clarke et al., 2024; Fenton and Hussain, 2024; Kawamoto et al., 2024; Lee et al., 2024; Shah et al., 2024), niraparib (Wu X. et al., 2024), rucaparib (Monk et al., 2022; Sayyid et al., 2024), veliparib (Mizuno et al., 2023; Rodler et al., 2023; Zhao et al., 2023; Dieras et al., 2024; Kashbour et al., 2024; Sun and Li, 2024) and talazoparib (Fizazi et al., 2024; Heiss et al., 2024; Narang et al., 2024; Piha-Paul et al., 2024; Telli et al., 2024) hinder the cancer-fighting effects of parthanatos by suppressing the catalytic function of PARP-1 and PARP-2 (Fong et al., 2009; Sandhu et al., 2013; Mateo et al., 2016; de Bono et al., 2017; Nishikawa et al., 2017; Wu X. et al., 2024).

β-Lapachone, a naturally occurring compound derived from the bark of the lapacho tree, triggers parthanatos by activating the NQO1-dependent ROS-mediated RIPK1-PARP1-AIF pathway, leading to the death of hepatocellular carcinoma cells (Zhao W. et al., 2021). This process was prevented by the inclusion of a PARP-1-specific inhibitor (Park et al., 2014). Deoxypodophyllotoxin (DPT), a naturally occurring chemical derived from Anthriscus sylvestris, effectively suppressed glioma growth by promoting the generation of excessive reactive oxygen species (ROS), enhancing PARP-1 expression and facilitating AIF translocation to the cell nucleus. This has been shown in both xenograft glioma models and in glioma cells cultured *in vitro* (Ma D. et al., 2016).

## 2 Conclusion and future perspective

The evolution of cancer therapy always involves trial and error, but discovery of novel mechanisms to target the mission-critical events shared by all tumors offers a glimpse of previously unthinkable therapeutic possibilities (Debela et al., 2021; Levantini, 2023). Understanding carcinogenesis, especially through the identification of altered cellular processes that maintain cancer cells and the development of diagnostic and prognostic biomarkers, has been made possible by studying these altered cell death pathways (Koren and Fuchs, 2021; Peng et al., 2022; Zhou Y. et al., 2024). Since RCD pathways are fundamental to the genesis of all tumors, they present clear targets for therapeutic intervention in all cancer types (Koren and Fuchs, 2021; Peng et al., 2022; Gong et al., 2023). Moreover, detecting abnormalities in these signaling pathways can aid in identifying the DNA, mRNA and protein mutations present in cancer cells, and may play a significant role in determining the efficacy of specific targeted therapies (Waarts et al., 2022; Chitluri and Emerson, 2024; Liu B. et al., 2024). While a tumor’s mutational profile may impact a therapy’s effectiveness, identifying altered RCD pathways may yield identification of novel targets.

The complexity of the cellular signaling that occurs in tumor cells presents the biggest obstacle to addressing the dysregulated pathways in distinct cancers (Bou Antoun and Chioni, 2023; Swanton et al., 2024). Crosstalk and inhibitory feedback mechanisms are just two examples of the many elements that obstruct targeted signaling pathways. Additionally, the risk of resistance selection exists with all tumor therapies and this risk may be exacerbated by the genetic plasticity present in most malignancies (Emran et al., 2022; Khan et al., 2024).

The primary therapeutic challenge in targeting RCD pathways for cancer treatment lies in the emergence of resistance mechanisms (D'Amico and De Amicis, 2024). Cancer cells often experience genetic and epigenetic changes that enable them to evade or inhibit cell death signals, even in the presence of targeted therapies designed to activate these pathways (Ozyerli-Goknar and Bagci-Onder, 2021; Tufail et al., 2024). For instance, the overexpression of anti-apoptotic proteins like BCL-2 and BCL-XL or mutations in tumor suppressors such as TP53 can inhibit apoptosis, allowing cancer cells to escape death induced by chemotherapy (Mohammad et al., 2015). In a similar vein, autophagy—a mechanism that enables cells to survive under stress—can be exploited by cancer cells to endure therapeutic damage, resulting in certain cancers, such as pancreatic and lung cancer, becoming resistant to drugs aimed at metabolic pathways (Li et al., 2019; Mele et al., 2020).

Ferroptosis, serves as another significant instance where resistance develops (Nie et al., 2022). The overexpression of GPX4, a lipid peroxidase enzyme, diminishes oxidative stress and inhibits ferroptosis-mediated cell death (Xie Y. et al., 2023), enabling cells to escape therapies aimed at triggering this type of RCD, particularly in liver and pancreatic cancers. Resistance to necroptosis, arises from the inactivation of essential regulators such as RIPK1 and RIPK3, resulting in treatment resistance in cancers including glioblastoma (Xie Y. et al., 2023) and colorectal cancer (Feng et al., 2015). In cuproptosis, cancer cells evade copper-induced cell death by disrupting copper ion homeostasis, with changes in proteins such as FDX1 and DLAT contributing to resistance in lung and melanoma cancers (Abdullah et al., 2024).

Moreover, pyroptosis, can be inhibited by the dysregulation of inflammasome components such as NLRP3 and caspase-1 (Zheng M. et al., 2020). This enables cancer cells to evade the inflammatory response typically associated with pyroptosis. This evasion mechanism has been noted in cancers including colorectal, gastric, and breast cancer. Ultimately, parthanatos, associated with the overactivation of PARP1 due to DNA damage, is often evaded in breast and ovarian cancers by the overexpression of PARP1 or mutations in related pathways, which diminishes the effectiveness of PARP inhibitors in these instances (Pazzaglia and Pioli, 2019).

These examples illustrate how cancer cells’ capacity to manipulate and resist RCD pathways complicates therapeutic strategies. The flexibility and redundancy in cell death mechanisms necessitate the creation of combination therapies or innovative strategies to re-sensitize cancer cells, highlighting the importance of addressing these resistance mechanisms across different cancers. Furthermore, recent high-throughput sequencing data demonstrate the significance of these dysregulated signaling pathways in sustaining supportive TMEs that facilitate the growth and metastasis of numerous solid tumors (Wang et al., 2023). Understanding the composition and function of the TME is thus crucial for deciphering the impact of genetic and epigenetic changes that occur in tumors and the cells that surround them. By studying various tumor types, researchers may identify common pathways that contribute to tumor development.

While the caveats associated with targeting RCD pathways for cancer therapies described above are challenging, the most effective approach to address these issues likely requires use of more advanced combination therapies that target multiple lesions unique to tumors simultaneously. Building a pathway interaction network to determine the functional dependencies between different signaling pathways may offer new perspectives on disease causes and lead to development of more effective drug formulations. Future research should place a stronger emphasis on the utilization of combination therapies for studies employing patient-derived xenografts, organoids/tumoroids and genetically modified mouse models to target oncogenic signaling pathways, RCD and the TME.

## References

[B1] Abdollahzadeh H. Pazhang Y. Zamani A. Sharafi Y. (2024). Green synthesis of copper oxide nanoparticles using walnut shell and their size dependent anticancer effects on breast and colorectal cancer cell lines. Sci. Rep. 14 (1), 20323. 10.1038/s41598-024-71234-4 39223184 PMC11369244

[B2] Abdullah K. M. Kaushal J. B. Takkar S. Sharma G. Alsafwani Z. W. Pothuraju R. (2024). Copper metabolism and cuproptosis in human malignancies: unraveling the complex interplay for therapeutic insights. Heliyon 10 (5), e27496. 10.1016/j.heliyon.2024.e27496 38486750 PMC10938126

[B3] Agalakova N. I. (2024). Chloroquine and chemotherapeutic compounds in experimental cancer treatment. Int. J. Mol. Sci. 25 (2), 945. 10.3390/ijms25020945 38256019 PMC10815352

[B4] Ahmadi-Dehlaghi F. Mohammadi P. Valipour E. Pournaghi P. Kiani S. Mansouri K. (2023). Autophagy: a challengeable paradox in cancer treatment. Cancer Med. 12 (10), 11542–11569. 10.1002/cam4.5577 36760166 PMC10242330

[B5] Akhtar F. Bokhari S. R. A. (2024). Apoptosis. Treasure Island (FL): StatPearls.

[B6] Akin D. Wang S. K. Habibzadegah-Tari P. Law B. Ostrov D. Li M. (2014). A novel ATG4B antagonist inhibits autophagy and has a negative impact on osteosarcoma tumors. Autophagy 10 (11), 2021–2035. 10.4161/auto.32229 25483883 PMC4502682

[B7] Alam M. Alam S. Shamsi A. Adnan M. Elasbali A. M. Al-Soud W. A. (2022). Bax/Bcl-2 cascade is regulated by the EGFR pathway: therapeutic targeting of non-small cell lung cancer. Front. Oncol. 12, 869672. 10.3389/fonc.2022.869672 35402265 PMC8990771

[B8] Alameda J. P. Moreno-Maldonado R. Navarro M. Bravo A. Ramirez A. Page A. (2010). An inactivating CYLD mutation promotes skin tumor progression by conferring enhanced proliferative, survival and angiogenic properties to epidermal cancer cells. Oncogene 29 (50), 6522–6532. 10.1038/onc.2010.378 20838385

[B9] Aleksandrova K. V. Suvorova I. I. (2023). Evaluation of the effectiveness of various autophagy inhibitors in A549 cancer stem cells. Acta Naturae 15 (1), 19–25. 10.32607/actanaturae.11891 37153502 PMC10154774

[B10] Ali A. M. Atmaj J. Van Oosterwijk N. Groves M. R. Domling A. (2019). Stapled peptides inhibitors: a new window for target drug discovery. Comput. Struct. Biotechnol. J. 17, 263–281. 10.1016/j.csbj.2019.01.012 30867891 PMC6396041

[B11] Alidadi H. Ashtari A. Samimi A. Karami M. A. Khorsandi L. (2022). Myricetin loaded in solid lipid nanoparticles induces apoptosis in the HT-29 colorectal cancer cells via mitochondrial dysfunction. Mol. Biol. Rep. 49 (9), 8537–8545. 10.1007/s11033-022-07683-9 35767106

[B12] Amarante-Mendes G. P. Adjemian S. Branco L. M. Zanetti L. C. Weinlich R. Bortoluci K. R. (2018). Pattern recognition receptors and the host cell death molecular machinery. Front. Immunol. 9, 2379. 10.3389/fimmu.2018.02379 30459758 PMC6232773

[B13] Amaravadi R. K. Schilder R. J. Martin L. P. Levin M. Graham M. A. Weng D. E. (2015). A phase I study of the SMAC-mimetic birinapant in adults with refractory solid tumors or lymphoma. Mol. Cancer Ther. 14 (11), 2569–2575. 10.1158/1535-7163.MCT-15-0475 26333381

[B14] Amaravadi R. K. Winkler J. D. (2012). Lys05: a new lysosomal autophagy inhibitor. Autophagy 8 (9), 1383–1384. 10.4161/auto.20958 22878685 PMC3442884

[B15] Andreeff M. Kelly K. R. Yee K. Assouline S. Strair R. Popplewell L. (2016). Results of the phase I trial of RG7112, a small-molecule MDM2 antagonist in leukemia. Clin. Cancer Res. 22 (4), 868–876. 10.1158/1078-0432.CCR-15-0481 26459177 PMC4809642

[B16] Annibaldi A. Walczak H. (2020). Death receptors and their ligands in inflammatory disease and cancer. Cold Spring Harb. Perspect. Biol. 12 (9), a036384. 10.1101/cshperspect.a036384 31988141 PMC7461759

[B17] Bakar-Ates F. Ozkan E. (2024). Synergistic ferroptosis in triple-negative breast cancer cells: paclitaxel in combination with Erastin induced oxidative stress and Ferroportin-1 modulation in MDA-MB-231 cells. Naunyn Schmiedeb. Arch. Pharmacol. 10.1007/s00210-024-03523-8 39392483

[B18] Basuli D. Tesfay L. Deng Z. Paul B. Yamamoto Y. Ning G. (2017). Iron addiction: a novel therapeutic target in ovarian cancer. Oncogene 36 (29), 4089–4099. 10.1038/onc.2017.11 28319068 PMC5540148

[B19] Baszuk P. Marciniak W. Derkacz R. Jakubowska A. Cybulski C. Gronwald J. (2021). Blood copper levels and the occurrence of colorectal cancer in Poland. Biomedicines 9 (11), 1628. 10.3390/biomedicines9111628 34829856 PMC8615693

[B20] Bejarano E. Cuervo A. M. (2010). Chaperone-mediated autophagy. Proc. Am. Thorac. Soc. 7 (1), 29–39. 10.1513/pats.200909-102JS 20160146 PMC3137147

[B21] Bell H. N. Stockwell B. R. Zou W. (2024). Ironing out the role of ferroptosis in immunity. Immunity 57 (5), 941–956. 10.1016/j.immuni.2024.03.019 38749397 PMC11101142

[B22] Belyanskaya L. L. Marti T. M. Hopkins-Donaldson S. Kurtz S. Felley-Bosco E. Stahel R. A. (2007). Human agonistic TRAIL receptor antibodies Mapatumumab and Lexatumumab induce apoptosis in malignant mesothelioma and act synergistically with cisplatin. Mol. Cancer 6, 66. 10.1186/1476-4598-6-66 17953743 PMC2134932

[B23] Benguigui M. Weitz I. S. Timaner M. Kan T. Shechter D. Perlman O. (2019). Copper oxide nanoparticles inhibit pancreatic tumor growth primarily by targeting tumor initiating cells. Sci. Rep. 9 (1), 12613. 10.1038/s41598-019-48959-8 31471546 PMC6717199

[B24] Berke T. P. Slight S. H. Hyder S. M. (2022). Role of reactivating mutant p53 protein in suppressing growth and metastasis of triple-negative breast cancer. Onco Targets Ther. 15, 23–30. 10.2147/OTT.S342292 35035222 PMC8754468

[B25] Bhat G. R. Sethi I. Sadida H. Q. Rah B. Mir R. Algehainy N. (2024). Cancer cell plasticity: from cellular, molecular, and genetic mechanisms to tumor heterogeneity and drug resistance. Cancer Metastasis Rev. 43 (1), 197–228. 10.1007/s10555-024-10172-z 38329598 PMC11016008

[B26] Blagosklonny M. V. (2023). Cancer prevention with rapamycin. Oncotarget 14, 342–350. 10.18632/oncotarget.28410 37057884 PMC10103596

[B27] Blockhuys S. Celauro E. Hildesjo C. Feizi A. Stal O. Fierro-Gonzalez J. C. (2017). Defining the human copper proteome and analysis of its expression variation in cancers. Metallomics 9 (2), 112–123. 10.1039/c6mt00202a 27942658

[B28] Blockhuys S. Wittung-Stafshede P. (2017). Roles of copper-binding proteins in breast cancer. Int. J. Mol. Sci. 18 (4), 871. 10.3390/ijms18040871 28425924 PMC5412452

[B29] Bolden J. E. Peart M. J. Johnstone R. W. (2006). Anticancer activities of histone deacetylase inhibitors. Nat. Rev. Drug Discov. 5 (9), 769–784. 10.1038/nrd2133 16955068

[B30] Boschi A. Martini P. Janevik-Ivanovska E. Duatti A. (2018). The emerging role of copper-64 radiopharmaceuticals as cancer theranostics. Drug Discov. Today 23 (8), 1489–1501. 10.1016/j.drudis.2018.04.002 29635027

[B31] Bou Antoun N. Chioni A. M. (2023). Dysregulated signalling pathways driving anticancer drug resistance. Int. J. Mol. Sci. 24 (15), 12222. 10.3390/ijms241512222 37569598 PMC10418675

[B32] Boulos J. C. Rahama M. Hegazy M. F. Efferth T. (2019). Shikonin derivatives for cancer prevention and therapy. Cancer Lett. 459, 248–267. 10.1016/j.canlet.2019.04.033 31132429

[B33] Brewer G. J. Dick R. D. Grover D. K. LeClaire V. Tseng M. Wicha M. (2000). Treatment of metastatic cancer with tetrathiomolybdate, an anticopper, antiangiogenic agent: phase I study. Clin. Cancer Res. 6 (1), 1–10. 10656425

[B34] Brown J. S. Amend S. R. Austin R. H. Gatenby R. A. Hammarlund E. U. Pienta K. J. (2023). Updating the definition of cancer. Mol. Cancer Res. 21 (11), 1142–1147. 10.1158/1541-7786.MCR-23-0411 37409952 PMC10618731

[B35] Burvenich I. J. Lee F. T. Guo N. Gan H. K. Rigopoulos A. Parslow A. C. (2016). *In vitro* and *in vivo* evaluation of (89)Zr-DS-8273a as a theranostic for anti-death receptor 5 therapy. Theranostics 6 (12), 2225–2234. 10.7150/thno.16260 27924159 PMC5135445

[B36] Caenepeel S. Brown S. P. Belmontes B. Moody G. Keegan K. S. Chui D. (2018). AMG 176, a selective MCL1 inhibitor, is effective in hematologic cancer models alone and in combination with established therapies. Cancer Discov. 8 (12), 1582–1597. 10.1158/2159-8290.CD-18-0387 30254093

[B37] Cai Z. Jitkaew S. Zhao J. Chiang H. C. Choksi S. Liu J. (2014). Plasma membrane translocation of trimerized MLKL protein is required for TNF-induced necroptosis. Nat. Cell Biol. 16 (1), 55–65. 10.1038/ncb2883 24316671 PMC8369836

[B38] Cao L. Mu W. (2021). Necrostatin-1 and necroptosis inhibition: pathophysiology and therapeutic implications. Pharmacol. Res. 163, 105297. 10.1016/j.phrs.2020.105297 33181319 PMC7962892

[B39] Carew J. S. Nawrocki S. T. (2017). Drain the lysosome: development of the novel orally available autophagy inhibitor ROC-325. Autophagy 13 (4), 765–766. 10.1080/15548627.2017.1280222 28118053 PMC5388230

[B40] Carneiro B. A. El-Deiry W. S. (2020). Targeting apoptosis in cancer therapy. Nat. Rev. Clin. Oncol. 17 (7), 395–417. 10.1038/s41571-020-0341-y 32203277 PMC8211386

[B41] Cater M. A. Pearson H. B. Wolyniec K. Klaver P. Bilandzic M. Paterson B. M. (2013). Increasing intracellular bioavailable copper selectively targets prostate cancer cells. ACS Chem. Biol. 8 (7), 1621–1631. 10.1021/cb400198p 23656859

[B42] Chan N. Willis A. Kornhauser N. Ward M. M. Lee S. B. Nackos E. (2017). Influencing the tumor microenvironment: a phase II study of copper depletion using tetrathiomolybdate in patients with breast cancer at high risk for recurrence and in preclinical models of lung metastases. Clin. Cancer Res. 23 (3), 666–676. 10.1158/1078-0432.CCR-16-1326 27769988

[B43] Chang Y. S. Graves B. Guerlavais V. Tovar C. Packman K. To K. H. (2013). Stapled α-helical peptide drug development: a potent dual inhibitor of MDM2 and MDMX for p53-dependent cancer therapy. Proc. Natl. Acad. Sci. U. S. A. 110 (36), E3445–E3454. 10.1073/pnas.1303002110 23946421 PMC3767549

[B44] Chatran M. Pilehvar-Soltanahmadi Y. Dadashpour M. Faramarzi L. Rasouli S. Jafari-Gharabaghlou D. (2018). Synergistic anti-proliferative effects of metformin and silibinin combination on T47D breast cancer cells via hTERT and cyclin D1 inhibition. Drug Res. (Stuttg) 68 (12), 710–716. 10.1055/a-0631-8046 29920623

[B45] Chavez-Dominguez R. Perez-Medina M. Lopez-Gonzalez J. S. Galicia-Velasco M. Aguilar-Cazares D. (2020). The double-edge sword of autophagy in cancer: from tumor suppression to pro-tumor activity. Front. Oncol. 10, 578418. 10.3389/fonc.2020.578418 33117715 PMC7575731

[B46] Chen G. Ding X. F. Bouamar H. Pressley K. Sun L. Z. (2019). Everolimus induces G(1) cell cycle arrest through autophagy-mediated protein degradation of cyclin D1 in breast cancer cells. Am. J. Physiol. Cell Physiol. 317 (2), C244–C252. 10.1152/ajpcell.00390.2018 31116586 PMC6732424

[B47] Chen G. Y. Meng C. L. Lin K. C. Tuan H. Y. Yang H. J. Chen C. L. (2015). Graphene oxide as a chemosensitizer: diverted autophagic flux, enhanced nuclear import, elevated necrosis and improved antitumor effects. Biomaterials 40, 12–22. 10.1016/j.biomaterials.2014.11.034 25498801

[B48] Chen G. Y. Nunez G. (2010). Sterile inflammation: sensing and reacting to damage. Nat. Rev. Immunol. 10 (12), 826–837. 10.1038/nri2873 21088683 PMC3114424

[B49] Chen J. Ge L. Shi X. Liu J. Ruan H. Heng D. (2022). Lobaplatin induces pyroptosis in cervical cancer cells via the caspase-3/GSDME pathway. Anticancer Agents Med. Chem. 22 (11), 2091–2097. 10.2174/1871520621666211018100532 34666646

[B50] Chen L. Niu X. Qiao X. Liu S. Ma H. Shi X. (2021a). Characterization of interplay between autophagy and ferroptosis and their synergistical roles on manipulating immunological tumor microenvironment in squamous cell carcinomas. Front. Immunol. 12, 739039. 10.3389/fimmu.2021.739039 35185859 PMC8854375

[B51] Chen Q. Kang J. Fu C. (2018). The independence of and associations among apoptosis, autophagy, and necrosis. Signal Transduct. Target Ther. 3, 18. 10.1038/s41392-018-0018-5 29967689 PMC6026494

[B52] Chen X. Kang R. Kroemer G. Tang D. (2021b). Ferroptosis in infection, inflammation, and immunity. J. Exp. Med. 218 (6), e20210518. 10.1084/jem.20210518 33978684 PMC8126980

[B53] Cheng A. L. Kang Y. K. Chen Z. Tsao C. J. Qin S. Kim J. S. (2009). Efficacy and safety of sorafenib in patients in the Asia-Pacific region with advanced hepatocellular carcinoma: a phase III randomised, double-blind, placebo-controlled trial. Lancet Oncol. 10 (1), 25–34. 10.1016/S1470-2045(08)70285-7 19095497

[B54] Chisholm C. L. Wang H. Wong A. H. Vazquez-Ortiz G. Chen W. Xu X. (2016). Ammonium tetrathiomolybdate treatment targets the copper transporter ATP7A and enhances sensitivity of breast cancer to cisplatin. Oncotarget 7 (51), 84439–84452. 10.18632/oncotarget.12992 27806319 PMC5341295

[B55] Chitluri K. K. Emerson I. A. (2024). The importance of protein domain mutations in cancer therapy. Heliyon 10 (6), e27655. 10.1016/j.heliyon.2024.e27655 38509890 PMC10950675

[B56] Choi Y. K. Kang J. I. Han S. Kim Y. R. Jo J. Kang Y. W. (2020). L-ascorbic acid inhibits breast cancer growth by inducing IRE/JNK/CHOP-related endoplasmic reticulum stress-mediated p62/SQSTM1 accumulation in the nucleus. Nutrients 12 (5), 1351. 10.3390/nu12051351 32397306 PMC7284633

[B57] Ciuleanu T. Bazin I. Lungulescu D. Miron L. Bondarenko I. Deptala A. (2016). A randomized, double-blind, placebo-controlled phase II study to assess the efficacy and safety of mapatumumab with sorafenib in patients with advanced hepatocellular carcinoma. Ann. Oncol. 27 (4), 680–687. 10.1093/annonc/mdw004 26802147

[B58] Clarke N. W. Armstrong A. J. Oya M. Shore N. Procopio G. Daniel Guedes J. (2024). Efficacy and safety of olaparib plus abiraterone versus placebo plus abiraterone in the first-line treatment of patients with asymptomatic/mildly symptomatic and symptomatic metastatic castration-resistant prostate cancer: analyses from the phase 3 PROpel trial. Eur. Urol. Oncol. 10.1016/j.euo.2024.09.013 39384451

[B59] Cluzeau T. Sebert M. Rahme R. Cuzzubbo S. Lehmann-Che J. Madelaine I. (2021). Eprenetapopt Plus Azacitidine in TP53-Mutated Myelodysplastic Syndromes and Acute Myeloid Leukemia: A Phase II Study by the Groupe Francophone des Myelodysplasies (GFM). J. Clin. Oncol. 39 (14), 1575–1583. 10.1200/JCO.20.02342 33600210 PMC8099409

[B60] Cook K. L. Warri A. Soto-Pantoja D. R. Clarke P. A. Cruz M. I. Zwart A. (2014). Hydroxychloroquine inhibits autophagy to potentiate antiestrogen responsiveness in ER+ breast cancer. Clin. Cancer Res. 20 (12), 3222–3232. 10.1158/1078-0432.CCR-13-3227 24928945 PMC4073207

[B61] Cui L. Gouw A. M. LaGory E. L. Guo S. Attarwala N. Tang Y. (2021). Mitochondrial copper depletion suppresses triple-negative breast cancer in mice. Nat. Biotechnol. 39 (3), 357–367. 10.1038/s41587-020-0707-9 33077961 PMC7956242

[B62] Dabiri Y. Abu El Maaty M. A. Chan H. Y. Wolker J. Ott I. Wolfl S. (2019). p53-Dependent anti-proliferative and pro-apoptotic effects of a gold(I) N-heterocyclic carbene (NHC) complex in colorectal cancer cells. Front. Oncol. 9, 438. 10.3389/fonc.2019.00438 31231607 PMC6558413

[B63] Dai F. Yan W. J. Du Y. T. Bao X. Z. Li X. Z. Zhou B. (2017). Structural basis, chemical driving forces and biological implications of flavones as Cu(II) ionophores. Free Radic. Biol. Med. 108, 554–563. 10.1016/j.freeradbiomed.2017.04.023 28431962

[B64] D'Amico M. De Amicis F. (2024). Challenges of regulated cell death: implications for therapy resistance in cancer. Cells 13 (13), 1083. 10.3390/cells13131083 38994937 PMC11240625

[B65] Dang Q. Sun Z. Wang Y. Wang L. Liu Z. Han X. (2022). Ferroptosis: a double-edged sword mediating immune tolerance of cancer. Cell Death Dis. 13 (11), 925. 10.1038/s41419-022-05384-6 36335094 PMC9637147

[B66] Dawson V. L. Dawson T. M. (2004). Deadly conversations: nuclear-mitochondrial cross-talk. J. Bioenerg. Biomembr. 36 (4), 287–294. 10.1023/B:JOBB.0000041755.22613.8d 15377859

[B67] Debela D. T. Muzazu S. G. Heraro K. D. Ndalama M. T. Mesele B. W. Haile D. C. (2021). New approaches and procedures for cancer treatment: current perspectives. SAGE Open Med. 9, 20503121211034366. 10.1177/20503121211034366 34408877 PMC8366192

[B68] Debnath J. Gammoh N. Ryan K. M. (2023). Autophagy and autophagy-related pathways in cancer. Nat. Rev. Mol. Cell Biol. 24 (8), 560–575. 10.1038/s41580-023-00585-z 36864290 PMC9980873

[B69] de Bono J. Ramanathan R. K. Mina L. Chugh R. Glaspy J. Rafii S. (2017). Phase I, dose-escalation, two-Part Trial of the PARP inhibitor talazoparib in patients with advanced germline BRCA1/2 mutations and selected sporadic cancers. Cancer Discov. 7 (6), 620–629. 10.1158/2159-8290.CD-16-1250 28242752 PMC5905335

[B70] Degterev A. Huang Z. Boyce M. Li Y. Jagtap P. Mizushima N. (2005). Chemical inhibitor of nonapoptotic cell death with therapeutic potential for ischemic brain injury. Nat. Chem. Biol. 1 (2), 112–119. 10.1038/nchembio711 16408008

[B71] Del Gaizo Moore V. Brown J. R. Certo M. Love T. M. Novina C. D. Letai A. (2007). Chronic lymphocytic leukemia requires BCL2 to sequester prodeath BIM, explaining sensitivity to BCL2 antagonist ABT-737. J. Clin. Invest. 117 (1), 112–121. 10.1172/JCI28281 17200714 PMC1716201

[B72] Deng W. Xiong X. Lu M. Huang S. Luo Y. Wang Y. (2024). Curcumin suppresses colorectal tumorigenesis through restoring the gut microbiota and metabolites. BMC Cancer 24 (1), 1141. 10.1186/s12885-024-12898-z 39267014 PMC11395590

[B73] Devor E. J. Schickling B. M. Lapierre J. R. Bender D. P. Gonzalez-Bosquet J. Leslie K. K. (2021). The synthetic curcumin analog HO-3867 rescues suppression of PLAC1 expression in ovarian cancer cells. Pharm. (Basel) 14 (9), 942. 10.3390/ph14090942 PMC8465575 34577642

[B74] Dhuriya Y. K. Sharma D. (2018). Necroptosis: a regulated inflammatory mode of cell death. J. Neuroinflammation 15 (1), 199. 10.1186/s12974-018-1235-0 29980212 PMC6035417

[B75] Di Cosimo S. Perez-Garcia J. M. Bellet M. Dalenc F. Gil Gil M. J. Ruiz Borrego M. (2023). Palbociclib with fulvestrant or letrozole in endocrine-sensitive patients with HR-positive/HER2-negative advanced breast cancer: a detailed safety analysis of the randomized parsifal trial. Oncologist 28 (1), 23–32. 10.1093/oncolo/oyac205 36239405 PMC9847524

[B76] Dieras V. Han H. S. Wildiers H. Friedlander M. Ayoub J. P. Puhalla S. L. (2024). Veliparib with carboplatin and paclitaxel in BRCA-mutated advanced breast cancer (BROCADE3): final overall survival results from a randomized phase 3 trial. Eur. J. Cancer 200, 113580. 10.1016/j.ejca.2024.113580 38309017

[B77] Dilley R. L. Poh W. Gladstone D. E. Herman J. G. Showel M. M. Karp J. E. (2014). Poly(ADP-ribose) polymerase inhibitor CEP-8983 synergizes with bendamustine in chronic lymphocytic leukemia cells *in vitro* . Leuk. Res. 38 (3), 411–417. 10.1016/j.leukres.2013.12.019 24439051 PMC4142574

[B78] DiNardo C. D. Pratz K. Pullarkat V. Jonas B. A. Arellano M. Becker P. S. (2019). Venetoclax combined with decitabine or azacitidine in treatment-naive, elderly patients with acute myeloid leukemia. Blood 133 (1), 7–17. 10.1182/blood-2018-08-868752 30361262 PMC6318429

[B79] Ding J. Wang K. Liu W. She Y. Sun Q. Shi J. (2016). Pore-forming activity and structural autoinhibition of the gasdermin family. Nature 535 (7610), 111–116. 10.1038/nature18590 27281216

[B80] Ding P. Wen L. Tong F. Zhang R. Huang Y. Dong X. (2022). Mechanism underlying the immune checkpoint inhibitor-induced hyper-progressive state of cancer. Cancer Drug Resist 5 (1), 147–164. 10.20517/cdr.2021.104 35582541 PMC8992596

[B81] Ding Q. Zhang Z. Liu J. J. Jiang N. Zhang J. Ross T. M. (2013). Discovery of RG7388, a potent and selective p53-MDM2 inhibitor in clinical development. J. Med. Chem. 56 (14), 5979–5983. 10.1021/jm400487c 23808545

[B82] Dixon S. J. Lemberg K. M. Lamprecht M. R. Skouta R. Zaitsev E. M. Gleason C. E. (2012). Ferroptosis: an iron-dependent form of nonapoptotic cell death. Cell 149 (5), 1060–1072. 10.1016/j.cell.2012.03.042 22632970 PMC3367386

[B83] Dixon S. J. Patel D. N. Welsch M. Skouta R. Lee E. D. Hayano M. (2014). Pharmacological inhibition of cystine-glutamate exchange induces endoplasmic reticulum stress and ferroptosis. Elife 3, e02523. 10.7554/eLife.02523 24844246 PMC4054777

[B84] Dohner H. Pratz K. W. DiNardo C. D. Wei A. H. Jonas B. A. Pullarkat V. (2024). Genetic risk stratification and outcomes among treatment-naive patients with AML treated with venetoclax and azacitidine. Blood, 2024024944. 10.1182/blood.2024024944 PMC11600046 39133921

[B85] Doll S. Proneth B. Tyurina Y. Y. Panzilius E. Kobayashi S. Ingold I. (2017). ACSL4 dictates ferroptosis sensitivity by shaping cellular lipid composition. Nat. Chem. Biol. 13 (1), 91–98. 10.1038/nchembio.2239 27842070 PMC5610546

[B86] Dominguez G. A. Condamine T. Mony S. Hashimoto A. Wang F. Liu Q. (2017). Selective targeting of myeloid-derived suppressor cells in cancer patients using DS-8273a, an agonistic TRAIL-R2 antibody. Clin. Cancer Res. 23 (12), 2942–2950. 10.1158/1078-0432.CCR-16-1784 27965309 PMC5468499

[B87] Donohue E. Tovey A. Vogl A. W. Arns S. Sternberg E. Young R. N. (2011). Inhibition of autophagosome formation by the benzoporphyrin derivative verteporfin. J. Biol. Chem. 286 (9), 7290–7300. 10.1074/jbc.M110.139915 21193398 PMC3044985

[B88] Dorsam B. Seiwert N. Foersch S. Stroh S. Nagel G. Begaliew D. (2018). PARP-1 protects against colorectal tumor induction, but promotes inflammation-driven colorectal tumor progression. Proc. Natl. Acad. Sci. U. S. A. 115 (17), E4061–E4070. 10.1073/pnas.1712345115 29632181 PMC5924876

[B89] Draganov D. Gopalakrishna-Pillai S. Chen Y. R. Zuckerman N. Moeller S. Wang C. (2015). Modulation of P2X4/P2X7/Pannexin-1 sensitivity to extracellular ATP via Ivermectin induces a non-apoptotic and inflammatory form of cancer cell death. Sci. Rep. 5, 16222. 10.1038/srep16222 26552848 PMC4639773

[B90] Du J. Wang X. Li Y. Ren X. Zhou Y. Hu W. (2021a). DHA exhibits synergistic therapeutic efficacy with cisplatin to induce ferroptosis in pancreatic ductal adenocarcinoma via modulation of iron metabolism. Cell Death Dis. 12 (7), 705. 10.1038/s41419-021-03996-y 34262021 PMC8280115

[B91] Du Y. Zhao H. C. Zhu H. C. Jin Y. Wang L. (2021b). Ferroptosis is involved in the anti-tumor effect of lycorine in renal cell carcinoma cells. Oncol. Lett. 22 (5), 781. 10.3892/ol.2021.13042 34594422 PMC8456505

[B92] Dubuisson A. Micheau O. (2017). Antibodies and derivatives targeting DR4 and DR5 for cancer therapy. Antibodies (Basel) 6 (4), 16. 10.3390/antib6040016 31548531 PMC6698863

[B93] Duffy M. J. Murray A. Synnott N. C. O'Donovan N. Crown J. (2017). Vitamin D analogues: potential use in cancer treatment. Crit. Rev. Oncol. Hematol. 112, 190–197. 10.1016/j.critrevonc.2017.02.015 28325259

[B94] Dunkle A. He Y. W. (2011). Apoptosis and autophagy in the regulation of T lymphocyte function. Immunol. Res. 49 (1-3), 70–86. 10.1007/s12026-010-8195-5 21128005 PMC3248808

[B95] Eling N. Reuter L. Hazin J. Hamacher-Brady A. Brady N. R. (2015). Identification of artesunate as a specific activator of ferroptosis in pancreatic cancer cells. Oncoscience 2 (5), 517–532. 10.18632/oncoscience.160 26097885 PMC4468338

[B96] Elmore S. (2007). Apoptosis: a review of programmed cell death. Toxicol. Pathol. 35 (4), 495–516. 10.1080/01926230701320337 17562483 PMC2117903

[B97] Emran T. B. Shahriar A. Mahmud A. R. Rahman T. Abir M. H. Siddiquee M. F. (2022). Multidrug resistance in cancer: understanding molecular mechanisms, immunoprevention and therapeutic approaches. Front. Oncol. 12, 891652. 10.3389/fonc.2022.891652 35814435 PMC9262248

[B98] Erkes D. A. Cai W. Sanchez I. M. Purwin T. J. Rogers C. Field C. O. (2020). Mutant BRAF and MEK inhibitors regulate the tumor immune microenvironment via pyroptosis. Cancer Discov. 10 (2), 254–269. 10.1158/2159-8290.CD-19-0672 31796433 PMC7007378

[B99] Fang Y. Tian S. Pan Y. Li W. Wang Q. Tang Y. (2020). Pyroptosis: a new frontier in cancer. Biomed. Pharmacother. 121, 109595. 10.1016/j.biopha.2019.109595 31710896

[B100] Fantone S. Piani F. Olivieri F. Rippo M. R. Sirico A. Di Simone N. (2024). Role of slc7a11/xCT in ovarian cancer. Int. J. Mol. Sci. 25 (1), 587. 10.3390/ijms25010587 38203758 PMC10779187

[B101] Feldmann F. Schenk B. Martens S. Vandenabeele P. Fulda S. (2017). Sorafenib inhibits therapeutic induction of necroptosis in acute leukemia cells. Oncotarget 8 (40), 68208–68220. 10.18632/oncotarget.19919 28978109 PMC5620249

[B102] Feng S. Fox D. Man S. M. (2018). Mechanisms of gasdermin family members in inflammasome signaling and cell death. J. Mol. Biol. 430 (18 Pt B), 3068–3080. 10.1016/j.jmb.2018.07.002 29990470

[B103] Feng W. Shi W. Liu S. Liu H. Liu Y. Ge P. (2022). Fe(III)-Shikonin supramolecular nanomedicine for combined therapy of tumor via ferroptosis and necroptosis. Adv. Healthc. Mater 11 (2), e2101926. 10.1002/adhm.202101926 34738742

[B104] Feng W. Ye F. Xue W. Zhou Z. Kang Y. J. (2009). Copper regulation of hypoxia-inducible factor-1 activity. Mol. Pharmacol. 75 (1), 174–182. 10.1124/mol.108.051516 18842833 PMC2685058

[B105] Feng X. Song Q. Yu A. Tang H. Peng Z. Wang X. (2015). Receptor-interacting protein kinase 3 is a predictor of survival and plays a tumor suppressive role in colorectal cancer. Neoplasma 62 (4), 592–601. 10.4149/neo_2015_071 25997957

[B106] Feng Y. He D. Yao Z. Klionsky D. J. (2014). The machinery of macroautophagy. Cell Res. 24 (1), 24–41. 10.1038/cr.2013.168 24366339 PMC3879710

[B107] Feng Y. Yang Z. Wang J. Zhao H. (2024). Cuproptosis: unveiling a new frontier in cancer biology and therapeutics. Cell Commun. Signal 22 (1), 249. 10.1186/s12964-024-01625-7 38693584 PMC11064406

[B108] Fenton S. E. Hussain M. (2024). Olaparib monotherapy or in combination with abiraterone for treating mutated metastatic castration-resistant prostate cancer: alone or stronger together? Expert Opin. Investig. Drugs 33 (10), 993–999. 10.1080/13543784.2024.2391828 39135527

[B109] Fizazi K. Azad A. A. Matsubara N. Carles J. Fay A. P. De Giorgi U. (2024). First-line talazoparib with enzalutamide in HRR-deficient metastatic castration-resistant prostate cancer: the phase 3 TALAPRO-2 trial. Nat. Med. 30 (1), 257–264. 10.1038/s41591-023-02704-x 38049622 PMC10803259

[B110] Fong P. C. Boss D. S. Yap T. A. Tutt A. Wu P. Mergui-Roelvink M. (2009). Inhibition of poly(ADP-ribose) polymerase in tumors from BRCA mutation carriers. N. Engl. J. Med. 361 (2), 123–134. 10.1056/NEJMoa0900212 19553641

[B111] Forero A. Bendell J. C. Kumar P. Janisch L. Rosen M. Wang Q. (2017). First-in-human study of the antibody DR5 agonist DS-8273a in patients with advanced solid tumors. Invest. New Drugs 35 (3), 298–306. 10.1007/s10637-016-0420-1 28050790

[B112] Forero-Torres A. Infante J. R. Waterhouse D. Wong L. Vickers S. Arrowsmith E. (2013). Phase 2, multicenter, open-label study of tigatuzumab (CS-1008), a humanized monoclonal antibody targeting death receptor 5, in combination with gemcitabine in chemotherapy-naive patients with unresectable or metastatic pancreatic cancer. Cancer Med. 2 (6), 925–932. 10.1002/cam4.137 24403266 PMC3892397

[B113] Forero-Torres A. Shah J. Wood T. Posey J. Carlisle R. Copigneaux C. (2010). Phase I trial of weekly tigatuzumab, an agonistic humanized monoclonal antibody targeting death receptor 5 (DR5). Cancer Biother Radiopharm. 25 (1), 13–19. 10.1089/cbr.2009.0673 20187792 PMC2883819

[B114] Franchi L. Munoz-Planillo R. Nunez G. (2012). Sensing and reacting to microbes through the inflammasomes. Nat. Immunol. 13 (4), 325–332. 10.1038/ni.2231 22430785 PMC3449002

[B115] Frenzel A. Grespi F. Chmelewskij W. Villunger A. (2009). Bcl2 family proteins in carcinogenesis and the treatment of cancer. Apoptosis 14 (4), 584–596. 10.1007/s10495-008-0300-z 19156528 PMC3272401

[B116] Fulda S. Vucic D. (2012). Targeting IAP proteins for therapeutic intervention in cancer. Nat. Rev. Drug Discov. 11 (2), 109–124. 10.1038/nrd3627 22293567

[B117] Gaal A. Garay T. M. Horvath I. Mathe D. Szollosi D. Veres D. S. (2020). Development and *in vivo* application of a water-soluble anticancer copper ionophore system using a temperature-sensitive liposome formulation. Pharmaceutics 12 (5), 466. 10.3390/pharmaceutics12050466 32443790 PMC7284829

[B118] Galia A. Calogero A. E. Condorelli R. Fraggetta F. La Corte A. Ridolfo F. (2012). PARP-1 protein expression in glioblastoma multiforme. Eur. J. Histochem 56 (1), e9. 10.4081/ejh.2012.e9 22472897 PMC3352138

[B119] Galluzzi L. Vitale I. Aaronson S. A. Abrams J. M. Adam D. Agostinis P. (2018). Molecular mechanisms of cell death: recommendations of the nomenclature committee on cell death 2018. Cell Death Differ. 25 (3), 486–541. 10.1038/s41418-017-0012-4 29362479 PMC5864239

[B120] Gan L. Wang J. Xu H. Yang X. (2011). Resistance to docetaxel-induced apoptosis in prostate cancer cells by p38/p53/p21 signaling. Prostate 71 (11), 1158–1166. 10.1002/pros.21331 21656826

[B121] Gandhi L. Camidge D. R. Ribeiro de Oliveira M. Bonomi P. Gandara D. Khaira D. (2011). Phase I study of Navitoclax (ABT-263), a novel Bcl-2 family inhibitor, in patients with small-cell lung cancer and other solid tumors. J. Clin. Oncol. 29 (7), 909–916. 10.1200/JCO.2010.31.6208 21282543 PMC4668282

[B122] Gao W. Huang Z. Duan J. Nice E. C. Lin J. Huang C. (2021). Elesclomol induces copper-dependent ferroptosis in colorectal cancer cells via degradation of ATP7A. Mol. Oncol. 15 (12), 3527–3544. 10.1002/1878-0261.13079 34390123 PMC8637554

[B123] Gardner B. M. Pincus D. Gotthardt K. Gallagher C. M. Walter P. (2013). Endoplasmic reticulum stress sensing in the unfolded protein response. Cold Spring Harb. Perspect. Biol. 5 (3), a013169. 10.1101/cshperspect.a013169 23388626 PMC3578356

[B124] Gaschler M. M. Andia A. A. Liu H. Csuka J. M. Hurlocker B. Vaiana C. A. (2018). FINO(2) initiates ferroptosis through GPX4 inactivation and iron oxidation. Nat. Chem. Biol. 14 (5), 507–515. 10.1038/s41589-018-0031-6 29610484 PMC5899674

[B125] Ge E. J. Bush A. I. Casini A. Cobine P. A. Cross J. R. DeNicola G. M. (2022). Connecting copper and cancer: from transition metal signalling to metalloplasia. Nat. Rev. Cancer 22 (2), 102–113. 10.1038/s41568-021-00417-2 34764459 PMC8810673

[B126] Ghasemi P. Shafiee G. Ziamajidi N. Abbasalipourkabir R. (2023). Copper nanoparticles induce apoptosis and oxidative stress in SW480 human colon cancer cell line. Biol. Trace Elem. Res. 201 (8), 3746–3754. 10.1007/s12011-022-03458-2 36274109

[B127] Gill H. (2024). Chemotherapy-free approaches to newly-diagnosed acute promyelocytic leukaemia: is oral-arsenic trioxide/all-trans retinoic acid/ascorbic acid the answer? Expert Rev. Hematol. 17 (10), 661–667. 10.1080/17474086.2024.2391098 39120131

[B128] Gill S. Brudno J. N. (2021). CAR T-cell therapy in hematologic malignancies: clinical role, toxicity, and unanswered questions. Am. Soc. Clin. Oncol. Educ. Book 41, 1–20. 10.1200/EDBK_320085 33989023

[B129] Giordano F. D'Amico M. Montalto F. I. Malivindi R. Chimento A. Conforti F. L. (2023). Cdk4 regulates glioblastoma cell invasion and stemness and is target of a notch inhibitor plus resveratrol combined treatment. Int. J. Mol. Sci. 24 (12), 10094. 10.3390/ijms241210094 37373242 PMC10298906

[B130] Gomez-Virgilio L. Silva-Lucero M. D. Flores-Morelos D. S. Gallardo-Nieto J. Lopez-Toledo G. Abarca-Fernandez A. M. (2022). Autophagy: a key regulator of homeostasis and disease: an overview of molecular mechanisms and modulators. Cells 11 (15), 2262. 10.3390/cells11152262 35892559 PMC9329718

[B131] Gong L. Huang D. Shi Y. Liang Z. Bu H. (2023). Regulated cell death in cancer: from pathogenesis to treatment. Chin. Med. J. Engl. 136 (6), 653–665. 10.1097/CM9.0000000000002239 35950752 PMC10129203

[B132] Gong Y. Fan Z. Luo G. Yang C. Huang Q. Fan K. (2019). The role of necroptosis in cancer biology and therapy. Mol. Cancer 18 (1), 100. 10.1186/s12943-019-1029-8 31122251 PMC6532150

[B133] Gordy C. He Y. W. (2012). The crosstalk between autophagy and apoptosis: where does this lead? Protein Cell 3 (1), 17–27. 10.1007/s13238-011-1127-x 22314807 PMC4875212

[B134] Goy A. Hernandez-Ilzaliturri F. J. Kahl B. Ford P. Protomastro E. Berger M. (2014). A phase I/II study of the pan Bcl-2 inhibitor obatoclax mesylate plus bortezomib for relapsed or refractory mantle cell lymphoma. Leuk. Lymphoma 55 (12), 2761–2768. 10.3109/10428194.2014.907891 24679008 PMC4349217

[B135] Greco F. A. Bonomi P. Crawford J. Kelly K. Oh Y. Halpern W. (2008). Phase 2 study of mapatumumab, a fully human agonistic monoclonal antibody which targets and activates the TRAIL receptor-1, in patients with advanced non-small cell lung cancer. Lung Cancer 61 (1), 82–90. 10.1016/j.lungcan.2007.12.011 18255187

[B136] Gu L. Zhang H. Liu T. Draganov A. Yi S. Wang B. (2018). Inhibition of MDM2 by a rhein-derived compound AQ-101 suppresses cancer development in SCID mice. Mol. Cancer Ther. 17 (2), 497–507. 10.1158/1535-7163.MCT-17-0566 29282301 PMC6054458

[B137] Guo J. Xu B. Han Q. Zhou H. Xia Y. Gong C. (2018). Ferroptosis: a novel anti-tumor action for cisplatin. Cancer Res. Treat. 50 (2), 445–460. 10.4143/crt.2016.572 28494534 PMC5912137

[B138] Hadian K. Stockwell B. R. (2023). The therapeutic potential of targeting regulated non-apoptotic cell death. Nat. Rev. Drug Discov. 22 (9), 723–742. 10.1038/s41573-023-00749-8 37550363

[B139] Halliwell B. Chirico S. (1993). Lipid peroxidation: its mechanism, measurement, and significance. Am. J. Clin. Nutr. 57 (5 Suppl. l), 715S–725S. 10.1093/ajcn/57.5.715S 8475889

[B140] Han Q. Ma Y. Wang H. Dai Y. Chen C. Liu Y. (2018). Resibufogenin suppresses colorectal cancer growth and metastasis through RIP3-mediated necroptosis. J. Transl. Med. 16 (1), 201. 10.1186/s12967-018-1580-x 30029665 PMC6053767

[B141] Han W. Li L. Qiu S. Lu Q. Pan Q. Gu Y. (2007). Shikonin circumvents cancer drug resistance by induction of a necroptotic death. Mol. Cancer Ther. 6 (5), 1641–1649. 10.1158/1535-7163.MCT-06-0511 17513612

[B142] Hangauer M. J. Viswanathan V. S. Ryan M. J. Bole D. Eaton J. K. Matov A. (2017). Drug-tolerant persister cancer cells are vulnerable to GPX4 inhibition. Nature 551 (7679), 247–250. 10.1038/nature24297 29088702 PMC5933935

[B143] Harraz M. M. Dawson T. M. Dawson V. L. (2008). Advances in neuronal cell death 2007. Stroke 39 (2), 286–288. 10.1161/STROKEAHA.107.511857 18187674

[B144] Harshman L. C. Kroeger N. Rha S. Y. Donskov F. Wood L. Tantravahi S. K. (2014). First-line Mammalian target of rapamycin inhibition in metastatic renal cell carcinoma: an analysis of practice patterns from the International Metastatic Renal Cell Carcinoma Database Consortium. Clin. Genitourin. Cancer 12 (5), 335–340. 10.1016/j.clgc.2014.03.003 24787966 PMC4164603

[B145] He S. Wang L. Miao L. Wang T. Du F. Zhao L. (2009). Receptor interacting protein kinase-3 determines cellular necrotic response to TNF-alpha. Cell 137 (6), 1100–1111. 10.1016/j.cell.2009.05.021 19524512

[B146] Heiss B. L. Chang E. Gao X. Truong T. Brave M. H. Bloomquist E. (2024). US food and drug administration approval summary: talazoparib in combination with enzalutamide for treatment of patients with homologous recombination repair gene-mutated metastatic castration-resistant prostate cancer. J. Clin. Oncol. 42 (15), 1851–1860. 10.1200/JCO.23.02182 38452327 PMC11095902

[B147] Herbst R. S. Kurzrock R. Hong D. S. Valdivieso M. Hsu C. P. Goyal L. (2010). A first-in-human study of conatumumab in adult patients with advanced solid tumors. Clin. Cancer Res. 16 (23), 5883–5891. 10.1158/1078-0432.CCR-10-0631 20947515

[B148] Hergueta-Redondo M. Sarrio D. Molina-Crespo A. Vicario R. Bernado-Morales C. Martinez L. (2016). Gasdermin B expression predicts poor clinical outcome in HER2-positive breast cancer. Oncotarget 7 (35), 56295–56308. 10.18632/oncotarget.10787 27462779 PMC5302915

[B149] Hitomi J. Christofferson D. E. Ng A. Yao J. Degterev A. Xavier R. J. (2008). Identification of a molecular signaling network that regulates a cellular necrotic cell death pathway. Cell 135 (7), 1311–1323. 10.1016/j.cell.2008.10.044 19109899 PMC2621059

[B150] Hotte S. J. Hirte H. W. Chen E. X. Siu L. L. Le L. H. Corey A. (2008). A phase 1 study of mapatumumab (fully human monoclonal antibody to TRAIL-R1) in patients with advanced solid malignancies. Clin. Cancer Res. 14 (11), 3450–3455. 10.1158/1078-0432.CCR-07-1416 18519776

[B151] Hu Z. Cai M. Zhang Y. Tao L. Guo R. (2020). miR-29c-3p inhibits autophagy and cisplatin resistance in ovarian cancer by regulating FOXP1/ATG14 pathway. Cell Cycle 19 (2), 193–206. 10.1080/15384101.2019.1704537 31885310 PMC6961660

[B152] Hua L. Zhu G. Wei J. (2018). MicroRNA-1 overexpression increases chemosensitivity of non-small cell lung cancer cells by inhibiting autophagy related 3-mediated autophagy. Cell Biol. Int. 42 (9), 1240–1249. 10.1002/cbin.10995 29851226

[B153] Hua Y. Zheng Y. Yao Y. Jia R. Ge S. Zhuang A. (2023). Metformin and cancer hallmarks: shedding new lights on therapeutic repurposing. J. Transl. Med. 21 (1), 403. 10.1186/s12967-023-04263-8 37344841 PMC10286395

[B154] Huang C. Y. Yu L. C. (2015). Pathophysiological mechanisms of death resistance in colorectal carcinoma. World J. Gastroenterol. 21 (41), 11777–11792. 10.3748/wjg.v21.i41.11777 26557002 PMC4631976

[B155] Huang K. J. Wei Y. H. Chiu Y. C. Wu S. R. Shieh D. B. (2019a). Assessment of zero-valent iron-based nanotherapeutics for ferroptosis induction and resensitization strategy in cancer cells. Biomater. Sci. 7 (4), 1311–1322. 10.1039/c8bm01525b 30734774

[B156] Huang P. Chen G. Jin W. Mao K. Wan H. He Y. (2022). Molecular mechanisms of parthanatos and its role in diverse diseases. Int. J. Mol. Sci. 23 (13), 7292. 10.3390/ijms23137292 35806303 PMC9266317

[B157] Huang Y. F. Kuo M. T. Liu Y. S. Cheng Y. M. Wu P. Y. Chou C. Y. (2019b). A dose escalation study of trientine plus carboplatin and pegylated liposomal doxorubicin in women with a first relapse of epithelial ovarian, tubal, and peritoneal cancer within 12 Months after platinum-based chemotherapy. Front. Oncol. 9, 437. 10.3389/fonc.2019.00437 31179244 PMC6544081

[B158] Hymowitz S. G. Christinger H. W. Fuh G. Ultsch M. O'Connell M. Kelley R. F. (1999). Triggering cell death: the crystal structure of Apo2L/TRAIL in a complex with death receptor 5. Mol. Cell 4 (4), 563–571. 10.1016/s1097-2765(00)80207-5 10549288

[B159] Ishaq M. Khan M. A. Sharma K. Sharma G. Dutta R. K. Majumdar S. (2014). Gambogic acid induced oxidative stress dependent caspase activation regulates both apoptosis and autophagy by targeting various key molecules (NF-κB, Beclin-1, p62 and NBR1) in human bladder cancer cells. Biochim. Biophys. Acta 1840 (12), 3374–3384. 10.1016/j.bbagen.2014.08.019 25218692

[B160] Iurlaro R. Munoz-Pinedo C. (2016). Cell death induced by endoplasmic reticulum stress. FEBS J. 283 (14), 2640–2652. 10.1111/febs.13598 26587781

[B161] Jan R. Chaudhry G. E. (2019). Understanding apoptosis and apoptotic pathways targeted cancer therapeutics. Adv. Pharm. Bull. 9 (2), 205–218. 10.15171/apb.2019.024 31380246 PMC6664112

[B162] Jeon S. M. Shin E. A. (2018). Exploring vitamin D metabolism and function in cancer. Exp. Mol. Med. 50 (4), 20–14. 10.1038/s12276-018-0038-9 29657326 PMC5938036

[B163] Ji Y. Dai F. Zhou B. (2018). Designing salicylaldehyde isonicotinoyl hydrazones as Cu(II) ionophores with tunable chelation and release of copper for hitting redox Achilles heel of cancer cells. Free Radic. Biol. Med. 129, 215–226. 10.1016/j.freeradbiomed.2018.09.017 30240704

[B164] Jia Y. Wang X. Deng Y. Li S. Xu X. Qin Y. (2023). Pyroptosis provides new strategies for the treatment of cancer. J. Cancer 14 (1), 140–151. 10.7150/jca.77965 36605484 PMC9809330

[B165] Jiang Y. Shen X. Zhi F. Wen Z. Gao Y. Xu J. (2023). An overview of arsenic trioxide-involved combined treatment algorithms for leukemia: basic concepts and clinical implications. Cell Death Discov. 9 (1), 266. 10.1038/s41420-023-01558-z 37500645 PMC10374529

[B166] Johnson D. C. Taabazuing C. Y. Okondo M. C. Chui A. J. Rao S. D. Brown F. C. (2018). DPP8/DPP9 inhibitor-induced pyroptosis for treatment of acute myeloid leukemia. Nat. Med. 24 (8), 1151–1156. 10.1038/s41591-018-0082-y 29967349 PMC6082709

[B167] Joly F. Fabbro M. Follana P. Lequesne J. Medioni J. Lesoin A. (2022). A phase II study of Navitoclax (ABT-263) as single agent in women heavily pretreated for recurrent epithelial ovarian cancer: the MONAVI - GINECO study. Gynecol. Oncol. 165 (1), 30–39. 10.1016/j.ygyno.2022.01.021 35123771

[B168] Kaczmarek A. Vandenabeele P. Krysko D. V. (2013). Necroptosis: the release of damage-associated molecular patterns and its physiological relevance. Immunity 38 (2), 209–223. 10.1016/j.immuni.2013.02.003 23438821

[B169] Kagan V. E. Mao G. Qu F. Angeli J. P. Doll S. Croix C. S. (2017). Oxidized arachidonic and adrenic PEs navigate cells to ferroptosis. Nat. Chem. Biol. 13 (1), 81–90. 10.1038/nchembio.2238 27842066 PMC5506843

[B170] Kaiser W. J. Sridharan H. Huang C. Mandal P. Upton J. W. Gough P. J. (2013). Toll-like receptor 3-mediated necrosis via TRIF, RIP3, and MLKL. J. Biol. Chem. 288 (43), 31268–31279. 10.1074/jbc.M113.462341 24019532 PMC3829437

[B171] Kamgar-Dayhoff P. Brelidze T. I. (2021). Multifaceted effect of chlorpromazine in cancer: implications for cancer treatment. Oncotarget 12 (14), 1406–1426. 10.18632/oncotarget.28010 34262651 PMC8274723

[B172] Kang Z. Chen J. J. Yu Y. Li B. Sun S. Y. Zhang B. (2011). Drozitumab, a human antibody to death receptor 5, has potent antitumor activity against rhabdomyosarcoma with the expression of caspase-8 predictive of response. Clin. Cancer Res. 17 (10), 3181–3192. 10.1158/1078-0432.CCR-10-2874 21385927 PMC3096734

[B173] Karki R. Kanneganti T. D. (2021). The 'cytokine storm': molecular mechanisms and therapeutic prospects. Trends Immunol. 42 (8), 681–705. 10.1016/j.it.2021.06.001 34217595 PMC9310545

[B174] Kashbour M. Alhadeethi A. Awwad S. Yassin M. Amin A. Abed M. (2024). The efficacy of Veliparib in combination with chemotherapy in the treatment of lung cancer: systematic review and meta-analysis. Expert Rev. Anticancer Ther., 1–11. 10.1080/14737140.2024.2417770 39428643

[B175] Kasof G. M. Prosser J. C. Liu D. Lorenzi M. V. Gomes B. C. (2000). The RIP-like kinase, RIP3, induces apoptosis and NF-kappaB nuclear translocation and localizes to mitochondria. FEBS Lett. 473 (3), 285–291. 10.1016/s0014-5793(00)01473-3 10818227

[B176] Kasznicki J. Sliwinska A. Drzewoski J. (2014). Metformin in cancer prevention and therapy. Ann. Transl. Med. 2 (6), 57. 10.3978/j.issn.2305-5839.2014.06.01 25333032 PMC4200668

[B177] Kawamoto Y. Yamai T. Ikezawa K. Seiki Y. Watsuji K. Hirao T. (2024). Clinical significance of germline breast cancer susceptibility gene (gBRCA) testing and olaparib as maintenance therapy for patients with pancreatic cancer. BMC Cancer 24 (1), 1000. 10.1186/s12885-024-12722-8 39134950 PMC11321060

[B178] Kayagaki N. Stowe I. B. Lee B. L. O'Rourke K. Anderson K. Warming S. (2015). Caspase-11 cleaves gasdermin D for non-canonical inflammasome signalling. Nature 526 (7575), 666–671. 10.1038/nature15541 26375259

[B179] Kayagaki N. Webster J. D. Newton K. (2024). Control of cell death in health and disease. Annu. Rev. Pathol. 19, 157–180. 10.1146/annurev-pathmechdis-051022-014433 37788577

[B180] Keldsen N. Havsteen H. Vergote I. Bertelsen K. Jakobsen A. (2003). Altretamine (hexamethylmelamine) in the treatment of platinum-resistant ovarian cancer: a phase II study. Gynecol. Oncol. 88 (2), 118–122. 10.1016/s0090-8258(02)00103-8 12586589

[B181] Kelley K. C. Grossman K. F. Brittain-Blankenship M. Thorne K. M. Akerley W. L. Terrazas M. C. (2021). A Phase 1 dose-escalation study of disulfiram and copper gluconate in patients with advanced solid tumors involving the liver using S-glutathionylation as a biomarker. BMC Cancer 21 (1), 510. 10.1186/s12885-021-08242-4 33957901 PMC8103752

[B182] Khan S. U. Fatima K. Aisha S. Malik F. (2024). Unveiling the mechanisms and challenges of cancer drug resistance. Cell Commun. Signal 22 (1), 109. 10.1186/s12964-023-01302-1 38347575 PMC10860306

[B183] Kim K. W. Hwang M. Moretti L. Jaboin J. J. Cha Y. I. Lu B. (2008). Autophagy upregulation by inhibitors of caspase-3 and mTOR enhances radiotherapy in a mouse model of lung cancer. Autophagy 4 (5), 659–668. 10.4161/auto.6058 18424912 PMC3073356

[B184] Kim S. R. Lewis J. M. Cyrenne B. M. Monico P. F. Mirza F. N. Carlson K. R. (2018). BET inhibition in advanced cutaneous T cell lymphoma is synergistically potentiated by BCL2 inhibition or HDAC inhibition. Oncotarget 9 (49), 29193–29207. 10.18632/oncotarget.25670 30018745 PMC6044378

[B185] Kindler H. L. Richards D. A. Garbo L. E. Garon E. B. Stephenson J. J. Jr. Rocha-Lima C. M. (2012). A randomized, placebo-controlled phase 2 study of ganitumab (AMG 479) or conatumumab (AMG 655) in combination with gemcitabine in patients with metastatic pancreatic cancer. Ann. Oncol. 23 (11), 2834–2842. 10.1093/annonc/mds142 22700995

[B186] Koch A. Jeiler B. Roedig J. van Wijk S. J. L. Dolgikh N. Fulda S. (2021). Smac mimetics and TRAIL cooperate to induce MLKL-dependent necroptosis in Burkitt's lymphoma cell lines. Neoplasia 23 (5), 539–550. 10.1016/j.neo.2021.03.003 33971465 PMC8122156

[B187] Kona S. V. Kalivendi S. V. (2024). The USP10/13 inhibitor, spautin-1, attenuates the progression of glioblastoma by independently regulating RAF-ERK mediated glycolysis and SKP2. Biochim. Biophys. Acta Mol. Basis Dis. 1870 (7), 167291. 10.1016/j.bbadis.2024.167291 38857836

[B188] Koo G. B. Morgan M. J. Lee D. G. Kim W. J. Yoon J. H. Koo J. S. (2015). Methylation-dependent loss of RIP3 expression in cancer represses programmed necrosis in response to chemotherapeutics. Cell Res. 25 (6), 707–725. 10.1038/cr.2015.56 25952668 PMC4456623

[B189] Koren E. Fuchs Y. (2021). Modes of regulated cell death in cancer. Cancer Discov. 11 (2), 245–265. 10.1158/2159-8290.CD-20-0789 33462123

[B190] Kou L. Xie X. Chen X. Li B. Li J. Li Y. (2023). The progress of research on immune checkpoint inhibitor resistance and reversal strategies for hepatocellular carcinoma. Cancer Immunol. Immunother. 72 (12), 3953–3969. 10.1007/s00262-023-03568-3 37917364 PMC10992589

[B191] Krutilina R. I. Hartman K. L. Oluwalana D. Playa H. C. Parke D. N. Chen H. (2022). Sabizabulin, a potent orally bioavailable colchicine binding site agent, suppresses HER2+ breast cancer and metastasis. Cancers (Basel) 14 (21), 5336. 10.3390/cancers14215336 36358755 PMC9658816

[B192] Kuwahara Y. Oikawa T. Ochiai Y. Roudkenar M. H. Fukumoto M. Shimura T. (2011). Enhancement of autophagy is a potential modality for tumors refractory to radiotherapy. Cell Death Dis. 2 (6), e177. 10.1038/cddis.2011.56 21716292 PMC3168998

[B193] Lage H. Helmbach H. Grottke C. Dietel M. Schadendorf D. (2001). DFNA5 (ICERE-1) contributes to acquired etoposide resistance in melanoma cells. FEBS Lett. 494 (1-2), 54–59. 10.1016/s0014-5793(01)02304-3 11297734

[B194] Lamichhane P. P. Samir P. (2023). Cellular stress: modulator of regulated cell death. Biol. (Basel) 12 (9), 1172. 10.3390/biology12091172 PMC10525759 37759572

[B195] Lang X. Green M. D. Wang W. Yu J. Choi J. E. Jiang L. (2019). Radiotherapy and immunotherapy promote tumoral lipid oxidation and ferroptosis via synergistic repression of SLC7A11. Cancer Discov. 9 (12), 1673–1685. 10.1158/2159-8290.CD-19-0338 31554642 PMC6891128

[B196] Lara P. N. Jr. Villanueva L. Ibanez C. Erman M. Lee J. L. Heinrich D. (2024). A randomized, open-label, phase 3 trial of pembrolizumab plus epacadostat versus sunitinib or pazopanib as first-line treatment for metastatic renal cell carcinoma (KEYNOTE-679/ECHO-302). BMC Cancer 23 (Suppl. 1), 1253. 10.1186/s12885-023-10971-7 39054430 PMC11270760

[B197] LeBlanc H. N. Ashkenazi A. (2003). Apo2L/TRAIL and its death and decoy receptors. Cell Death Differ. 10 (1), 66–75. 10.1038/sj.cdd.4401187 12655296

[B198] Lee H. O. Mustafa A. Hudes G. R. Kruger W. D. (2015). Hydroxychloroquine destabilizes phospho-S6 in human renal carcinoma cells. PLoS One 10 (7), e0131464. 10.1371/journal.pone.0131464 26134285 PMC4489871

[B199] Lee J. M. Brady M. F. Miller A. Moore R. G. MacKay H. McNally L. (2024). Cediranib and olaparib combination compared with cediranib or olaparib alone, or chemotherapy in platinum-resistant or primary platinum-refractory ovarian cancer: NRG-GY005. J. Clin. Oncol., JCO2400683. 10.1200/JCO.24.00683 39361946 PMC11652233

[B200] Lee J. M. Kim H. S. Kim A. Chang Y. S. Lee J. G. Cho J. (2022). ABT-737, a BH3 mimetic, enhances the therapeutic effects of ionizing radiation in K-ras mutant non-small cell lung cancer preclinical model. Yonsei Med. J. 63 (1), 16–25. 10.3349/ymj.2022.63.1.16 34913280 PMC8688371

[B201] Lee S. Y. Seo J. H. Kim S. Hwang C. Jeong D. I. Park J. (2023). Cuproptosis-inducible chemotherapeutic/cascade catalytic reactor system for combating with breast cancer. Small 19 (35), e2301402. 10.1002/smll.202301402 37162448

[B202] Lehmann S. Bykov V. J. Ali D. Andren O. Cherif H. Tidefelt U. (2012). Targeting p53 *in vivo*: a first-in-human study with p53-targeting compound APR-246 in refractory hematologic malignancies and prostate cancer. J. Clin. Oncol. 30 (29), 3633–3639. 10.1200/JCO.2011.40.7783 22965953

[B203] Lei G. Zhang Y. Koppula P. Liu X. Zhang J. Lin S. H. (2020). The role of ferroptosis in ionizing radiation-induced cell death and tumor suppression. Cell Res. 30 (2), 146–162. 10.1038/s41422-019-0263-3 31949285 PMC7015061

[B204] Lei G. Zhuang L. Gan B. (2024). The roles of ferroptosis in cancer: tumor suppression, tumor microenvironment, and therapeutic interventions. Cancer Cell 42 (4), 513–534. 10.1016/j.ccell.2024.03.011 38593779

[B205] Levantini E. (2023). Novel therapeutic targets in cancers. Int. J. Mol. Sci. 24 (19), 14660. 10.3390/ijms241914660 37834107 PMC10572778

[B206] Levine A. J. (2022). Targeting the P53 protein for cancer therapies: the translational impact of P53 research. Cancer Res. 82 (3), 362–364. 10.1158/0008-5472.CAN-21-2709 35110395 PMC8852246

[B207] Li C. Zhang J. Pan P. Zhang J. Hou X. Wang Y. (2024a). Humanistic health management and cancer: associations of psychology, nutrition, and exercise with cancer progression and pathogenesis. Adv. Sci. (Weinh) 11 (22), e2400665. 10.1002/advs.202400665 38526194 PMC11165509

[B208] Li H. Liu W. Zhang X. Wu F. Sun D. Wang Z. (2021). Ketamine suppresses proliferation and induces ferroptosis and apoptosis of breast cancer cells by targeting KAT5/GPX4 axis. Biochem. Biophys. Res. Commun. 585, 111–116. 10.1016/j.bbrc.2021.11.029 34800882

[B209] Li J. Chen S. Liao Y. Wang H. Zhou D. Zhang B. (2022a). Arecoline is associated with inhibition of cuproptosis and proliferation of cancer-associated fibroblasts in oral squamous cell carcinoma: a potential mechanism for tumor metastasis. Front. Oncol. 12, 925743. 10.3389/fonc.2022.925743 35875097 PMC9303015

[B210] Li K. Tan L. Li Y. Lyu Y. Zheng X. Jiang H. (2022b). Cuproptosis identifies respiratory subtype of renal cancer that confers favorable prognosis. Apoptosis 27 (11-12), 1004–1014. 10.1007/s10495-022-01769-2 36103026

[B211] Li L. G. Peng X. C. Yang Z. Y. Han N. Gou C. L. Shi J. (2024b). Dihydroartemisinin-driven selective anti-lung cancer proliferation by binding to EGFR and inhibition of NRAS signaling pathway-induced DNA damage. Sci. Rep. 14 (1), 11704. 10.1038/s41598-024-62126-8 38778121 PMC11111767

[B212] Li Q. Lv D. Sun X. Wang M. Cai L. Liu F. (2024c). Inetetamab combined with sirolimus and chemotherapy for the treatment of HER2-positive metastatic breast cancer patients with abnormal activation of the PI3K/Akt/mTOR pathway after trastuzumab treatment. Cancer Innov. 3 (5), e145. 10.1002/cai2.145 39301201 PMC11411696

[B213] Li R. Ding C. Zhang J. Xie M. Park D. Ding Y. (2017). Modulation of Bax and mTOR for cancer therapeutics. Cancer Res. 77 (11), 3001–3012. 10.1158/0008-5472.CAN-16-2356 28381544 PMC5503158

[B214] Li X. Zhou Y. Li Y. Yang L. Ma Y. Peng X. (2019). Autophagy: a novel mechanism of chemoresistance in cancers. Biomed. Pharmacother. 119, 109415. 10.1016/j.biopha.2019.109415 31514065

[B215] Liao M. Qin R. Huang W. Zhu H. P. Peng F. Han B. (2022a). Targeting regulated cell death (RCD) with small-molecule compounds in triple-negative breast cancer: a revisited perspective from molecular mechanisms to targeted therapies. J. Hematol. Oncol. 15 (1), 44. 10.1186/s13045-022-01260-0 35414025 PMC9006445

[B216] Liao P. Wang W. Wang W. Kryczek I. Li X. Bian Y. (2022b). CD8(+) T cells and fatty acids orchestrate tumor ferroptosis and immunity via ACSL4. Cancer Cell 40 (4), 365–378.e6. 10.1016/j.ccell.2022.02.003 35216678 PMC9007863

[B217] Lin S. Q. Jia F. J. Zhang C. Y. Liu F. Y. Ma J. H. Han Z. (2019a). Actinomycin V suppresses human non-small-cell lung carcinoma A549 cells by inducing G2/M phase arrest and apoptosis via the p53-dependent pathway. Mar. Drugs 17 (10), 572. 10.3390/md17100572 31601054 PMC6835885

[B218] Lin X. Jia Y. Dong X. Shen J. Jin Y. Li Y. (2019b). Diplatin, a novel and low-toxicity anti-lung cancer platinum complex, activation of cell death in tumors via a ROS/JNK/p53-Dependent pathway, and a low rate of acquired treatment resistance. Front. Pharmacol. 10, 982. 10.3389/fphar.2019.00982 31572176 PMC6749073

[B219] Lindemann A. Patel A. A. Silver N. L. Tang L. Liu Z. Wang L. (2019). COTI-2, A novel thiosemicarbazone derivative, exhibits antitumor activity in HNSCC through p53-dependent and -independent mechanisms. Clin. Cancer Res. 25 (18), 5650–5662. 10.1158/1078-0432.CCR-19-0096 31308060 PMC6759991

[B220] Liu B. Zhou H. Tan L. Siu K. T. H. Guan X. Y. (2024a). Exploring treatment options in cancer: tumor treatment strategies. Signal Transduct. Target Ther. 9 (1), 175. 10.1038/s41392-024-01856-7 39013849 PMC11252281

[B221] Liu J. Kuang F. Kroemer G. Klionsky D. J. Kang R. Tang D. (2020a). Autophagy-dependent ferroptosis: machinery and regulation. Cell Chem. Biol. 27 (4), 420–435. 10.1016/j.chembiol.2020.02.005 32160513 PMC7166192

[B222] Liu J. Yuan Y. Cheng Y. Fu D. Chen Z. Wang Y. (2022). Copper-based metal-organic framework overcomes cancer chemoresistance through systemically disrupting dynamically balanced cellular redox homeostasis. J. Am. Chem. Soc. 144 (11), 4799–4809. 10.1021/jacs.1c11856 35192770

[B223] Liu S. Yao S. Yang H. Liu S. Wang Y. (2023). Autophagy: regulator of cell death. Cell Death Dis. 14 (10), 648. 10.1038/s41419-023-06154-8 37794028 PMC10551038

[B224] Liu T. Sun X. Cao Z. (2019). Shikonin-induced necroptosis in nasopharyngeal carcinoma cells via ROS overproduction and upregulation of RIPK1/RIPK3/MLKL expression. Onco Targets Ther. 12, 2605–2614. 10.2147/OTT.S200740 31118661 PMC6498394

[B225] Liu X. Zhou M. Mei L. Ruan J. Hu Q. Peng J. (2016). Key roles of necroptotic factors in promoting tumor growth. Oncotarget 7 (16), 22219–22233. 10.18632/oncotarget.7924 26959742 PMC5008357

[B226] Liu Y. Fang Y. Chen X. Wang Z. Liang X. Zhang T. (2020b). Gasdermin E-mediated target cell pyroptosis by CAR T cells triggers cytokine release syndrome. Sci. Immunol. 5 (43), eaax7969. 10.1126/sciimmunol.aax7969 31953257

[B227] Liu Y. Mondello P. Erazo T. Tannan N. B. Asgari Z. de Stanchina E. (2018). NOXA genetic amplification or pharmacologic induction primes lymphoma cells to BCL2 inhibitor-induced cell death. Proc. Natl. Acad. Sci. U. S. A. 115 (47), 12034–12039. 10.1073/pnas.1806928115 30404918 PMC6255185

[B228] Liu Y. Pan R. Ouyang Y. Gu W. Xiao T. Yang H. (2024b). Pyroptosis in health and disease: mechanisms, regulation and clinical perspective. Signal Transduct. Target Ther. 9 (1), 245. 10.1038/s41392-024-01958-2 39300122 PMC11413206

[B229] Liu Z. Y. Wu B. Guo Y. S. Zhou Y. H. Fu Z. G. Xu B. Q. (2015). Necrostatin-1 reduces intestinal inflammation and colitis-associated tumorigenesis in mice. Am. J. Cancer Res. 5 (10), 3174–3185. 26693068 PMC4656739

[B230] Llovet J. M. Ricci S. Mazzaferro V. Hilgard P. Gane E. Blanc J. F. (2008). Sorafenib in advanced hepatocellular carcinoma. N. Engl. J. Med. 359 (4), 378–390. 10.1056/NEJMoa0708857 18650514

[B231] Loi S. Karapetis C. S. McCarthy N. Oakman C. Redfern A. White M. (2022). Palbociclib plus letrozole as treatment for postmenopausal women with hormone receptor-positive/human epidermal growth factor receptor 2-negative advanced breast cancer for whom letrozole therapy is deemed appropriate: an expanded access study in Australia and India. Asia Pac J. Clin. Oncol. 18 (6), 560–569. 10.1111/ajco.13653 34908235 PMC9787838

[B232] Lowe S. W. Lin A. W. (2000). Apoptosis in cancer. Carcinogenesis 21 (3), 485–495. 10.1093/carcin/21.3.485 10688869

[B233] Lu W. Cheng F. Yan W. Li X. Yao X. Song W. (2017). Selective targeting p53(WT) lung cancer cells harboring homozygous p53 Arg72 by an inhibitor of CypA. Oncogene 36 (33), 4719–4731. 10.1038/onc.2017.41 28394340 PMC5562848

[B234] Lu X. Chen L. Chen Y. Shao Q. Qin W. (2015). Bafilomycin A1 inhibits the growth and metastatic potential of the BEL-7402 liver cancer and HO-8910 ovarian cancer cell lines and induces alterations in their microRNA expression. Exp. Ther. Med. 10 (5), 1829–1834. 10.3892/etm.2015.2758 26640557 PMC4665926

[B235] Lu Z. Wu C. Zhu M. Song W. Wang H. Wang J. (2020). Ophiopogonin D' induces RIPK1-dependent necroptosis in androgen-dependent LNCaP prostate cancer cells. Int. J. Oncol. 56 (2), 439–447. 10.3892/ijo.2019.4945 31894265 PMC6959467

[B236] Luo Y. Bai X. Y. Zhang L. Hu Q. Q. Zhang N. Cheng J. Z. (2024). Ferroptosis in cancer therapy: mechanisms, small molecule inducers, and novel approaches. Drug Des. Devel Ther. 18, 2485–2529. 10.2147/DDDT.S472178 PMC11198730 38919962

[B237] Ma D. Lu B. Feng C. Wang C. Wang Y. Luo T. (2016a). Deoxypodophyllotoxin triggers parthanatos in glioma cells via induction of excessive ROS. Cancer Lett. 371 (2), 194–204. 10.1016/j.canlet.2015.11.044 26683770

[B238] Ma J. Li L. Yue K. Li Y. Liu H. Wang P. G. (2020). Bromocoumarinplatin, targeting simultaneously mitochondria and nuclei with p53 apoptosis pathway to overcome cisplatin resistance. Bioorg Chem. 99, 103768. 10.1016/j.bioorg.2020.103768 32217375

[B239] Ma S. Henson E. S. Chen Y. Gibson S. B. (2016b). Ferroptosis is induced following siramesine and lapatinib treatment of breast cancer cells. Cell Death Dis. 7 (7), e2307. 10.1038/cddis.2016.208 27441659 PMC4973350

[B240] Ma S. Zhu J. Wang M. Zhu J. Wang W. Xiong Y. (2022). A cuproptosis-related long non-coding RNA signature to predict the prognosis and immune microenvironment characterization for lung adenocarcinoma. Transl. Lung Cancer Res. 11 (10), 2079–2093. 10.21037/tlcr-22-660 36386454 PMC9641048

[B241] Ma X. Xiao L. Liu L. Ye L. Su P. Bi E. (2021). CD36-mediated ferroptosis dampens intratumoral CD8(+) T cell effector function and impairs their antitumor ability. Cell Metab. 33 (5), 1001–1012.e5. 10.1016/j.cmet.2021.02.015 33691090 PMC8102368

[B242] Ma Z. G. Ma R. Xiao X. L. Zhang Y. H. Zhang X. Z. Hu N. (2016c). Azo polymeric micelles designed for colon-targeted dimethyl fumarate delivery for colon cancer therapy. Acta Biomater. 44, 323–331. 10.1016/j.actbio.2016.08.021 27544813

[B243] MacDonald G. Nalvarte I. Smirnova T. Vecchi M. Aceto N. Dolemeyer A. (2014). Memo is a copper-dependent redox protein with an essential role in migration and metastasis. Sci. Signal 7 (329), ra56. 10.1126/scisignal.2004870 24917593

[B244] Mahalingam D. Mita M. Sarantopoulos J. Wood L. Amaravadi R. K. Davis L. E. (2014). Combined autophagy and HDAC inhibition: a phase I safety, tolerability, pharmacokinetic, and pharmacodynamic analysis of hydroxychloroquine in combination with the HDAC inhibitor vorinostat in patients with advanced solid tumors. Autophagy 10 (8), 1403–1414. 10.4161/auto.29231 24991835 PMC4203517

[B245] Maleki Vareki S. Salim K. Y. Danter W. R. Koropatnick J. (2018). Novel anti-cancer drug COTI-2 synergizes with therapeutic agents and does not induce resistance or exhibit cross-resistance in human cancer cell lines. PLoS One 13 (1), e0191766. 10.1371/journal.pone.0191766 29364966 PMC5783418

[B246] Man S. M. Karki R. Kanneganti T. D. (2017). Molecular mechanisms and functions of pyroptosis, inflammatory caspases and inflammasomes in infectious diseases. Immunol. Rev. 277 (1), 61–75. 10.1111/imr.12534 28462526 PMC5416822

[B247] Mann B. S. Johnson J. R. Cohen M. H. Justice R. Pazdur R. (2007). FDA approval summary: vorinostat for treatment of advanced primary cutaneous T-cell lymphoma. Oncologist 12 (10), 1247–1252. 10.1634/theoncologist.12-10-1247 17962618

[B248] Markowski M. C. Tutrone R. Pieczonka C. Barnette K. G. Getzenberg R. H. Rodriguez D. (2022). A phase ib/II study of sabizabulin, a novel oral cytoskeleton disruptor, in men with metastatic castration-resistant prostate cancer with progression on an androgen receptor-targeting agent. Clin. Cancer Res. 28 (13), 2789–2795. 10.1158/1078-0432.CCR-22-0162 35416959 PMC9774054

[B249] Marsh J. W. Djoko K. Y. McEwan A. G. Huston W. M. (2017). Copper(II)-bis(thiosemicarbazonato) complexes as anti-chlamydial agents. Pathog. Dis. 75 (7). 10.1093/femspd/ftx084 28830076

[B250] Maru D. Hothi A. Bagariya C. Kumar A. (2022). Targeting ferroptosis pathways: a novel strategy for cancer therapy. Curr. Cancer Drug Targets 22 (3), 234–244. 10.2174/1568009622666220211122745 35152865

[B251] Masaldan S. Clatworthy S. A. S. Gamell C. Meggyesy P. M. Rigopoulos A. T. Haupt S. (2018). Iron accumulation in senescent cells is coupled with impaired ferritinophagy and inhibition of ferroptosis. Redox Biol. 14, 100–115. 10.1016/j.redox.2017.08.015 28888202 PMC5596264

[B252] Mason K. A. Valdecanas D. Hunter N. R. Milas L. (2008). INO-1001, a novel inhibitor of poly(ADP-ribose) polymerase, enhances tumor response to doxorubicin. Invest. New Drugs 26 (1), 1–5. 10.1007/s10637-007-9072-5 17628743

[B253] Mateo J. Moreno V. Gupta A. Kaye S. B. Dean E. Middleton M. R. (2016). An adaptive study to determine the optimal dose of the tablet formulation of the PARP inhibitor olaparib. Target Oncol. 11 (3), 401–415. 10.1007/s11523-016-0435-8 27169564

[B254] Matteoni S. Matarrese P. Ascione B. Buccarelli M. Ricci-Vitiani L. Pallini R. (2021). Anticancer properties of the antipsychotic drug chlorpromazine and its synergism with temozolomide in restraining human glioblastoma proliferation *in vitro* . Front. Oncol. 11, 635472. 10.3389/fonc.2021.635472 33718225 PMC7952964

[B255] McIlwain D. R. Berger T. Mak T. W. (2015). Caspase functions in cell death and disease. Cold Spring Harb. Perspect. Biol. 7 (4), a026716. 10.1101/cshperspect.a026716 25833847 PMC4382736

[B256] Meier P. Legrand A. J. Adam D. Silke J. (2024). Immunogenic cell death in cancer: targeting necroptosis to induce antitumour immunity. Nat. Rev. Cancer 24 (5), 299–315. 10.1038/s41568-024-00674-x 38454135

[B257] Mele L. Del Vecchio V. Liccardo D. Prisco C. Schwerdtfeger M. Robinson N. (2020). The role of autophagy in resistance to targeted therapies. Cancer Treat. Rev. 88, 102043. 10.1016/j.ctrv.2020.102043 32505806

[B258] Meng M. B. Wang H. H. Cui Y. L. Wu Z. Q. Shi Y. Y. Zaorsky N. G. (2016). Necroptosis in tumorigenesis, activation of anti-tumor immunity, and cancer therapy. Oncotarget 7 (35), 57391–57413. 10.18632/oncotarget.10548 27429198 PMC5302997

[B259] Michie J. Kearney C. J. Hawkins E. D. Silke J. Oliaro J. (2020). The immuno-modulatory effects of inhibitor of apoptosis protein antagonists in cancer immunotherapy. Cells 9 (1), 207. 10.3390/cells9010207 31947615 PMC7017284

[B260] Mishra R. Zokaei Nikoo M. Veeraballi S. Singh A. (2023). Venetoclax and hypomethylating agent combination in myeloid malignancies: mechanisms of synergy and challenges of resistance. Int. J. Mol. Sci. 25 (1), 484. 10.3390/ijms25010484 38203655 PMC10778677

[B261] Mizuno M. Ito K. Nakai H. Kato H. Kamiura S. Ushijima K. (2023). Veliparib with frontline chemotherapy and as maintenance in Japanese women with ovarian cancer: a subanalysis of efficacy, safety, and antiemetic use in the phase 3 VELIA trial. Int. J. Clin. Oncol. 28 (1), 163–174. 10.1007/s10147-022-02258-x 36534262 PMC9823063

[B262] Mohammad R. M. Muqbil I. Lowe L. Yedjou C. Hsu H. Y. Lin L. T. (2015). Broad targeting of resistance to apoptosis in cancer. Semin. Cancer Biol. 35, S78–S103. 10.1016/j.semcancer.2015.03.001 25936818 PMC4720504

[B263] Mohammadinejad R. Moosavi M. A. Tavakol S. Vardar D. O. Hosseini A. Rahmati M. (2019). Necrotic, apoptotic and autophagic cell fates triggered by nanoparticles. Autophagy 15 (1), 4–33. 10.1080/15548627.2018.1509171 30160607 PMC6287681

[B264] Molina-Crespo A. Cadete A. Sarrio D. Gamez-Chiachio M. Martinez L. Chao K. (2019). Intracellular delivery of an antibody targeting gasdermin-B reduces HER2 breast cancer aggressiveness. Clin. Cancer Res. 25 (15), 4846–4858. 10.1158/1078-0432.CCR-18-2381 31064780

[B265] Monian P. Jiang X. (2012). Clearing the final hurdles to mitochondrial apoptosis: regulation post cytochrome C release. Exp. Oncol. 34 (3), 185–191. 23070003

[B266] Monk B. J. Parkinson C. Lim M. C. O'Malley D. M. Oaknin A. Wilson M. K. (2022). A randomized, phase III trial to evaluate rucaparib monotherapy as maintenance treatment in patients with newly diagnosed ovarian cancer (ATHENA-MONO/GOG-3020/ENGOT-ov45). J. Clin. Oncol. 40 (34), 3952–3964. 10.1200/JCO.22.01003 35658487 PMC9746782

[B267] Morana O. Wood W. Gregory C. D. (2022). The apoptosis paradox in cancer. Int. J. Mol. Sci. 23 (3), 1328. 10.3390/ijms23031328 35163253 PMC8836235

[B268] Morgan M. J. Kim Y. S. (2022). Roles of RIPK3 in necroptosis, cell signaling, and disease. Exp. Mol. Med. 54 (10), 1695–1704. 10.1038/s12276-022-00868-z 36224345 PMC9636380

[B269] Motzer R. J. Escudier B. Oudard S. Hutson T. E. Porta C. Bracarda S. (2008). Efficacy of everolimus in advanced renal cell carcinoma: a double-blind, randomised, placebo-controlled phase III trial. Lancet 372 (9637), 449–456. 10.1016/S0140-6736(08)61039-9 18653228

[B270] Murphy J. M. (2020). The killer pseudokinase mixed lineage kinase domain-like protein (MLKL). Cold Spring Harb. Perspect. Biol. 12 (8), a036376. 10.1101/cshperspect.a036376 31712266 PMC7397827

[B271] Nagai M. Vo N. H. Shin Ogawa L. Chimmanamada D. Inoue T. Chu J. (2012). The oncology drug elesclomol selectively transports copper to the mitochondria to induce oxidative stress in cancer cells. Free Radic. Biol. Med. 52 (10), 2142–2150. 10.1016/j.freeradbiomed.2012.03.017 22542443

[B272] Nagourney A. J. Gipoor J. B. Evans S. S. D'Amora P. Duesberg M. S. Bernard P. J. (2023). Therapeutic targeting of P53: a comparative analysis of APR-246 and COTI-2 in human tumor primary culture 3-D explants. Genes (Basel) 14 (3), 747. 10.3390/genes14030747 36981018 PMC10048363

[B273] Najafov A. Chen H. Yuan J. (2017). Necroptosis and cancer. Trends Cancer 3 (4), 294–301. 10.1016/j.trecan.2017.03.002 28451648 PMC5402749

[B274] Narang A. Hage Chehade C. Ozay Z. I. Nordblad B. Swami U. Agarwal N. (2024). Talazoparib for the treatment of prostate cancer. Expert Opin. Pharmacother. 25 (13), 1717–1727. 10.1080/14656566.2024.2397002 39210559

[B275] Nawrocki S. T. Han Y. Visconte V. Przychodzen B. Espitia C. M. Phillips J. (2019). The novel autophagy inhibitor ROC-325 augments the antileukemic activity of azacitidine. Leukemia 33 (12), 2971–2974. 10.1038/s41375-019-0529-2 31358855 PMC7462348

[B276] Nawrocki S. T. Wang W. Carew J. S. (2020). Autophagy: new insights into its roles in cancer progression and drug resistance. Cancers (Basel) 12 (10), 3005. 10.3390/cancers12103005 33081217 PMC7602821

[B277] Negroni A. Colantoni E. Cucchiara S. Stronati L. (2020). Necroptosis in intestinal inflammation and cancer: new concepts and therapeutic perspectives. Biomolecules 10 (10), 1431. 10.3390/biom10101431 33050394 PMC7599789

[B278] Newton K. Dugger D. L. Wickliffe K. E. Kapoor N. de Almagro M. C. Vucic D. (2014). Activity of protein kinase RIPK3 determines whether cells die by necroptosis or apoptosis. Science 343 (6177), 1357–1360. 10.1126/science.1249361 24557836

[B279] Newton K. Strasser A. Kayagaki N. Dixit V. M. (2024). Cell death. Cell 187 (2), 235–256. 10.1016/j.cell.2023.11.044 38242081

[B280] Nie Z. Chen M. Gao Y. Huang D. Cao H. Peng Y. (2022). Ferroptosis and tumor drug resistance: current status and major challenges. Front. Pharmacol. 13, 879317. 10.3389/fphar.2022.879317 35668934 PMC9163417

[B281] Nishikawa T. Matsumoto K. Tamura K. Yoshida H. Imai Y. Miyasaka A. (2017). Phase 1 dose-escalation study of single-agent veliparib in Japanese patients with advanced solid tumors. Cancer Sci. 108 (9), 1834–1842. 10.1111/cas.13307 28665051 PMC5581522

[B282] Nor Hisam N. S. Ugusman A. Rajab N. F. Ahmad M. F. Fenech M. Liew S. L. (2021). Combination therapy of navitoclax with chemotherapeutic agents in solid tumors and blood cancer: a review of current evidence. Pharmaceutics 13 (9), 1353. 10.3390/pharmaceutics13091353 34575429 PMC8468743

[B283] O'Day S. J. Eggermont A. M. Chiarion-Sileni V. Kefford R. Grob J. J. Mortier L. (2013). Final results of phase III SYMMETRY study: randomized, double-blind trial of elesclomol plus paclitaxel versus paclitaxel alone as treatment for chemotherapy-naive patients with advanced melanoma. J. Clin. Oncol. 31 (9), 1211–1218. 10.1200/JCO.2012.44.5585 23401447

[B284] Ojha R. Jha V. Singh S. K. (2016). Gemcitabine and mitomycin induced autophagy regulates cancer stem cell pool in urothelial carcinoma cells. Biochim. Biophys. Acta 1863 (2), 347–359. 10.1016/j.bbamcr.2015.12.002 26658162

[B285] Oliveri V. (2022). Selective targeting of cancer cells by copper ionophores: an overview. Front. Mol. Biosci. 9, 841814. 10.3389/fmolb.2022.841814 35309510 PMC8931543

[B286] Oliveri V. Lanza V. Milardi D. Viale M. Maric I. Sgarlata C. (2017). Amino- and chloro-8-hydroxyquinolines and their copper complexes as proteasome inhibitors and antiproliferative agents. Metallomics 9 (10), 1439–1446. 10.1039/c7mt00156h 28932850

[B287] Ozyerli-Goknar E. Bagci-Onder T. (2021). Epigenetic deregulation of apoptosis in cancers. Cancers (Basel) 13 (13), 3210. 10.3390/cancers13133210 34199020 PMC8267644

[B288] Paik P. K. Rudin C. M. Pietanza M. C. Brown A. Rizvi N. A. Takebe N. (2011). A phase II study of obatoclax mesylate, a Bcl-2 antagonist, plus topotecan in relapsed small cell lung cancer. Lung Cancer 74 (3), 481–485. 10.1016/j.lungcan.2011.05.005 21620511 PMC3715068

[B289] Parikh S. A. Kantarjian H. Schimmer A. Walsh W. Asatiani E. El-Shami K. (2010). Phase II study of obatoclax mesylate (GX15-070), a small-molecule BCL-2 family antagonist, for patients with myelofibrosis. Clin. Lymphoma Myeloma Leuk. 10 (4), 285–289. 10.3816/CLML.2010.n.059 20709666

[B290] Park E. J. Min K. J. Lee T. J. Yoo Y. H. Kim Y. S. Kwon T. K. (2014). β-Lapachone induces programmed necrosis through the RIP1-PARP-AIF-dependent pathway in human hepatocellular carcinoma SK-Hep1 cells. Cell Death Dis. 5 (5), e1230. 10.1038/cddis.2014.202 24832602 PMC4047891

[B291] Park J. M. Huang S. Wu T. T. Foster N. R. Sinicrope F. A. (2013). Prognostic impact of Beclin 1, p62/sequestosome 1 and LC3 protein expression in colon carcinomas from patients receiving 5-fluorouracil as adjuvant chemotherapy. Cancer Biol. Ther. 14 (2), 100–107. 10.4161/cbt.22954 23192274 PMC3571991

[B292] Parzych K. R. Klionsky D. J. (2014). An overview of autophagy: morphology, mechanism, and regulation. Antioxid. Redox Signal 20 (3), 460–473. 10.1089/ars.2013.5371 23725295 PMC3894687

[B293] Pasparakis M. Vandenabeele P. (2015). Necroptosis and its role in inflammation. Nature 517 (7534), 311–320. 10.1038/nature14191 25592536

[B294] Pasquier B. (2015). SAR405, a PIK3C3/Vps34 inhibitor that prevents autophagy and synergizes with MTOR inhibition in tumor cells. Autophagy 11 (4), 725–726. 10.1080/15548627.2015.1033601 25905679 PMC4502822

[B295] Pazzaglia S. Pioli C. (2019). Multifaceted role of PARP-1 in DNA repair and inflammation: pathological and therapeutic implications in cancer and non-cancer diseases. Cells 9 (1), 41. 10.3390/cells9010041 31877876 PMC7017201

[B296] Pellegrini P. Strambi A. Zipoli C. Hagg-Olofsson M. Buoncervello M. Linder S. (2014). Acidic extracellular pH neutralizes the autophagy-inhibiting activity of chloroquine: implications for cancer therapies. Autophagy 10 (4), 562–571. 10.4161/auto.27901 24492472 PMC3984580

[B297] Peng F. Liao M. Qin R. Zhu S. Peng C. Fu L. (2022). Regulated cell death (RCD) in cancer: key pathways and targeted therapies. Signal Transduct. Target Ther. 7 (1), 286. 10.1038/s41392-022-01110-y 35963853 PMC9376115

[B298] Petsri K. Chamni S. Suwanborirux K. Saito N. Chanvorachote P. (2019). Renieramycin T induces lung cancer cell apoptosis by targeting mcl-1 degradation: a new insight in the mechanism of action. Mar. Drugs 17 (5), 301. 10.3390/md17050301 31117253 PMC6562878

[B299] Pfeffer C. M. Singh A. T. K. (2018). Apoptosis: a target for anticancer therapy. Int. J. Mol. Sci. 19 (2), 448. 10.3390/ijms19020448 29393886 PMC5855670

[B300] Piccolo M. Ferraro M. G. Iazzetti F. Santamaria R. Irace C. (2024). Insight into iron, oxidative stress and ferroptosis: therapy targets for approaching anticancer strategies. Cancers (Basel) 16 (6), 1220. 10.3390/cancers16061220 38539554 PMC10969343

[B301] Piha-Paul S. A. Tseng C. Leung C. H. Yuan Y. Karp D. D. Subbiah V. (2024). Phase II study of talazoparib in advanced cancers with BRCA1/2, DNA repair, and PTEN alterations. NPJ Precis. Oncol. 8 (1), 166. 10.1038/s41698-024-00634-6 39085400 PMC11291882

[B302] Pizato N. Luzete B. C. Kiffer L. Correa L. H. de Oliveira Santos I. Assumpcao J. A. F. (2018). Omega-3 docosahexaenoic acid induces pyroptosis cell death in triple-negative breast cancer cells. Sci. Rep. 8 (1), 1952. 10.1038/s41598-018-20422-0 29386662 PMC5792438

[B303] Pu F. Chen F. Zhang Z. Shi D. Zhong B. Lv X. (2022). Ferroptosis as a novel form of regulated cell death: implications in the pathogenesis, oncometabolism and treatment of human cancer. Genes Dis. 9 (2), 347–357. 10.1016/j.gendis.2020.11.019 35224151 PMC8843993

[B304] Pukac L. Kanakaraj P. Humphreys R. Alderson R. Bloom M. Sung C. (2005). HGS-ETR1, a fully human TRAIL-receptor 1 monoclonal antibody, induces cell death in multiple tumour types *in vitro* and *in vivo* . Br. J. Cancer 92 (8), 1430–1441. 10.1038/sj.bjc.6602487 15846298 PMC2361994

[B305] Qi D. Peng M. (2023). Ferroptosis-mediated immune responses in cancer. Front. Immunol. 14, 1188365. 10.3389/fimmu.2023.1188365 37325669 PMC10264078

[B306] Qin K. Zhang F. Wang H. Wang N. Qiu H. Jia X. (2023). circRNA circSnx12 confers Cisplatin chemoresistance to ovarian cancer by inhibiting ferroptosis through a miR-194-5p/SLC7A11 axis. BMB Rep. 56 (2), 184–189. 10.5483/BMBRep.2022-0175 36617466 PMC10068343

[B307] Rangwala R. Chang Y. C. Hu J. Algazy K. M. Evans T. L. Fecher L. A. (2014a). Combined MTOR and autophagy inhibition: phase I trial of hydroxychloroquine and temsirolimus in patients with advanced solid tumors and melanoma. Autophagy 10 (8), 1391–1402. 10.4161/auto.29119 24991838 PMC4203516

[B308] Rangwala R. Leone R. Chang Y. C. Fecher L. A. Schuchter L. M. Kramer A. (2014b). Phase I trial of hydroxychloroquine with dose-intense temozolomide in patients with advanced solid tumors and melanoma. Autophagy 10 (8), 1369–1379. 10.4161/auto.29118 24991839 PMC4203514

[B309] Rao Z. Zhu Y. Yang P. Chen Z. Xia Y. Qiao C. (2022). Pyroptosis in inflammatory diseases and cancer. Theranostics 12 (9), 4310–4329. 10.7150/thno.71086 35673561 PMC9169370

[B310] Rashmi K. C. Harsha Raj M. Paul M. Girish K. S. Salimath B. P. Aparna H. S. (2019). A new pyrrole based small molecule from Tinospora cordifolia induces apoptosis in MDA-MB-231 breast cancer cells via ROS mediated mitochondrial damage and restoration of p53 activity. Chem. Biol. Interact. 299, 120–130. 10.1016/j.cbi.2018.12.005 30543781

[B311] Rebecca V. W. Nicastri M. C. Fennelly C. Chude C. I. Barber-Rotenberg J. S. Ronghe A. (2019). PPT1 promotes tumor growth and is the molecular target of chloroquine derivatives in cancer. Cancer Discov. 9 (2), 220–229. 10.1158/2159-8290.CD-18-0706 30442709 PMC6368875

[B312] Rebecca V. W. Nicastri M. C. McLaughlin N. Fennelly C. McAfee Q. Ronghe A. (2017). A unified approach to targeting the lysosome's degradative and growth signaling roles. Cancer Discov. 7 (11), 1266–1283. 10.1158/2159-8290.CD-17-0741 28899863 PMC5833978

[B313] Reck M. Schenker M. Lee K. H. Provencio M. Nishio M. Lesniewski-Kmak K. (2019). Nivolumab plus ipilimumab versus chemotherapy as first-line treatment in advanced non-small-cell lung cancer with high tumour mutational burden: patient-reported outcomes results from the randomised, open-label, phase III CheckMate 227 trial. Eur. J. Cancer 116, 137–147. 10.1016/j.ejca.2019.05.008 31195357

[B314] Redman B. G. Esper P. Pan Q. Dunn R. L. Hussain H. K. Chenevert T. (2003). Phase II trial of tetrathiomolybdate in patients with advanced kidney cancer. Clin. Cancer Res. 9 (5), 1666–1672. 12738719

[B315] Reed J. C. (2006). Drug insight: cancer therapy strategies based on restoration of endogenous cell death mechanisms. Nat. Clin. Pract. Oncol. 3 (7), 388–398. 10.1038/ncponc0538 16826219

[B316] Reeder N. L. Kaplan J. Xu J. Youngquist R. S. Wallace J. Hu P. (2011). Zinc pyrithione inhibits yeast growth through copper influx and inactivation of iron-sulfur proteins. Antimicrob. Agents Chemother. 55 (12), 5753–5760. 10.1128/AAC.00724-11 21947398 PMC3232817

[B317] Rocha Lima C. M. Bayraktar S. Flores A. M. MacIntyre J. Montero A. Baranda J. C. (2012). Phase Ib study of drozitumab combined with first-line mFOLFOX6 plus bevacizumab in patients with metastatic colorectal cancer. Cancer Invest. 30 (10), 727–731. 10.3109/07357907.2012.732163 23061802

[B318] Rodler E. Sharma P. Barlow W. E. Gralow J. R. Puhalla S. L. Anders C. K. (2023). Cisplatin with veliparib or placebo in metastatic triple-negative breast cancer and BRCA mutation-associated breast cancer (S1416): a randomised, double-blind, placebo-controlled, phase 2 trial. Lancet Oncol. 24 (2), 162–174. 10.1016/S1470-2045(22)00739-2 36623515 PMC9924094

[B319] Rogers C. Erkes D. A. Nardone A. Aplin A. E. Fernandes-Alnemri T. Alnemri E. S. (2019). Gasdermin pores permeabilize mitochondria to augment caspase-3 activation during apoptosis and inflammasome activation. Nat. Commun. 10 (1), 1689. 10.1038/s41467-019-09397-2 30976076 PMC6459836

[B320] Roh J. L. Kim E. H. Jang H. J. Park J. Y. Shin D. (2016). Induction of ferroptotic cell death for overcoming cisplatin resistance of head and neck cancer. Cancer Lett. 381 (1), 96–103. 10.1016/j.canlet.2016.07.035 27477897

[B321] Rosenfeld M. R. Ye X. Supko J. G. Desideri S. Grossman S. A. Brem S. (2014). A phase I/II trial of hydroxychloroquine in conjunction with radiation therapy and concurrent and adjuvant temozolomide in patients with newly diagnosed glioblastoma multiforme. Autophagy 10 (8), 1359–1368. 10.4161/auto.28984 24991840 PMC4203513

[B322] Rudin C. M. Hann C. L. Garon E. B. Ribeiro de Oliveira M. Bonomi P. D. Camidge D. R. (2012). Phase II study of single-agent navitoclax (ABT-263) and biomarker correlates in patients with relapsed small cell lung cancer. Clin. Cancer Res. 18 (11), 3163–3169. 10.1158/1078-0432.CCR-11-3090 22496272 PMC3715059

[B323] Ruiz L. M. Libedinsky A. Elorza A. A. (2021). Role of copper on mitochondrial function and metabolism. Front. Mol. Biosci. 8, 711227. 10.3389/fmolb.2021.711227 34504870 PMC8421569

[B324] Russo A. L. Kwon H. C. Burgan W. E. Carter D. Beam K. Weizheng X. (2009). *In vitro* and *in vivo* radiosensitization of glioblastoma cells by the poly (ADP-ribose) polymerase inhibitor E7016. Clin. Cancer Res. 15 (2), 607–612. 10.1158/1078-0432.CCR-08-2079 19147766 PMC6322204

[B325] Saddoughi S. A. Gencer S. Peterson Y. K. Ward K. E. Mukhopadhyay A. Oaks J. (2013). Sphingosine analogue drug FTY720 targets I2PP2A/SET and mediates lung tumour suppression via activation of PP2A-RIPK1-dependent necroptosis. EMBO Mol. Med. 5 (1), 105–121. 10.1002/emmm.201201283 23180565 PMC3569657

[B326] Safi R. Nelson E. R. Chitneni S. K. Franz K. J. George D. J. Zalutsky M. R. (2014). Copper signaling axis as a target for prostate cancer therapeutics. Cancer Res. 74 (20), 5819–5831. 10.1158/0008-5472.CAN-13-3527 25320179 PMC4203427

[B327] Saghatelyan T. Tananyan A. Janoyan N. Tadevosyan A. Petrosyan H. Hovhannisyan A. (2020). Efficacy and safety of curcumin in combination with paclitaxel in patients with advanced, metastatic breast cancer: a comparative, randomized, double-blind, placebo-controlled clinical trial. Phytomedicine 70, 153218. 10.1016/j.phymed.2020.153218 32335356

[B328] Saha S. Panigrahi D. P. Patil S. Bhutia S. K. (2018). Autophagy in health and disease: a comprehensive review. Biomed. Pharmacother. 104, 485–495. 10.1016/j.biopha.2018.05.007 29800913

[B329] Salazar R. Garcia-Carbonero R. Libutti S. K. Hendifar A. E. Custodio A. Guimbaud R. (2018). Phase II study of BEZ235 versus everolimus in patients with mammalian target of rapamycin inhibitor-naive advanced pancreatic neuroendocrine tumors. Oncologist 23 (7), 766–e90. 10.1634/theoncologist.2017-0144 29242283 PMC6058330

[B330] Sandhu S. K. Schelman W. R. Wilding G. Moreno V. Baird R. D. Miranda S. (2013). The poly(ADP-ribose) polymerase inhibitor niraparib (MK4827) in BRCA mutation carriers and patients with sporadic cancer: a phase 1 dose-escalation trial. Lancet Oncol. 14 (9), 882–892. 10.1016/S1470-2045(13)70240-7 23810788

[B331] Sari A. N. Elwakeel A. Dhanjal J. K. Kumar V. Sundar D. Kaul S. C. (2021). Identification and characterization of mortaparib(plus)-A novel triazole derivative that targets mortalin-p53 interaction and inhibits cancer-cell proliferation by wild-type p53-dependent and -independent mechanisms. Cancers (Basel) 13 (4), 835. 10.3390/cancers13040835 33671256 PMC7921971

[B332] Sato H. Hiraki M. Namba T. Egawa N. Baba K. Tanaka T. (2018). Andrographolide induces degradation of mutant p53 via activation of Hsp70. Int. J. Oncol. 53 (2), 761–770. 10.3892/ijo.2018.4416 29845212

[B333] Satta T. Grant S. (2020). Enhancing venetoclax activity in hematological malignancies. Expert Opin. Investig. Drugs 29 (7), 697–708. 10.1080/13543784.2020.1789588 PMC7529910 32600066

[B334] Sayyid R. K. Bernardino R. Chavarriaga J. Gleave A. Kumar R. Fleshner N. E. (2024). Rucaparib monotherapy in the heavily pre-treated metastatic castrate-resistant prostate cancer setting: practical considerations and alternate treatment approaches. Transl. Androl. Urol. 13 (5), 884–888. 10.21037/tau-23-671 38855585 PMC11157394

[B335] Schroder M. Kaufman R. J. (2005). ER stress and the unfolded protein response. Mutat. Res. 569 (1-2), 29–63. 10.1016/j.mrfmmm.2004.06.056 15603751

[B336] Sciegienka S. J. Solst S. R. Falls K. C. Schoenfeld J. D. Klinger A. R. Ross N. L. (2017). D-penicillamine combined with inhibitors of hydroperoxide metabolism enhances lung and breast cancer cell responses to radiation and carboplatin via H(2)O(2)-mediated oxidative stress. Free Radic. Biol. Med. 108, 354–361. 10.1016/j.freeradbiomed.2017.04.001 28389407 PMC5495544

[B337] Secchiero P. Bosco R. Celeghini C. Zauli G. (2011). Recent advances in the therapeutic perspectives of Nutlin-3. Curr. Pharm. Des. 17 (6), 569–577. 10.2174/138161211795222586 21391907

[B338] Seehawer M. Heinzmann F. D'Artista L. Harbig J. Roux P. F. Hoenicke L. (2018). Necroptosis microenvironment directs lineage commitment in liver cancer. Nature 562 (7725), 69–75. 10.1038/s41586-018-0519-y 30209397 PMC8111790

[B339] Sever R. Brugge J. S. (2015). Signal transduction in cancer. Cold Spring Harb. Perspect. Med. 5 (4), a006098. 10.1101/cshperspect.a006098 25833940 PMC4382731

[B340] Shah M. Green J. Hudacko R. Cohen A. J. (2024). Clinical response to olaparib in a patient with leptomeningeal carcinomatosis in newly diagnosed breast cancer with germline BRCA2 mutation. JCO Precis. Oncol. 8, e2400063. 10.1200/PO.24.00063 38991180

[B341] Sharifi-Rad J. Herrera-Bravo J. Kamiloglu S. Petroni K. Mishra A. P. Monserrat-Mesquida M. (2022). Recent advances in the therapeutic potential of emodin for human health. Biomed. Pharmacother. 154, 113555. 10.1016/j.biopha.2022.113555 36027610

[B342] Sharma S. de Vries E. G. Infante J. R. Oldenhuis C. N. Gietema J. A. Yang L. (2014). Safety, pharmacokinetics, and pharmacodynamics of the DR5 antibody LBY135 alone and in combination with capecitabine in patients with advanced solid tumors. Invest. New Drugs 32 (1), 135–144. 10.1007/s10637-013-9952-9 23589214

[B343] Shi J. Zhao Y. Wang K. Shi X. Wang Y. Huang H. (2015). Cleavage of GSDMD by inflammatory caspases determines pyroptotic cell death. Nature 526 (7575), 660–665. 10.1038/nature15514 26375003

[B344] Shi J. Zhao Y. Wang Y. Gao W. Ding J. Li P. (2014a). Inflammatory caspases are innate immune receptors for intracellular LPS. Nature 514 (7521), 187–192. 10.1038/nature13683 25119034

[B345] Shi Y. Zhou F. Jiang F. Lu H. Wang J. Cheng C. (2014b). PARP inhibitor reduces proliferation and increases apoptosis in breast cancer cells. Chin. J. Cancer Res. 26 (2), 142–147. 10.3978/j.issn.1000-9604.2014.02.13 24826054 PMC4000905

[B346] Shimada K. Skouta R. Kaplan A. Yang W. S. Hayano M. Dixon S. J. (2016). Global survey of cell death mechanisms reveals metabolic regulation of ferroptosis. Nat. Chem. Biol. 12 (7), 497–503. 10.1038/nchembio.2079 27159577 PMC4920070

[B347] Shimada O. Wu X. Jin X. Nouh M. A. Fiscella M. Albert V. (2007). Human agonistic antibody to tumor necrosis factor-related apoptosis-inducing ligand receptor 2 induces cytotoxicity and apoptosis in prostate cancer and bladder cancer cells. Urology 69 (2), 395–401. 10.1016/j.urology.2006.12.007 17320696

[B348] Shimony S. Stone R. M. Stahl M. (2022). Venetoclax combination therapy in acute myeloid leukemia and myelodysplastic syndromes. Curr. Opin. Hematol. 29 (2), 63–73. 10.1097/MOH.0000000000000698 34966123

[B349] Singh J. Novik Y. Stein S. Volm M. Meyers M. Smith J. (2014). Phase 2 trial of everolimus and carboplatin combination in patients with triple negative metastatic breast cancer. Breast Cancer Res. 16 (2), R32. 10.1186/bcr3634 24684785 PMC4053575

[B350] Sinha B. K. Murphy C. Brown S. M. Silver B. B. Tokar E. J. Bortner C. D. (2024). Mechanisms of cell death induced by erastin in human ovarian tumor cells. Int. J. Mol. Sci. 25 (16), 8666. 10.3390/ijms25168666 39201357 PMC11355013

[B351] Slamon D. J. Dieras V. Rugo H. S. Harbeck N. Im S. A. Gelmon K. A. (2024). Overall survival with palbociclib plus letrozole in advanced breast cancer. J. Clin. Oncol. 42 (9), 994–1000. 10.1200/JCO.23.00137 38252901 PMC10950136

[B352] Soares J. Espadinha M. Raimundo L. Ramos H. Gomes A. S. Gomes S. (2017). DIMP53-1: a novel small-molecule dual inhibitor of p53-MDM2/X interactions with multifunctional p53-dependent anticancer properties. Mol. Oncol. 11 (6), 612–627. 10.1002/1878-0261.12051 28296148 PMC5467495

[B353] Son Y. An Y. Jung J. Shin S. Park I. Gwak J. (2019). Protopine isolated from Nandina domestica induces apoptosis and autophagy in colon cancer cells by stabilizing p53. Phytother. Res. 33 (6), 1689–1696. 10.1002/ptr.6357 30932278

[B354] Song B. Wang W. Tang X. Goh R. M. W. Thuya W. L. Ho P. C. L. (2023). Inhibitory potential of resveratrol in cancer metastasis: from biology to therapy. Cancers (Basel) 15 (10), 2758. 10.3390/cancers15102758 37345095 PMC10216034

[B355] Song M. Xia W. Tao Z. Zhu B. Zhang W. Liu C. (2021). Self-assembled polymeric nanocarrier-mediated co-delivery of metformin and doxorubicin for melanoma therapy. Drug Deliv. 28 (1), 594–606. 10.1080/10717544.2021.1898703 33729072 PMC7996084

[B356] Sonkusre P. (2019). Specificity of biogenic selenium nanoparticles for prostate cancer therapy with reduced risk of toxicity: an *in vitro* and *in vivo* study. Front. Oncol. 9, 1541. 10.3389/fonc.2019.01541 32010628 PMC6978793

[B357] Souers A. J. Leverson J. D. Boghaert E. R. Ackler S. L. Catron N. D. Chen J. (2013). ABT-199, a potent and selective BCL-2 inhibitor, achieves antitumor activity while sparing platelets. Nat. Med. 19 (2), 202–208. 10.1038/nm.3048 23291630

[B358] Spencer B. G. Finnie J. W. (2020). The role of endoplasmic reticulum stress in cell survival and death. J. Comp. Pathol. 181, 86–91. 10.1016/j.jcpa.2020.10.006 33288157

[B359] Springer C. Humayun D. Skouta R. (2024). Cuproptosis: unraveling the mechanisms of copper-induced cell death and its implication in cancer therapy. Cancers (Basel) 16 (3), 647. 10.3390/cancers16030647 38339398 PMC10854864

[B360] Sterling J. Guttha S. Song Y. Song D. Hadziahmetovic M. Dunaief J. L. (2017). Iron importers Zip8 and Zip14 are expressed in retina and regulated by retinal iron levels. Exp. Eye Res. 155, 15–23. 10.1016/j.exer.2016.12.008 28057442 PMC5359041

[B361] Sui X. Chen R. Wang Z. Huang Z. Kong N. Zhang M. (2013). Autophagy and chemotherapy resistance: a promising therapeutic target for cancer treatment. Cell Death Dis. 4 (10), e838. 10.1038/cddis.2013.350 24113172 PMC3824660

[B362] Sun J. Wei Q. Zhou Y. Wang J. Liu Q. Xu H. (2017). A systematic analysis of FDA-approved anticancer drugs. BMC Syst. Biol. 11 (Suppl. 5), 87. 10.1186/s12918-017-0464-7 28984210 PMC5629554

[B363] Sun L. Wang H. Wang Z. He S. Chen S. Liao D. (2012). Mixed lineage kinase domain-like protein mediates necrosis signaling downstream of RIP3 kinase. Cell 148 (1-2), 213–227. 10.1016/j.cell.2011.11.031 22265413

[B364] Sun W. Li J. (2024). Efficacy and safety of veliparib in the treatment of advanced/metastatic breast cancer: a meta-analysis of phase II and III randomized controlled trials. J. Chemother. 36 (6), 441–448. 10.1080/1120009X.2023.2281760 37975589

[B365] Sussman R. T. Ricci M. S. Hart L. S. Sun S. Y. El-Deiry W. S. (2007). Chemotherapy-resistant side-population of colon cancer cells has a higher sensitivity to TRAIL than the non-SP, a higher expression of c-Myc and TRAIL-receptor DR4. Cancer Biol. Ther. 6 (9), 1490–1495. 10.4161/cbt.6.9.4905 17881904

[B366] Swanton C. Bernard E. Abbosh C. Andre F. Auwerx J. Balmain A. (2024). Embracing cancer complexity: hallmarks of systemic disease. Cell 187 (7), 1589–1616. 10.1016/j.cell.2024.02.009 38552609 PMC12077170

[B367] Tadele D. S. Robertson J. Crispin R. Herrera M. C. Chlubnova M. Piechaczyk L. (2021). A cell competition-based small molecule screen identifies a novel compound that induces dual c-Myc depletion and p53 activation. J. Biol. Chem. 296, 100179. 10.1074/jbc.RA120.015285 33303632 PMC7948465

[B368] Tamm I. Kornblau S. M. Segall H. Krajewski S. Welsh K. Kitada S. (2000). Expression and prognostic significance of IAP-family genes in human cancers and myeloid leukemias. Clin. Cancer Res. 6 (5), 1796–1803. 10815900

[B369] Tan T. Li J. Luo R. Wang R. Yin L. Liu M. (2021). Recent advances in understanding the mechanisms of elemene in reversing drug resistance in tumor cells: a review. Molecules 26 (19), 5792. 10.3390/molecules26195792 34641334 PMC8510449

[B370] Tang D. Kang R. Berghe T. V. Vandenabeele P. Kroemer G. (2019). The molecular machinery of regulated cell death. Cell Res. 29 (5), 347–364. 10.1038/s41422-019-0164-5 30948788 PMC6796845

[B371] Tang F. Hu P. Yang Z. Xue C. Gong J. Sun S. (2017). SBI0206965, a novel inhibitor of Ulk1, suppresses non-small cell lung cancer cell growth by modulating both autophagy and apoptosis pathways. Oncol. Rep. 37 (6), 3449–3458. 10.3892/or.2017.5635 28498429

[B372] Tang M. Crown J. Duffy M. J. (2023). Degradation of MYC by the mutant p53 reactivator drug, COTI-2 in breast cancer cells. Invest. New Drugs 41 (4), 541–550. 10.1007/s10637-023-01368-1 37233863 PMC10447602

[B373] Tang R. Xu J. Zhang B. Liu J. Liang C. Hua J. (2020). Ferroptosis, necroptosis, and pyroptosis in anticancer immunity. J. Hematol. Oncol. 13 (1), 110. 10.1186/s13045-020-00946-7 32778143 PMC7418434

[B374] Taniguchi K. Yamachika S. He F. Karin M. (2016). p62/SQSTM1-Dr. Jekyll and Mr. Hyde that prevents oxidative stress but promotes liver cancer. FEBS Lett. 590 (15), 2375–2397. 10.1002/1873-3468.12301 27404485 PMC4983218

[B375] Tao Z. Le Blanc J. M. Wang C. Zhan T. Zhuang H. Wang P. (2016). Coadministration of trametinib and palbociclib radiosensitizes KRAS-mutant non-small cell lung cancers *in vitro* and *in vivo* . Clin. Cancer Res. 22 (1), 122–133. 10.1158/1078-0432.CCR-15-0589 26728409

[B376] Telli M. L. Litton J. K. Beck J. T. Jones J. M. Andersen J. Mina L. A. (2024). Neoadjuvant talazoparib in patients with germline BRCA1/2 mutation-positive, early-stage triple-negative breast cancer: exploration of tumor BRCA mutational status. Breast Cancer 31 (5), 886–897. 10.1007/s12282-024-01603-4 38869771 PMC11341741

[B377] Tong X. Tang R. Xiao M. Xu J. Wang W. Zhang B. (2022). Targeting cell death pathways for cancer therapy: recent developments in necroptosis, pyroptosis, ferroptosis, and cuproptosis research. J. Hematol. Oncol. 15 (1), 174. 10.1186/s13045-022-01392-3 36482419 PMC9733270

[B378] Trapani J. A. Smyth M. J. (2002). Functional significance of the perforin/granzyme cell death pathway. Nat. Rev. Immunol. 2 (10), 735–747. 10.1038/nri911 12360212

[B379] Trarbach T. Moehler M. Heinemann V. Kohne C. H. Przyborek M. Schulz C. (2010). Phase II trial of mapatumumab, a fully human agonistic monoclonal antibody that targets and activates the tumour necrosis factor apoptosis-inducing ligand receptor-1 (TRAIL-R1), in patients with refractory colorectal cancer. Br. J. Cancer 102 (3), 506–512. 10.1038/sj.bjc.6605507 20068564 PMC2822942

[B380] Tron A. E. Belmonte M. A. Adam A. Aquila B. M. Boise L. H. Chiarparin E. (2018). Discovery of Mcl-1-specific inhibitor AZD5991 and preclinical activity in multiple myeloma and acute myeloid leukemia. Nat. Commun. 9 (1), 5341. 10.1038/s41467-018-07551-w 30559424 PMC6297231

[B381] Tsang T. Gu X. Davis C. I. Posimo J. M. Miller Z. A. Brady D. C. (2022). BRAFV600E-Driven lung adenocarcinoma requires copper to sustain autophagic signaling and processing. Mol. Cancer Res. 20 (7), 1096–1107. 10.1158/1541-7786.MCR-21-0250 35320362 PMC9262833

[B382] Tse C. Shoemaker A. R. Adickes J. Anderson M. G. Chen J. Jin S. (2008). ABT-263: a potent and orally bioavailable Bcl-2 family inhibitor. Cancer Res. 68 (9), 3421–3428. 10.1158/0008-5472.CAN-07-5836 18451170

[B383] Tsvetkov P. Coy S. Petrova B. Dreishpoon M. Verma A. Abdusamad M. (2022). Copper induces cell death by targeting lipoylated TCA cycle proteins. Science 375 (6586), 1254–1261. 10.1126/science.abf0529 35298263 PMC9273333

[B384] Tsvetkov P. Detappe A. Cai K. Keys H. R. Brune Z. Ying W. (2019). Mitochondrial metabolism promotes adaptation to proteotoxic stress. Nat. Chem. Biol. 15 (7), 681–689. 10.1038/s41589-019-0291-9 31133756 PMC8183600

[B385] Tucci M. Stucci S. Savonarola A. Resta L. Cives M. Rossi R. (2014). An imbalance between Beclin-1 and p62 expression promotes the proliferation of myeloma cells through autophagy regulation. Exp. Hematol. 42 (10), 897–908. 10.1016/j.exphem.2014.06.005 24971696

[B386] Tufail M. Hu J. J. Liang J. He C. Y. Wan W. D. Huang Y. Q. (2024). Hallmarks of cancer resistance. iScience 27 (6), 109979. 10.1016/j.isci.2024.109979 38832007 PMC11145355

[B387] Tzifi F. Economopoulou C. Gourgiotis D. Ardavanis A. Papageorgiou S. Scorilas A. (2012). The role of BCL2 family of apoptosis regulator proteins in acute and chronic leukemias. Adv. Hematol. 2012, 524308. 10.1155/2012/524308 21941553 PMC3173728

[B388] Vandenabeele P. Galluzzi L. Vanden Berghe T. Kroemer G. (2010). Molecular mechanisms of necroptosis: an ordered cellular explosion. Nat. Rev. Mol. Cell Biol. 11 (10), 700–714. 10.1038/nrm2970 20823910

[B389] Van Hoecke L. Riederer S. Saelens X. Sutter G. Rojas J. J. (2020). Recombinant viruses delivering the necroptosis mediator MLKL induce a potent antitumor immunity in mice. Oncoimmunology 9 (1), 1802968. 10.1080/2162402X.2020.1802968 32923163 PMC7458643

[B390] Varisli L. Cen O. Vlahopoulos S. (2020). Dissecting pharmacological effects of chloroquine in cancer treatment: interference with inflammatory signaling pathways. Immunology 159 (3), 257–278. 10.1111/imm.13160 31782148 PMC7011648

[B391] Viswanathan V. S. Ryan M. J. Dhruv H. D. Gill S. Eichhoff O. M. Seashore-Ludlow B. (2017). Dependency of a therapy-resistant state of cancer cells on a lipid peroxidase pathway. Nature 547 (7664), 453–457. 10.1038/nature23007 28678785 PMC5667900

[B392] Vogl D. T. Stadtmauer E. A. Tan K. S. Heitjan D. F. Davis L. E. Pontiggia L. (2014). Combined autophagy and proteasome inhibition: a phase 1 trial of hydroxychloroquine and bortezomib in patients with relapsed/refractory myeloma. Autophagy 10 (8), 1380–1390. 10.4161/auto.29264 24991834 PMC4203515

[B393] von Pawel J. Harvey J. H. Spigel D. R. Dediu M. Reck M. Cebotaru C. L. (2014). Phase II trial of mapatumumab, a fully human agonist monoclonal antibody to tumor necrosis factor-related apoptosis-inducing ligand receptor 1 (TRAIL-R1), in combination with paclitaxel and carboplatin in patients with advanced non-small-cell lung cancer. Clin. Lung Cancer 15 (3), 188–196. 10.1016/j.cllc.2013.12.005 24560012

[B394] Vuorinen R. L. Paunu N. Turpeenniemi-Hujanen T. Reunamo T. Jekunen A. Kataja V. (2019). Sunitinib first-line treatment in metastatic renal cell carcinoma: costs and effects. Anticancer Res. 39 (10), 5559–5564. 10.21873/anticanres.13749 31570450

[B395] Waarts M. R. Stonestrom A. J. Park Y. C. Levine R. L. (2022). Targeting mutations in cancer. J. Clin. Invest. 132 (8), e154943. 10.1172/JCI154943 35426374 PMC9012285

[B396] Walensky L. D. Kung A. L. Escher I. Malia T. J. Barbuto S. Wright R. D. (2004). Activation of apoptosis *in vivo* by a hydrocarbon-stapled BH3 helix. Science 305 (5689), 1466–1470. 10.1126/science.1099191 15353804 PMC1360987

[B397] Wang C. Youle R. J. (2009). The role of mitochondria in apoptosis. Annu. Rev. Genet. 43, 95–118. 10.1146/annurev-genet-102108-134850 19659442 PMC4762029

[B398] Wang F. Gouttia O. G. Wang L. Peng A. (2021a). PARP1 upregulation in recurrent oral cancer and treatment resistance. Front. Cell Dev. Biol. 9, 804962. 10.3389/fcell.2021.804962 35071239 PMC8769238

[B399] Wang H. Rong X. Zhao G. Zhou Y. Xiao Y. Ma D. (2022a). The microbial metabolite trimethylamine N-oxide promotes antitumor immunity in triple-negative breast cancer. Cell Metab. 34 (4), 581–594.e8. 10.1016/j.cmet.2022.02.010 35278352

[B400] Wang H. Sun L. Su L. Rizo J. Liu L. Wang L. F. (2014). Mixed lineage kinase domain-like protein MLKL causes necrotic membrane disruption upon phosphorylation by RIP3. Mol. Cell 54 (1), 133–146. 10.1016/j.molcel.2014.03.003 24703947

[B401] Wang K. Zhang Z. Tsai H. I. Liu Y. Gao J. Wang M. (2021b). Branched-chain amino acid aminotransferase 2 regulates ferroptotic cell death in cancer cells. Cell Death Differ. 28 (4), 1222–1236. 10.1038/s41418-020-00644-4 33097833 PMC8027606

[B402] Wang L. Li K. Lin X. Yao Z. Wang S. Xiong X. (2019a). Metformin induces human esophageal carcinoma cell pyroptosis by targeting the miR-497/PELP1 axis. Cancer Lett. 450, 22–31. 10.1016/j.canlet.2019.02.014 30771436

[B403] Wang Q. Imamura R. Motani K. Kushiyama H. Nagata S. Suda T. (2013). Pyroptotic cells externalize eat-me and release find-me signals and are efficiently engulfed by macrophages. Int. Immunol. 25 (6), 363–372. 10.1093/intimm/dxs161 23446850

[B404] Wang Q. Ren M. Feng F. Chen K. Ju X. (2018a). Treatment of colon cancer with liver X receptor agonists induces immunogenic cell death. Mol. Carcinog. 57 (7), 903–910. 10.1002/mc.22811 29573475

[B405] Wang Q. Shao X. Zhang Y. Zhu M. Wang F. X. C. Mu J. (2023). Role of tumor microenvironment in cancer progression and therapeutic strategy. Cancer Med. 12 (10), 11149–11165. 10.1002/cam4.5698 36807772 PMC10242329

[B406] Wang Q. Wang P. Zhang L. Tessema M. Bai L. Xu X. (2020). Epigenetic regulation of RIP3 suppresses necroptosis and increases resistance to chemotherapy in NonSmall cell lung cancer. Transl. Oncol. 13 (2), 372–382. 10.1016/j.tranon.2019.11.011 31887632 PMC6938879

[B407] Wang Q. Zhou J. Cheng A. Liu Y. Guo J. Li X. (2024a). Artesunate-binding FABP5 promotes apoptosis in lung cancer cells via the PPARγ-SCD pathway. Int. Immunopharmacol. 143 (Pt 1), 113381. 10.1016/j.intimp.2024.113381 39405934

[B408] Wang T. Liu Y. Li Q. Luo Y. Liu D. Li B. (2022b). Cuproptosis-related gene FDX1 expression correlates with the prognosis and tumor immune microenvironment in clear cell renal cell carcinoma. Front. Immunol. 13, 999823. 10.3389/fimmu.2022.999823 36225932 PMC9549781

[B409] Wang W. Green M. Choi J. E. Gijon M. Kennedy P. D. Johnson J. K. (2019b). CD8(+) T cells regulate tumour ferroptosis during cancer immunotherapy. Nature 569 (7755), 270–274. 10.1038/s41586-019-1170-y 31043744 PMC6533917

[B410] Wang W. Lu Z. Wang M. Liu Z. Wu B. Yang C. (2022c). The cuproptosis-related signature associated with the tumor environment and prognosis of patients with glioma. Front. Immunol. 13, 998236. 10.3389/fimmu.2022.998236 36110851 PMC9468372

[B411] Wang W. Zhang L. Sun Z. (2022d). Eliciting pyroptosis to fuel cancer immunotherapy: mechanisms and strategies. Cancer Biol. Med. 19 (7), 948–964. 10.20892/j.issn.2095-3941.2022.0049 35856558 PMC9334758

[B412] Wang X. Chen Y. Wang X. Tian H. Wang Y. Jin J. (2021c). Stem cell factor SOX2 confers ferroptosis resistance in lung cancer via upregulation of SLC7A11. Cancer Res. 81 (20), 5217–5229. 10.1158/0008-5472.CAN-21-0567 34385181 PMC8530936

[B413] Wang X. Xu S. Zhang L. Cheng X. Yu H. Bao J. (2021d). Vitamin C induces ferroptosis in anaplastic thyroid cancer cells by ferritinophagy activation. Biochem. Biophys. Res. Commun. 551, 46–53. 10.1016/j.bbrc.2021.02.126 33714759

[B414] Wang Y. Chen Y. Zhang J. Yang Y. Fleishman J. S. Wang Y. (2024b). Cuproptosis: a novel therapeutic target for overcoming cancer drug resistance. Drug Resist Updat 72, 101018. 10.1016/j.drup.2023.101018 37979442

[B415] Wang Y. Luo W. Wang Y. (2019c). PARP-1 and its associated nucleases in DNA damage response. DNA Repair (Amst) 81, 102651. 10.1016/j.dnarep.2019.102651 31302005 PMC6764844

[B416] Wang Y. Peng R. Q. Li D. D. Ding Y. Wu X. Q. Zeng Y. X. (2011). Chloroquine enhances the cytotoxicity of topotecan by inhibiting autophagy in lung cancer cells. Chin. J. Cancer 30 (10), 690–700. 10.5732/cjc.011.10056 21959046 PMC4012269

[B417] Wang Y. Yin B. Li D. Wang G. Han X. Sun X. (2018b). GSDME mediates caspase-3-dependent pyroptosis in gastric cancer. Biochem. Biophys. Res. Commun. 495 (1), 1418–1425. 10.1016/j.bbrc.2017.11.156 29183726

[B418] Wang Y. Zhang L. Zhou F. (2022e). Cuproptosis: a new form of programmed cell death. Cell Mol. Immunol. 19 (8), 867–868. 10.1038/s41423-022-00866-1 35459854 PMC9338229

[B419] Wang Y. H. Scadden D. T. (2015). Harnessing the apoptotic programs in cancer stem-like cells. EMBO Rep. 16 (9), 1084–1098. 10.15252/embr.201439675 26253117 PMC4576979

[B420] Wang Z. Yao J. Dong T. Niu X. (2022f). Definition of a novel cuproptosis-relevant lncRNA signature for uncovering distinct survival, genomic alterations, and treatment implications in lung adenocarcinoma. J. Immunol. Res. 2022, 2756611. 10.1155/2022/2756611 36281357 PMC9587678

[B421] Wei A. H. Roberts A. W. Spencer A. Rosenberg A. S. Siegel D. Walter R. B. (2020). Targeting MCL-1 in hematologic malignancies: rationale and progress. Blood Rev. 44, 100672. 10.1016/j.blre.2020.100672 32204955 PMC7442684

[B422] Wei X. Xie F. Zhou X. Wu Y. Yan H. Liu T. (2022). Role of pyroptosis in inflammation and cancer. Cell Mol. Immunol. 19 (9), 971–992. 10.1038/s41423-022-00905-x 35970871 PMC9376585

[B423] Wertz I. E. Kusam S. Lam C. Okamoto T. Sandoval W. Anderson D. J. (2011). Sensitivity to antitubulin chemotherapeutics is regulated by MCL1 and FBW7. Nature 471 (7336), 110–114. 10.1038/nature09779 21368834

[B424] Widden H. Placzek W. J. (2021). The multiple mechanisms of MCL1 in the regulation of cell fate. Commun. Biol. 4 (1), 1029. 10.1038/s42003-021-02564-6 34475520 PMC8413315

[B425] Wise-Draper T. M. Moorthy G. Salkeni M. A. Karim N. A. Thomas H. E. Mercer C. A. (2017). A phase ib study of the dual PI3K/mTOR inhibitor dactolisib (BEZ235) combined with everolimus in patients with advanced solid malignancies. Target Oncol. 12 (3), 323–332. 10.1007/s11523-017-0482-9 28357727 PMC5447332

[B426] Wolpin B. M. Rubinson D. A. Wang X. Chan J. A. Cleary J. M. Enzinger P. C. (2014). Phase II and pharmacodynamic study of autophagy inhibition using hydroxychloroquine in patients with metastatic pancreatic adenocarcinoma. Oncologist 19 (6), 637–638. 10.1634/theoncologist.2014-0086 24821822 PMC4041680

[B427] Workenhe S. T. Nguyen A. Bakhshinyan D. Wei J. Hare D. N. MacNeill K. L. (2020). *De novo* necroptosis creates an inflammatory environment mediating tumor susceptibility to immune checkpoint inhibitors. Commun. Biol. 3 (1), 645. 10.1038/s42003-020-01362-w 33149194 PMC7643076

[B428] Wu F. Huang F. Jiang N. Su J. Yao S. Liang B. (2024a). Identification of ferroptosis related genes and pathways in prostate cancer cells under erastin exposure. BMC Urol. 24 (1), 78. 10.1186/s12894-024-01472-1 38575966 PMC10996193

[B429] Wu H. Lin J. Liu P. Huang Z. Zhao P. Jin H. (2015). Is the autophagy a friend or foe in the silver nanoparticles associated radiotherapy for glioma? Biomaterials 62, 47–57. 10.1016/j.biomaterials.2015.05.033 26022979

[B430] Wu M. Wang Y. Yang D. Gong Y. Rao F. Liu R. (2019a). A PLK1 kinase inhibitor enhances the chemosensitivity of cisplatin by inducing pyroptosis in oesophageal squamous cell carcinoma. EBioMedicine 41, 244–255. 10.1016/j.ebiom.2019.02.012 30876762 PMC6442225

[B431] Wu W. Liu P. Li J. (2012). Necroptosis: an emerging form of programmed cell death. Crit. Rev. Oncol. Hematol. 82 (3), 249–258. 10.1016/j.critrevonc.2011.08.004 21962882

[B432] Wu X. Zhu J. Yin R. Yang J. Liu J. Wang J. (2024b). Niraparib maintenance therapy using an individualised starting dose in patients with platinum-sensitive recurrent ovarian cancer (NORA): final overall survival analysis of a phase 3 randomised, placebo-controlled trial. EClinicalMedicine 72, 102629. 10.1016/j.eclinm.2024.102629 38745967 PMC11090914

[B433] Wu Y. Dong G. Sheng C. (2020). Targeting necroptosis in anticancer therapy: mechanisms and modulators. Acta Pharm. Sin. B 10 (9), 1601–1618. 10.1016/j.apsb.2020.01.007 33088682 PMC7563021

[B434] Wu Z. Zhang W. Kang Y. J. (2019b). Copper affects the binding of HIF-1α to the critical motifs of its target genes. Metallomics 11 (2), 429–438. 10.1039/c8mt00280k 30566157

[B435] Xie J. Yang Y. Gao Y. He J. (2023a). Cuproptosis: mechanisms and links with cancers. Mol. Cancer 22 (1), 46. 10.1186/s12943-023-01732-y 36882769 PMC9990368

[B436] Xie Y. Kang R. Klionsky D. J. Tang D. (2023b). GPX4 in cell death, autophagy, and disease. Autophagy 19 (10), 2621–2638. 10.1080/15548627.2023.2218764 37272058 PMC10472888

[B437] Xie Y. Zhu S. Song X. Sun X. Fan Y. Liu J. (2017). The tumor suppressor p53 limits ferroptosis by blocking DPP4 activity. Cell Rep. 20 (7), 1692–1704. 10.1016/j.celrep.2017.07.055 28813679

[B438] Xu Y. Liu S. Y. Zeng L. Ma H. Zhang Y. Yang H. (2022). An enzyme-engineered nonporous copper(I) coordination polymer nanoplatform for cuproptosis-based synergistic cancer therapy. Adv. Mater 34 (43), e2204733. 10.1002/adma.202204733 36054475

[B439] Xuzhang W. Lu T. Jin W. Yu Y. Li Z. Shen L. (2024). Cisplatin-induced pyroptosis enhances the efficacy of PD-L1 inhibitor in small-cell lung cancer via GSDME/IL12/CD4Tem Axis. Int. J. Biol. Sci. 20 (2), 537–553. 10.7150/ijbs.89080 38169676 PMC10758111

[B440] Yan H. Luo B. Wu X. Guan F. Yu X. Zhao L. (2021). Cisplatin induces pyroptosis via activation of MEG3/NLRP3/caspase-1/GSDMD pathway in triple-negative breast cancer. Int. J. Biol. Sci. 17 (10), 2606–2621. 10.7150/ijbs.60292 34326697 PMC8315016

[B441] Yan J. Wan P. Choksi S. Liu Z. G. (2022). Necroptosis and tumor progression. Trends Cancer 8 (1), 21–27. 10.1016/j.trecan.2021.09.003 34627742 PMC8702466

[B442] Yang B. Jiang J. Wu H. Lu Q. (2024). Topical BCl-2 inhibitor (ABT-737) attenuates skin photoaging in mice. Exp. Dermatol 33 (3), e15051. 10.1111/exd.15051 38514923

[B443] Yang D. Shu T. Zhao H. Sun Y. Xu W. Tu G. (2020). Knockdown of macrophage migration inhibitory factor (MIF), a novel target to protect neurons from parthanatos induced by simulated post-spinal cord injury oxidative stress. Biochem. Biophys. Res. Commun. 523 (3), 719–725. 10.1016/j.bbrc.2019.12.115 31948762

[B444] Yang L. Kumar B. Shen C. Zhao S. Blakaj D. Li T. (2019). LCL161, a SMAC-mimetic, preferentially radiosensitizes human papillomavirus-negative head and neck squamous cell carcinoma. Mol. Cancer Ther. 18 (6), 1025–1035. 10.1158/1535-7163.MCT-18-1157 31015310 PMC6548673

[B445] Yang M. Wu X. Hu J. Wang Y. Wang Y. Zhang L. (2022). COMMD10 inhibits HIF1α/CP loop to enhance ferroptosis and radiosensitivity by disrupting Cu-Fe balance in hepatocellular carcinoma. J. Hepatol. 76 (5), 1138–1150. 10.1016/j.jhep.2022.01.009 35101526

[B446] Yang W. S. SriRamaratnam R. Welsch M. E. Shimada K. Skouta R. Viswanathan V. S. (2014). Regulation of ferroptotic cancer cell death by GPX4. Cell 156 (1-2), 317–331. 10.1016/j.cell.2013.12.010 24439385 PMC4076414

[B447] Yang Y. Hu W. Feng S. Ma J. Wu M. (2005). RIP3 beta and RIP3 gamma, two novel splice variants of receptor-interacting protein 3 (RIP3), downregulate RIP3-induced apoptosis. Biochem. Biophys. Res. Commun. 332 (1), 181–187. 10.1016/j.bbrc.2005.04.114 15896315

[B448] Yao X. Xie R. Cao Y. Tang J. Men Y. Peng H. (2021). Simvastatin induced ferroptosis for triple-negative breast cancer therapy. J. Nanobiotechnology 19 (1), 311. 10.1186/s12951-021-01058-1 34627266 PMC8502296

[B449] Ye K. Chen Z. Xu Y. (2023). The double-edged functions of necroptosis. Cell Death Dis. 14 (2), 163. 10.1038/s41419-023-05691-6 36849530 PMC9969390

[B450] Ye L. Jin F. Kumar S. K. Dai Y. (2021a). The mechanisms and therapeutic targets of ferroptosis in cancer. Expert Opin. Ther. Targets 25 (11), 965–986. 10.1080/14728222.2021.2011206 34821176

[B451] Ye L. F. Chaudhary K. R. Zandkarimi F. Harken A. D. Kinslow C. J. Upadhyayula P. S. (2020). Radiation-induced lipid peroxidation triggers ferroptosis and synergizes with ferroptosis inducers. ACS Chem. Biol. 15 (2), 469–484. 10.1021/acschembio.9b00939 31899616 PMC7180072

[B452] Ye Z. Q. Chen H. B. Zhang T. Y. Chen Z. Tian L. Gu D. N. (2021b). MicroRNA-7 modulates cellular senescence to relieve gemcitabine resistance by targeting PARP1/NF-κB signaling in pancreatic cancer cells. Oncol. Lett. 21 (2), 139. 10.3892/ol.2020.12400 33552258 PMC7798037

[B453] Yoshii J. Yoshiji H. Kuriyama S. Ikenaka Y. Noguchi R. Okuda H. (2001). The copper-chelating agent, trientine, suppresses tumor development and angiogenesis in the murine hepatocellular carcinoma cells. Int. J. Cancer 94 (6), 768–773. 10.1002/ijc.1537 11745476

[B454] Younes A. Vose J. M. Zelenetz A. D. Smith M. R. Burris H. A. Ansell S. M. (2010). A Phase 1b/2 trial of mapatumumab in patients with relapsed/refractory non-Hodgkin's lymphoma. Br. J. Cancer 103 (12), 1783–1787. 10.1038/sj.bjc.6605987 21081929 PMC3008610

[B455] Yu J. Li S. Qi J. Chen Z. Wu Y. Guo J. (2019). Cleavage of GSDME by caspase-3 determines lobaplatin-induced pyroptosis in colon cancer cells. Cell Death Dis. 10 (3), 193. 10.1038/s41419-019-1441-4 30804337 PMC6389936

[B456] Yu X. Deng Q. Li W. Xiao L. Luo X. Liu X. (2015). Neoalbaconol induces cell death through necroptosis by regulating RIPK-dependent autocrine TNFα and ROS production. Oncotarget 6 (4), 1995–2008. 10.18632/oncotarget.3038 25575821 PMC4385831

[B457] Yu X. He S. (2017). GSDME as an executioner of chemotherapy-induced cell death. Sci. China Life Sci. 60 (11), 1291–1294. 10.1007/s11427-017-9142-2 29134415

[B458] Yuan B. Liao F. Shi Z. Z. Ren Y. Deng X. L. Yang T. T. (2020). Dihydroartemisinin inhibits the proliferation, colony formation and induces ferroptosis of lung cancer cells by inhibiting PRIM2/slc7a11 Axis. Onco Targets Ther. 13, 10829–10840. 10.2147/OTT.S248492 33149601 PMC7602909

[B459] Yuan J. Song J. Chen C. Lv X. Bai J. Yang J. (2022). Combination of ruxolitinib with ABT-737 exhibits synergistic effects in cells carrying concurrent JAK2(V617F) and ASXL1 mutations. Invest. New Drugs 40 (6), 1194–1205. 10.1007/s10637-022-01297-5 36044173

[B460] Yue E. Tuguzbaeva G. Chen X. Qin Y. Li A. Sun X. (2019). Anthocyanin is involved in the activation of pyroptosis in oral squamous cell carcinoma. Phytomedicine 56, 286–294. 10.1016/j.phymed.2018.09.223 30668350

[B461] Yun J. Mullarky E. Lu C. Bosch K. N. Kavalier A. Rivera K. (2015). Vitamin C selectively kills KRAS and BRAF mutant colorectal cancer cells by targeting GAPDH. Science 350 (6266), 1391–1396. 10.1126/science.aaa5004 26541605 PMC4778961

[B462] Zanardi E. Verzoni E. Grassi P. Necchi A. Giannatempo P. Raggi D. (2015). Clinical experience with temsirolimus in the treatment of advanced renal cell carcinoma. Ther. Adv. Urol. 7 (3), 152–161. 10.1177/1756287215574457 26161146 PMC4485412

[B463] Zhang C. Liu X. Jin S. Chen Y. Guo R. (2022a). Ferroptosis in cancer therapy: a novel approach to reversing drug resistance. Mol. Cancer 21 (1), 47. 10.1186/s12943-022-01530-y 35151318 PMC8840702

[B464] Zhang C. C. Li C. G. Wang Y. F. Xu L. H. He X. H. Zeng Q. Z. (2019a). Chemotherapeutic paclitaxel and cisplatin differentially induce pyroptosis in A549 lung cancer cells via caspase-3/GSDME activation. Apoptosis 24 (3-4), 312–325. 10.1007/s10495-019-01515-1 30710195

[B465] Zhang D. Cui P. Dai Z. Yang B. Yao X. Liu Q. (2019b). Tumor microenvironment responsive FePt/MoS(2) nanocomposites with chemotherapy and photothermal therapy for enhancing cancer immunotherapy. Nanoscale 11 (42), 19912–19922. 10.1039/c9nr05684j 31599915

[B466] Zhang J. Yu G. Yang Y. Wang Y. Guo M. Yin Q. (2022b). A small-molecule inhibitor of MDMX suppresses cervical cancer cells via the inhibition of E6-E6AP-p53 axis. Pharmacol. Res. 177, 106128. 10.1016/j.phrs.2022.106128 35150860

[B467] Zhang N. Hartig H. Dzhagalov I. Draper D. He Y. W. (2005). The role of apoptosis in the development and function of T lymphocytes. Cell Res. 15 (10), 749–769. 10.1038/sj.cr.7290345 16246265

[B468] Zhang T. Yin C. Fedorov A. Qiao L. Bao H. Beknazarov N. (2022c). ADAR1 masks the cancer immunotherapeutic promise of ZBP1-driven necroptosis. Nature 606 (7914), 594–602. 10.1038/s41586-022-04753-7 35614224 PMC9373927

[B469] Zhang X. Walke G. R. Horvath I. Kumar R. Blockhuys S. Holgersson S. (2022d). Memo1 binds reduced copper ions, interacts with copper chaperone Atox1, and protects against copper-mediated redox activity *in vitro* . Proc. Natl. Acad. Sci. U. S. A. 119 (37), e2206905119. 10.1073/pnas.2206905119 36067318 PMC9477392

[B470] Zhang X. Wang H. Yu M. Ma K. Ning L. (2022e). Inhibition of autophagy by 3-methyladenine promotes migration and invasion of colon cancer cells through epithelial mesenchymal transformation. Transl. Cancer Res. 11 (8), 2834–2842. 10.21037/tcr-22-1736 36093546 PMC9459642

[B471] Zhang Y. Chen X. Gueydan C. Han J. (2018). Plasma membrane changes during programmed cell deaths. Cell Res. 28 (1), 9–21. 10.1038/cr.2017.133 29076500 PMC5752838

[B472] Zhang Y. Tan H. Daniels J. D. Zandkarimi F. Liu H. Brown L. M. (2019c). Imidazole ketone erastin induces ferroptosis and slows tumor growth in a mouse lymphoma model. Cell Chem. Biol. 26 (5), 623–633. 10.1016/j.chembiol.2019.01.008 30799221 PMC6525071

[B473] Zhang Z. Lin J. Yang L. Li Y. (2023). Osimertinib inhibits brain metastases and improves long-term survival in a patient with advanced squamous cell lung cancer: a case report and literature review. Front. Oncol. 13, 1188772. 10.3389/fonc.2023.1188772 37781197 PMC10539605

[B474] Zhang Z. Zeng X. Wu Y. Liu Y. Zhang X. Song Z. (2022f). Cuproptosis-related risk score predicts prognosis and characterizes the tumor microenvironment in hepatocellular carcinoma. Front. Immunol. 13, 925618. 10.3389/fimmu.2022.925618 35898502 PMC9311491

[B475] Zhao G. Feng E. Liu Y. (2023). Efficacy and safety of veliparib combined with traditional chemotherapy for treating patients with lung cancer: a comprehensive review and meta-analysis. PeerJ 11, e16402. 10.7717/peerj.16402 37965288 PMC10642362

[B476] Zhao G. Han X. Zheng S. Li Z. Sha Y. Ni J. (2016). Curcumin induces autophagy, inhibits proliferation and invasion by downregulating AKT/mTOR signaling pathway in human melanoma cells. Oncol. Rep. 35 (2), 1065–1074. 10.3892/or.2015.4413 26573768

[B477] Zhao L. Zhou X. Xie F. Zhang L. Yan H. Huang J. (2022). Ferroptosis in cancer and cancer immunotherapy. Cancer Commun. (Lond) 42 (2), 88–116. 10.1002/cac2.12250 35133083 PMC8822596

[B478] Zhao W. Jiang L. Fang T. Fang F. Liu Y. Zhao Y. (2021a). β-Lapachone selectively kills hepatocellular carcinoma cells by targeting NQO1 to induce extensive DNA damage and PARP1 hyperactivation. Front. Oncol. 11, 747282. 10.3389/fonc.2021.747282 34676172 PMC8523939

[B479] Zhao X. Quan J. Tan Y. Liu Y. Liao C. Li Z. (2021b). RIP3 mediates TCN-induced necroptosis through activating mitochondrial metabolism and ROS production in chemotherapy-resistant cancers. Am. J. Cancer Res. 11 (3), 729–745. 33791150 PMC7994173

[B480] Zheng D. Liwinski T. Elinav E. (2020a). Inflammasome activation and regulation: toward a better understanding of complex mechanisms. Cell Discov. 6, 36. 10.1038/s41421-020-0167-x 32550001 PMC7280307

[B481] Zheng M. Williams E. P. Malireddi R. K. S. Karki R. Banoth B. Burton A. (2020b). Impaired NLRP3 inflammasome activation/pyroptosis leads to robust inflammatory cell death via caspase-8/RIPK3 during coronavirus infection. J. Biol. Chem. 295 (41), 14040–14052. 10.1074/jbc.RA120.015036 32763970 PMC7549031

[B482] Zheng Z. Bian Y. Zhang Y. Ren G. Li G. (2020c). Metformin activates AMPK/SIRT1/NF-κB pathway and induces mitochondrial dysfunction to drive caspase3/GSDME-mediated cancer cell pyroptosis. Cell Cycle 19 (10), 1089–1104. 10.1080/15384101.2020.1743911 32286137 PMC7217368

[B483] Zheng Z. Li G. (2020). Mechanisms and therapeutic regulation of pyroptosis in inflammatory diseases and cancer. Int. J. Mol. Sci. 21 (4), 1456. 10.3390/ijms21041456 32093389 PMC7073143

[B484] Zhou J. Li G. Han G. Feng S. Liu Y. Chen J. (2020). Emodin induced necroptosis in the glioma cell line U251 via the TNF-α/RIP1/RIP3 pathway. Invest. New Drugs 38 (1), 50–59. 10.1007/s10637-019-00764-w 30924024 PMC6985083

[B485] Zhou J. Yu Q. Song J. Li S. Li X. L. Kang B. K. (2023). Photothermally triggered copper payload release for cuproptosis-promoted cancer synergistic therapy. Angew. Chem. Int. Ed. Engl. 62 (12), e202213922. 10.1002/anie.202213922 36585379

[B486] Zhou Q. Meng Y. Li D. Yao L. Le J. Liu Y. (2024a). Ferroptosis in cancer: from molecular mechanisms to therapeutic strategies. Signal Transduct. Target Ther. 9 (1), 55. 10.1038/s41392-024-01769-5 38453898 PMC10920854

[B487] Zhou Y. Li J. Xu X. Zhao M. Zhang B. Deng S. (2019). (64)Cu-based radiopharmaceuticals in molecular imaging. Technol. Cancer Res. Treat. 18, 1533033819830758. 10.1177/1533033819830758 30764737 PMC6378420

[B488] Zhou Y. Liu L. Tao S. Yao Y. Wang Y. Wei Q. (2021). Parthanatos and its associated components: promising therapeutic targets for cancer. Pharmacol. Res. 163, 105299. 10.1016/j.phrs.2020.105299 33171306

[B489] Zhou Y. Manghwar H. Hu W. Liu F. (2022). Degradation mechanism of autophagy-related proteins and research progress. Int. J. Mol. Sci. 23 (13), 7301. 10.3390/ijms23137301 35806307 PMC9266641

[B490] Zhou Y. Tao L. Qiu J. Xu J. Yang X. Zhang Y. (2024b). Tumor biomarkers for diagnosis, prognosis and targeted therapy. Signal Transduct. Target Ther. 9 (1), 132. 10.1038/s41392-024-01823-2 38763973 PMC11102923

[B491] Zimna A. Kurpisz M. (2015). Hypoxia-inducible factor-1 in physiological and pathophysiological angiogenesis: applications and therapies. Biomed. Res. Int. 2015, 549412. 10.1155/2015/549412 26146622 PMC4471260

